# Supplementary material for: Faecal calprotectin to detect inflammatory bowel disease: a systematic review and exploratory meta-analysis of test accuracy
Source: BMJ Open. 2019 Mar 8;9(3):e027428. doi: 10.1136/bmjopen-2018-027428 (PMC6429840; doi:10.1136/bmjopen-2018-027428)
Supplement: Supplementary data [file bmjopen-2018-027428supp001.pdf]

Supplement

Supplement 1 Search strategy

Ovid MEDLINE(R) 1946 to May Week 3 2017 (searched on 31/05/2017)
472 references exported

1.	exp Inflammatory Bowel Diseases/di [Diagnosis]	10670
2.	exp Irritable Bowel Syndrome/di [Diagnosis]	1303
3.	Crohn* disease.tw.	34506
4.	Ulcerative colitis.tw.	29458
5.	Inflammatory bowel disease.tw.	27920
6.	Irritable bowel syndrome.tw.	9092
7.	(IBS or IBD).tw.	20353
8.	(chronic diarrhoea or chronic diarrhea).tw.	3608
9.	(abdominal pain or abdominal discomfort).tw.	42241
10.	1 or 2 or 3 or 4 or 5 or 6 or 7 or 8 or 9	124837
11.	Calprotectin.tw.	1463
12.	exp Leukocyte L1 Antigen Complex/an [Analysis]	606
13.	11 or 12	1594
14.	10 and 13	752
15.	Limit 14 to ed=20120901-20170531	472

Ovid MEDLINE(R) Daily Update May 30, 2017, Ovid MEDLINE(R) In-Process & Other Non-Indexed Citations May 30, 2017, Ovid MEDLINE(R) Epub Ahead of Print May 30, 2017 (searched on 31/05/2017)
240 references exported

1.	exp Inflammatory Bowel Diseases/di [Diagnosis]	15
2.	exp Irritable Bowel Syndrome/di [Diagnosis]	3
3.	Crohn* disease.tw.	4163
4.	Ulcerative colitis.tw.	3292
5.	Inflammatory bowel disease.tw.	4537
6.	Irritable bowel syndrome.tw.	1568
7.	(IBS or IBD).tw.	4167
8.	(chronic diarrhoea or chronic diarrhea).tw.	332
9.	(abdominal pain or abdominal discomfort).tw.	7253
10.	1 or 2 or 3 or 4 or 5 or 6 or 7 or 8 or 9	17922
11.	Calprotectin.tw.	435
12.	exp Leukocyte L1 Antigen Complex/an [Analysis]	3
13.	11 or 12	435
14.	10 and 13	240

Embase 1974 to 2017 May 30 (searched on 31/05/2017)
1995 references exported

1.	crohn* disease.tw.	58919
2.	ulcerative colitis.tw.	47316
3.	inflammatory bowel disease*.tw.	56906
4.	irritable bowel syndrome*.tw.	16123
5.	(IBD or IBS).tw.	45994
6.	(chronic diarrhoea or chronic diarrhea).tw.	5437
7.	(abdominal pain or abdominal discomfort).tw.	77520
8.	1 or 2 or 3 or 4 or 5 or 6 or 7	213302
9.	calprotectin.tw.	3698
10.	exp calgranulin/ec [Endogenous Compound]	2298
11.	9 or 10	4798
12.	8 and 11	2731
13.	exp Crohn disease/di [Diagnosis]	9738
14.	exp ulcerative colitis/di [Diagnosis]	6189
15.	13 or 14	13053
16.	11 and 15	347
17.	12 or 16	2747
18.	(fecal or faecal).tw.	82659
19.	17 and 18	2159
20.	limit 19 to dd=20120901-20170531	1487
21.	limit 19 to em=201235-201722	1975
22.	20 or 21	1995

Cochrane library (search on 31/05/2017)

calprotectin and (inflammatory bowel disease* or irritable bowel syndrome or crohn* disease or ulcerative colitis) - 158 hits
Other reviews: 6
Trials: 147
Technology assessments: 3
Economic evaluations: 2 (not of interest)

WoS: Science Citation Index and Conference Proceedings Citation Index 2012-2017 (search on 31/05/2017)

TS=(calprotectin and (inflammatory bowel disease* or irritable bowel syndrome or crohn* disease or ulcerative colitis)) – 1,063 hits

Supplement 2

Data extraction form: faecal calprotectin

First reviewer: KF Second reviewer: HF

**Study details**	
Study ID (Endnote ref)	
First author surname	
Year of publication	
Country	
Study setting	
Number of centres	
Duration of study	
Funding	
**Aim of the study**	
**Description of study format (study design / set up)**	
**Inclusion/exclusion criteria for patients**	
Inclusion criteria:	
Exclusion criteria:	
**Participants**	
**Item**	
Definition of included patients (primary care, referred, secondary care)	
Presentation (indication for FC testing)	
Previous investigations	
Total number of patients (samples) at baseline	
N excluded and reason	
N included in study	
N included in analysis	
Age mean (range)	
Adults (>60 years) n (%)	
Young adults (<18 years) n (%)	
Male n (%)	
Female n (%)	
**Index test**	
Test type	
Range in µg/g	
Cut-off in µg/g	
Cut-off pre-specified yes/no	
Time between taking the sample and freezing it	
Time between index test and reference standard	
Blinding to reference standard results	
Describe how index test was performed	
Reference standard	
Define patients and proportion who received reference standard 1	
Define patients and proportion who received reference standard 2	
Define patients and proportion who received no reference standard	
Blinding to index test results	
**Outcomes**	
Define differential diagnosis that the 2x2 applies to	
Definition of target condition (i.e. inclusion of conditions in organic category)	
Definition of IBS / functional disease	

Record test accuracy measures and 2x2 tables for all cut-offs and tests	
Proportion of patients referred if primary care population	
Proportion having received colonoscopy if primary care population	
Proportion of colonoscopies with abnormal findings n/N (%)	
**Authors' conclusion**	
**Reviewer's notes**	

Supplement 3 Between study variance of logit sensitivity and logit specificity of each random effects meta-analysis (for tests and clinical questions)

There was some statistical evidence (Chi-square = 14.145, 9df, P=0.1173) that the assumption of equal variances for tests is reasonable at the significance level of 0.05. However, the number of studies per test was small, and examination of the variances of the tests revealed that there is a substantial difference in the between-study variance of logit sensitivity and logit specificity of each test. The model did not converge when unequal variances were assumed between test types.

For clinical questions there was some statistical evidence (Chi-square = 13.328, 6df, P=0.03812) that the assumption of equal variance may not be reasonable for the 50µg/g cut-off, but reasonable for the 100µg/g cut-off (Chi-square = 2.3185, 6df, P=0.8882) with a much smaller number of studies. However, the models with non-equal variances did not converge, so results should be interpreted with caution.

**Test name**	**Between study variance (logitSe)**	**Between study variance (logitSp)**	**Correlation between logitSe and logitSp**
PhiCal	1.301	1.202	-0.94
EK-CAL	0.506	1.265	-0.47
Quantum-Blue	2.2012	0.1378	1.00
ELiA	0.9620	0.6593	0.132
**Clinical questions**			
IBD vs IBS	1.4131	0.4883	-0.81
IBD vs non-IBD	1.1198	0.4731	-0.81
Organic vs non-organic disease	0.1546	0.7890	0.48

logitSe logit sensitivity, logitSp logit specificity, IBD inflammatory bowel disease, IBS irritable bowel syndrome

Supplement 4 Excluded studies with reason

Studies excluded from new review

Reference of full text	Reason for exclusion
1. Anonymous. What is the faecal calprotectin test? Drug & Therapeutics Bulletin. 2014;52(9):102-4.	Review
2. Bai W, Boswell T. Clinical utility and outcome analysis of faecal calprotectin in Hawkes Bay District Health Board. New Zealand Medical Journal. 2016;129(1433):69-73.	No 2x2 data, 46/85 patients with FC test for differential diagnosis (IBD versus non-IBD) had no final diagnosis reported, FP only partially reported for patients with IBS diagnosis, not for non-IBD diagnoses, FN for IBD not reported
3. Bar-Gil Shitrit A, Koslowsky B, Livovsky DM, Shitrit D, Paz K, Adar T, Adler SN, Goldin E. A prospective study of fecal calprotectin and lactoferrin as predictors of small bowel Crohn's disease in patients undergoing capsule endoscopy. Scandinavian Journal of Gastroenterology. 2017;52(3):328-33.	Small bowel only, patient spectrum - all patients included had previous negative colonoscopy
4. Burri E, Manz M, Rothen C, Rossi L, Beglinger C, Lehmann FS. Monoclonal antibody testing for fecal calprotectin is superior to polyclonal testing of fecal calprotectin and lactoferrin to identify organic intestinal disease in patients with abdominal discomfort. Clinica Chimica Acta. 2013;416:41-7.	Overlap with previous SR
5. Burri E, Manz M, Schroeder P, Froehlich F, Rossi L, Beglinger C, Lehmann FS. Diagnostic yield of endoscopy in patients with abdominal complaints: incremental value of faecal calprotectin on guidelines of appropriateness. BMC Gastroenterology. 2014;14:57.	2x2 data is for risk of significant endoscopy finding by EPAGE alone versus EPAGE plus FC (not FC versus actual endoscopy finding)
6. Chang MH, Chou JW, Chen SM, Tsai MC, Sun YS, Lin CC, Lin CP. Faecal calprotectin as a novel biomarker for differentiating between inflammatory bowel disease and irritable bowel syndrome. Molecular Medicine Reports. 2014;10(1):522-6.	Patients with confirmed IBD/ IBS and healthy controls included, no 2x2 data, sensitivity and specificity and predictive values reported for 'optimal' cut off from ROC for IBD patients only
7. Chapman TP, Chen LY, Leaver L. Investigating young adults with chronic diarrhoea in primary care. BMJ. 2015;350:h573.	Case study, report on best clinical practice
8. Egea Valenzuela J, Pereniguez Lopez A, Perez Fernandez V, Alberca de Las Parras F, Carballo Alvarez F. Fecal calprotectin and C-reactive protein are associated with positive findings in capsule endoscopy in suspected small bowel Crohn's disease. Revista Espanola de Enfermedades Digestivas. 2016;108(7):394-400.	Small bowel only, spectrum bias - all patients included had previous negative colonoscopy
9. Egea-Valenzuela J, Alberca-de-Las-Parras F, Carballo-Alvarez F. Fecal calprotectin as a biomarker of inflammatory lesions of the small bowel seen by videocapsule endoscopy. Revista Espanola de Enfermedades Digestivas. 2015;107(4):211-4.	Small bowel only, spectrum bias - all patients included had previous negative colonoscopy
10. Elias SG, Kok L, de Wit NJ, Witteman BJ, Goedhard JG, Romberg-Camps MJ, Muris JW, Moons KG. Is there an added value of faecal calprotectin and haemoglobin in the diagnostic work-up for primary care patients suspected of significant colorectal disease? A cross-sectional diagnostic study. BMC Medicine. 2016;14(1):141.	Risk prediction model modelling the incremental diagnostic accuracy of FC test in addition to physical examination predictors
11. Emmanuel A, Landis D, Peucker M, Hungin AP. Faecal biomarker patterns in patients with symptoms of irritable bowel syndrome. Frontline Gastroenterology. 2016;7(4):275-82.	FC for characterisation of demographics and IBS subtypes in patients with known IBS
12. Garcia F, Martinez C, Juliao H, Bautista-Molano W, Valle-Onate R, Rueda JC, Romero-Sanchez C. Autoantibodies and fecal calprotectin levels in a group of Colombian patients with inflammatory bowel disease. Gazzetta Medica Italiana Archivio per le Scienze Mediche. 2017;176(3):132-41.	IBD patients only, IBS excluded, not test accuracy but characterisation of severity of IBD patients
13. Hale MF, Drew K, McAlindon ME, Sidhu R. The diagnostic accuracy of faecal calprotectin and small bowel capsule endoscopy and their correlation in suspected isolated small bowel Crohn's disease. European Journal of Gastroenterology & Hepatology. 2016;28(10):1145-50.	Small bowel only, unlcear whether included patients had normal colonoscopy
14. Hoog CM, Bark LA, Brostrom O, Sjoqvist U. Capsule endoscopic findings correlate with fecal calprotectin	Patients included have had negative colonoscopy and positive capsule endoscopy, small bowel only

	and C-reactive protein in patients with suspected small-bowel Crohn's disease. Scandinavian Journal of Gastroenterology. 2014;49(9):1084-90.	
15.	Jensen MD, Nathan T, Rafaelsen SR, Kjeldsen J. Ileoscopy reduces the need for small bowel imaging in suspected Crohn's disease. Danish Medical Journal. 2012;59(9):A4491.	Comparison is ileocolonoscopy versus ileocolonoscopy plus small bowel capsule endoscopy
16.	Joosen AMCP, Kok MB, Van Der Linden IJM, Bozkurt Z, Broos H, Van Pelt J, Van Heerde M, De Groot MJM. Analytical and clinical evaluation of faecal calprotectin. [Dutch] Analytische en klinische evaluatie van de bepaling van calprotectine in feces. Nederlands Tijdschrift voor Klinische Chemie en Laboratoriumgeneeskunde. 2013;38(4):196-201.	not sufficient data for 2x2 table and no demographics about patients, don't know whether adults and/or children, only reports PPV, NPV and concordance (TP+TN/N)
17.	Kalantari H, Taheri A, Yaran M. Fecal calprotectin is a useful marker to diagnose ulcerative colitis from irritable bowel syndrome. Advanced Biomedical Research. 2015;4:85.	UC patients only, 21 patients with other colonoscopy findings (Crohn's colitis, microscopic colitis, collagenous colitis, or other) were excluded
18.	Kok L, Elias SG, Witteman BJM, Goedhard JG, Muris JWM, Moons KGM, De Wit NJ. Diagnostic accuracy of point-of-care fecal calprotectin and immunochemical occult blood tests for diagnosis of organic bowel disease in primary care: The cost-effectiveness of a decision rule for abdominal complaints in primary care (CEDAR) study. Clinical Chemistry. 2012;58(6):989-98.	Overlap with previous SR
19.	Kopylov U, Yung DE, Engel T, Avni T, Battat R, Ben-Horin S, Plevris JN, Eliakim R, Koulaouzidis A. Fecal calprotectin for the prediction of small-bowel Crohn's disease by capsule endoscopy: a systematic review and meta-analysis. European Journal of Gastroenterology & Hepatology. 2016;28(10):1137-44.	Small bowel only, mix of patients with suspected and confirmed IBD, 5/7 studies investigated patients with negative endoscopy
20.	Kotze LM, Nisihara RM, Marion SB, Cavassani MF, Kotze PG. FECAL CALPROTECTIN: levels for the ethiological diagnosis in Brazilian patients with gastrointestinal symptoms. Arquivos de Gastroenterologia. 2015;52(1):50-4.	No 2x2 data, number of IBD and IBS reported but FC levels only reported as means and medians, no measures of accuracy reported to derive 2x2 data from, data requested – no response
21.	Koulaouzidis A, Sipponen T, Nemeth A, Makins R, Kopylov U, Nadler M, Giannakou A, Yung DE, Johansson GW, Bartzis L, Thorlacius H, Seidman EG, Eliakim R, Plevris JN, Toth E. Association Between Fecal Calprotectin Levels and Small-bowel Inflammation Score in Capsule Endoscopy: A Multicenter Retrospective Study. Digestive Diseases & Sciences. 2016;61(7):2033-40.	Small bowel only, mix of suspected and confirmed IBD
22.	Lance S, White C. An audit on the appropriate use of faecal calprotectin testing within the Taranaki DHB: a case for a more discerning approach. New Zealand Medical Journal. 2015;128(1417):24-9.	Not sufficient information to derive 2x2 data
23.	Li LQ, Zeng J, Wang S, Chen X, Jiang Z. Fecal calprotectin for diagnosis of inflammatory bowel disease: A meta-analysis. [Chinese]. World Chinese Journal of Digestology. 2016;24(31):4272-8.	Meta-analysis in Chinese, different question as included studies enrolled patients with confirmed diagnosis and healthy controls
24.	Licata A, Randazzo C, Cappello M, Calvaruso V, Butera G, Florena AM, Peralta S, Camma C, Craxi A. Fecal calprotectin in clinical practice: a noninvasive screening tool for patients with chronic diarrhea. Journal of Clinical Gastroenterology. 2012;46(6):504-8.	Overlap with previous SR
25.	Lozoya Angulo ME, de Las Heras Gomez I, Martinez Villanueva M, Noguera Velasco JA, Aviles Plaza F. Faecal calprotectin, an useful marker in discriminating between inflammatory bowel disease and functional gastrointestinal disorders. Gastroenterologia y Hepatologia. 2017;40(3):125-31.	Adults and children, results reported combined, author is unable to send results for adults separately
26.	Manz M, Burri E, Rothen C, Tchanguizi N, Niederberger C, Rossi L, Beglinger C, Lehmann FS. Value of fecal calprotectin in the evaluation of patients with abdominal discomfort: An observational study. BMC Gastroenterology. 2012:5.	Overlap with previous SR
27.	McFarlane M, Chambers S, Malik A, Lee B, Sung E, Nwokolo C, Waugh N, Arasaradnam R. Clinical outcomes at 12 months and risk of inflammatory bowel disease in patients with an intermediate raised fecal	Included patients with intermediate FC outcomes only

	calprotectin: a 'real-world' view. BMJ Open. 2016;6(6):e011041.	
28.	McFarlane M, Chambers S, Malik A, Lee B, Sung E, Nwokolo C, Waugh N, Arasaradnam R. Is NICE too optimistic about savings from normal faecal calprotectin results? Journal of Gastroenterology and Hepatology Research. 2016;5(1):1895-8.	Included patients with FC negative results only
29.	Menees SB, Powell C, Kurlander J, Goel A, Chey WD. A meta-analysis of the utility of C-reactive protein, erythrocyte sedimentation rate, fecal calprotectin, and fecal lactoferrin to exclude inflammatory bowel disease in adults with IBS. American Journal of Gastroenterology. 2015;110(3):444-54.	Patients with confirmed IBD
30.	Olsen PAS, Fossmark R, Qvigstad G. Fecal calprotectin in patients with suspected small bowel disease - a selection tool for small bowel capsule endoscopy? Scandinavian Journal of Gastroenterology. 2015;50(3):272-7.	Small bowel only, patients had negative / inconclusive bowel investigation
31.	Osipenko MF, Livzan m A, Skalinskaia MI, Lialiukova EA. [Fecal calprotectin concentration in the differential diagnosis of bowel diseases]. Terapevticheskii Arkhiv. 2015;87(2):30-3.	Not sufficient information to derive 2x2 data
32.	Polewiczowska B, Ashraf W, Grainger S, Khan SR, Sandhu K. Negative faecal calprotectin (FC) is a strong predictor of negative small bowel capsule endoscopy. United European Gastroenterology Journal. 2013;1):A188-A9.	Small bowel only, not enough information on included patients
33.	Rodriguez-Moranta F, Lobaton T, Rodriguez-Alonso L, Guardiola J. [Fecal calprotectin in the diagnosis of inflammatory bowel diseases]. Gastroenterologia y Hepatologia. 2013;36(6):400-6. Calprotectina fecal en el diagnostico de enfermedades inflamatorias.	Review
34.	Samuel S, Ragunath K. Colonoscopy, inflammatory bowel disease. Endoscopy. 2013;45(4):289-91.	Review
35.	Schulz C, Wex T, Arnim UV, Malfertheiner P. Validation of Two Calprotectin Rapid Tests in Daily Routine. Clinical Laboratory. 2016;62(7):1249-54.	Patients had confirmed IBD / IBS diagnosis, reference standard was ELISA for test comparison
36.	Seenan JP, Thomson F, Rankin K, Smith K, Gaya DR. Are we exposing patients with a mildly elevated faecal calprotectin to unnecessary investigations? Frontline Gastroenterology. 2015;6(3):156-60.	Only patients with 100-200 microgramm/g FC characterised
37.	Sipponen T, Haapamaki J, Savilahti E, Alfthan H, Hamalainen E, Rautiainen H, Koskenpato J, Nuutinen H, Farkkila M. Fecal calprotectin and S100A12 have low utility in prediction of small bowel Crohn's disease detected by wireless capsule endoscopy. Scandinavian Journal of Gastroenterology. 2012;47(7):778-84.	Overlap with previous SR - excluded as only investigation of small bowel
38.	Smith LA, Gaya DR. Utility of faecal calprotectin analysis in adult inflammatory bowel disease. World Journal of Gastroenterology. 2012;18(46):6782-9.	Review
39.	Sood R, Gracie DJ, Law GR, Ford AC. Systematic review with meta-analysis: the accuracy of diagnosing irritable bowel syndrome with symptoms, biomarkers and/or psychological markers. Alimentary Pharmacology & Therapeutics. 2015;42(5):491-503.	Not FC
40.	Sydora MJ, Sydora BC, Fedorak RN. Validation of a point-of-care desk top device to quantitate fecal calprotectin and distinguish inflammatory bowel disease from irritable bowel syndrome. Journal of Crohns & Colitis. 2012;6(2):207-14.	Overlap with previous SR - excluded as patients had known diagnosis prior to the study
41.	Turvill J. High negative predictive value of a normal faecal calprotectin in patients with symptomatic intestinal disease. Frontline Gastroenterology. 2012;3(1):21-8.	Overlap with previous SR
42.	von Arnim U, Wex T, Ganzert C, Schulz C, Malfertheiner P. Fecal calprotectin: a marker for clinical differentiation of microscopic colitis and irritable bowel syndrome. Clinical & Experimental Gastroenterology. 2016;9:97-103.	Spectrum bias - only included microscopic colitis patients (mixed active and in remission), no 2x2 data
43.	Wagner M, Sjoberg K, Vigren L, Olesen M, Benoni C, Toth E, Carlson M. Elevated fecal levels of eosinophil granule proteins predict collagenous colitis in patients referred to colonoscopy due to chronic non-bloody	Not sufficient information to derive 2x2 data

	diarrhea. Scandinavian Journal of Gastroenterology. 2016;51(7):835-41.	
44.	Wang S, Shi H, Lu H, Wang F, Wei J, Wang Z, Liu C, Yuan B. Significance of fecal calprotectin in differential diagnosis of whole spectrum of digestive system diseases. [Chinese]. Chinese Journal of Gastroenterology. 2012;17(4):237-9.	Included patients had confirmed disease
45.	Wang S, Wang Z, Shi H, Heng L, Juan W, Yuan B, Wu X, Wang F. Faecal calprotectin concentrations in gastrointestinal diseases. Journal of International Medical Research. 2013;41(4):1357-61.	no 2x2 data, sens / spec reported for 2 cut-offs from ROC, but no predictive value – request for further data failed, e-mail bounced back
46.	Wassell J, Wallage M, Brewer E. Evaluation of the Quantum Blue rapid test for faecal calprotectin. Annals of Clinical Biochemistry. 2012;49(1):55-8.	Overlap with previous SR - excluded as no patient details reported

Abstracts

1.	Alzoubaidi D, Asser L, Price T, Lithgo K, Housley D, Johnson MW. Is a false positive faecal calprotectin as false as you think? Gut. 2015;64:A238.	only considered FC+ patients with normal endoscopy
2.	Astle VI, Lewis NR. Under-utilisation of faecal calprotectin to exclude ibd in patients with functional bowel disorders. Gut. 2014;63:A207-A8.	Cannot derive meaningful 2x2 data
3.	Atef S, Abdel Rahman S. The diagnostic performance of the combined use of fecal calprotectin and occult blood tests for screening of organic bowel disease in primary care setting. Biochimica Clinica. 2013;37:S658.	No test and cut off reported
4.	Aujla UI, Hayee B, Sherwood R, Chung-Faye G. Predictive value of faecal calprotectin in patients undergoing colonoscopy: A cost-effective analysis. United European Gastroenterology Journal. 2013;1):A517.	Cannot derive meaningful 2x2 table
5.	Caccaro R, Lollo G, Hatem G, Ugoni A, Buda A, D'Odorico A, Galeazzi F, D'Inca R, Savarino EV, Sturniolo GC. Are non-invasive markers of gastro-intestinal disease predictors of enteropathy at small bowel capsule endoscopy? United European Gastroenterology Journal. 2014;1):A347-A8.	small bowel only in colonoscopy negative patients
6.	Conroy S, Hale M, Cross S, Swallow K, Sidhu R, Sargur R, et al. Does the performance of faecal calprotectin testing in primary care differentiate patients with inflammatory bowel disease? Gut. 2015;64:A25.	Patients are children and adults no proportions reported
7.	Demir OM, Ahmed Z, Logan RPH. Optimising the use of faecal calprotectin for early diagnosis of IBD in primary care. Journal of Crohn's and Colitis. 2013;7:S8-S9.	Cannot derive meaningful 2x2 data
8.	Digby J, Steele RJC, Strachan JA, Mowat C. Stool tests can potentially rule out significant bowel disease in symptomatic patients in primary care. United European Gastroenterology Journal. 2014;1):A47.	Overlap of study population with Mowat 2016 suspected
9.	Eccles J, Neely A, Lynch M, Ferguson CB, Morrison G. An overview of the impact of fecal calprotectin testing in the management of patients within the gastroenterology outpatient clinic in a general hospital. Gastroenterology. 2014;1):S-798.	Over 30% are children 15-18 years
10.	Greig E, Gore S, Staveley K, Phillips I, Benneyworth R, Matull R, Thole S, Williams M. Delivering cost effective management for Irritable Bowel Syndrome (IBS) across Somerset. Gut. 2015;64:A192-A3.	Known cases of IBD and new cases included, proportions not reported
11.	Hale MF, Drew K, McAlindon ME, Lobo AJ, Sidhu R. Faecal calprotectin in patients with suspected small bowel Crohn's disease: Correlation with small bowel capsule endoscopy. Gut. 2015;64:A83.	Colonoscopy negative patients only, investigation of small bowel only
12.	Hunt N, Allcock R, Sharma A, Myers M. Diagnostic performance of faecal calprotectin in primary care. Gut. 2014;63:A159.	Not enough information for 2x2 table
13.	Kotze L, Nisihara R, Cavassani M, Valarini S, Kotze P. Fecal calprotectin levels for the ethiological diagnosis in Brazilian patients with gastrointestinal symptoms. American Journal of Gastroenterology. 2014;109:S500.	Superseeded by full text, not enough data for 2x2 table, numbers of IBD and IBS don't add up to total, no numbers for FC levels or test accuracy measures
14.	Kwok R, Peter F, Page BP, Ahmed T, Tay DXH, Woo ASJ, Constantinos A, Yip BCH, Sze KCP, Wee EWL. Diagnostic performance of faecal calprotectin in Singaporean patients. Journal of Gastroenterology and Hepatology (Australia). 2016;31:212.	No 2x2 data, don't know definition of non-colitis group

15.	Keny B, Gaikwad S, Parkar S, Dherai AJ, Shetty D, Desai D, Joshi A, Abraham P, Gupta T, Tester Ashavaid F. Fecal calprotectin-marker for inflammatory bowel disease. Indian Journal of Clinical Biochemistry. 2016;31 (1 Supplement 1):S59.	Cannot derive meaningful 2x2 table
16.	Lee S, Borthwick H, Dhar A. Faecal calprotectin testing in primary and secondary care Are the current manufacturer's cut-off values clinically useful? Journal of Crohn's and Colitis. 2014;8:S155-S6.	Superseded by full text Lee 2013
17.	Maheshwari P, Junagade P, Goulding C. Evaluation of the use of fecal calprotectin as a diagnostic aid for IBD in an Irish population. Journal of Crohn's and Colitis. 2014;8:S159.	Cannot derive meaningful 2x2 table
18.	Malik A, Bowen D, Rees I. Usefulness of fecal calprotectin in clinical practice in a district general hospital. Gut. 2013;62:A65.	Duplicate abstract with Alrubaiy 2012
19.	McFarlane M, Chambers S, Dhaliwal A, Patel A, Nwokolo C, Arasaradnam R. Six month clinical outcomes in patients with intermediate raised faecal calprotectin levels. International Journal of Surgery. 2015;23:S48.	intermediate FC levels only
20.	McFarlane M, Dhaliwal A, Chambers S, Nwokolo C, Patel A, Arasaradnam R. Clinical outcomes in patients with intermediate raised faecal calprotectin levels. United European Gastroenterology Journal. 2014;1):A371-A2.	Not sufficient data for 2x2
21.	Mishreki A, Bell H, Austin C, Sheppard S, Noblett S. Does a raised faecal calprotectin level correlate with a diagnosis of IBD on colonoscopic biopsies? Colorectal Disease. 2014;16:43.	No test and cut-off reported, clinical question not specified, differential diagnosis unknown
22.	Mohammed N, Smale S. Positive calprotectin but negative investigations-what next? Gut. 2012;61:A236.	positive faecal calprotectin with normal endoscopic/radiological only
23.	Moroni F, Winter JW, Morris AJ, Gaya DR. What is the clinical relevance of a mildly elevated faecal calprotectin detected in new referrals to the gastroenterology clinic? Gut. 2012;61:A78-A9.	only low and intermediate FC group included
24.	Mukhtar A, Sivaramakrishnan N, Hassan F. Should the cut off values of faecal calprotectin for initiating further investigations be higher than current practice? Gut. 2016;65:A243-A4.	Only FC positive patients included
25.	Oyaert M, Trouve C, Baert F, De Smet D, Langlois M, Vanpoucke H. Performance characteristics of faecal calprotectin testing for diagnosis of Inflammatory Bowel Disease. Acta Clinica Belgica. 2014;69:1-2.	Superseded by full publication
26.	Pantaleoni S, Touscoz GA, Caviglia GP, Adriani A, Sguazzini C, Sapone N, Reggiani S, Rizzetto M, Astegiano M. Fecal calprotectin is an effective diagnostic tool that differentiates pathological from functional intestinal disorders. Digestive and Liver Disease. 2013;45:S104-S5.	patients with known diagnosis
27.	Parker C, Lamb CA, Robinson M, Mansfield JC, Gunn M. Predicting inflammatory pathology at capsule enteroscopy: What is the utility of a raised faecal calprotectin? Gut. 2015;64:A74-A5.	excluded only FC positive patients
28.	Patel KV, Zaman S, Fong S, Anderson SH. Comparison of video capsule endoscopy and faecal calprotectin as diagnostic tools in patients with abdominal symptoms suggestive of small bowel Crohn's Disease. Journal of Crohn's and Colitis. 2015;9:S153-S4.	small bowel only
29.	Reed O, Doyle J, Murphy S. Faecal calprotectin to screen out IBS: Is it being used correctly? Irish Journal of Medical Science. 2015;184 (6 Supplement 1):S248.	incomplete data at time of analysis
30.<br>31.	Rouke JO, Dhaliwal A, Sagar V, Davies J, Milestone A. Use of faecal calprotectin in primary care to distinguish irritable bowel syndrome from inflammatory bowel disease. Gut. 2015;64:A86-A7. And<br>Dhaliwal A, J OR, Sagar V, Burdsall J, Ransford R, Milestone A. Use of faecal calprotectin pathway in primary care to distinguish irritable bowel syndrome from inflammatory bowel disease. United European Gastroenterology Journal. 2015;1):A237.	Identical, incomplete data available at time of analysis
32.	Sartain S, Stone A. Faecal calprotectin levels of 50-200: Endoscopic findings and subsequent diagnoses. Gut. 2016;65:A197-A8.	Only intermediate FC levels
33.	Shastri YM, Povse N, Stein J. Prospective evaluation of faecal tumour pyruvate kinase type M2 (M2-PK) in	IBD monitoring

	comparison to calprotectin in IBD patients. Journal of Crohn's and Colitis. 2013;7:S75-S6.	
34.	Shitrit AB, Braverman D, Paz K, Adar T, Koslowsky B, Goldin E. Fecal calprotectin and lactoferrin as biomarkers in patients undergoing capsule endoscopy. Gastroenterology. 2013;1):S424.	Small bowel only
35.	Taylor N, Hills E, Sheen C, Al-Bahrani A, Grellier L. An audit of faecal calprotectin testing in suspected inflammatory bowel disease in the under 45's. Journal of Crohn's and Colitis. 2013;7:S131.	Clinical question not specified, non-IBD group unknown
36.	Tomkins C, Zeino Z, Nwokolo C, Smith SC, Arasaradnam R. Faecal calprotectin analysis: Does the method matter? Gut. 2012;61:A173-A4.	No meaningful 2x2 data
37.	Wu J, Bolton L, Chapman C, Chey CS, Harrison E, Kinderman H, Johnson H, Richards-Taylor A, Weaver S, McLaughlin S. Faecal calprotectin has an acceptable sensitivity for detecting small bowel crohn's disease: Results from real world clinical practice. Gut. 2016;65:A145.	Known diagnosis and small bowel only
38.	Zaman S, Patel K, Goel R, Borrow DM, Anderson SH. Comparison of video capsule endoscopy and faecal calprotectin as diagnostic tools in patients with abdominal symptoms suggestive of small bowel crohn's disease. United European Gastroenterology Journal. 2013;1):A493.	Small bowel only
39.	Zuhra N, Sartain S, Gordon J, Lloyd D. Capsule endoscopy in the investigation and management of small bowel crohn's disease. Gut. 2016;65:A167-A8.	Small bowel only, incomplete FC data

Studies from previous review (Waugh et al. 2012)

1.	Ashorn S, Honkanen T, Kolho KL, Ashorn M, Valineva T, Wei B, et al. Fecal calprotectin levels and serological responses to microbial antigens among children and adolescents with inflammatory bowel disease. Inflamm Bowel Dis 2009;15:199–205.	Children
2.	Basumani P, Bardhan K, Eyre R, Ellis R, The Rotherham Team. Faecal calprotectin: Rotherham experience (slide presentation). BSG Away day 28/06/2012 (accessed 19 July 2013). Unpublished	Published and included as Banerjee 2015
3.	Canani RB, de Horatio LT, Terrin G, Romano MT, Miele E, Staiano A, et al. Combined use of noninvasive tests is useful in the initial diagnostic approach to a child with suspected inflammatory bowel disease. JPGN 2006;42:9–15.	Children
4.	Diamanti A, Panetta F, Basso MS, Forgione A, Colistro F, Bracci F, et al. Diagnostic work-up of inflammatory bowel disease in children: the role of calprotectin assay. Inflamm Bowel Dis 2010;16:1926–30.	Children
5.	Dolwani S, Metzner M, Wassell JJ, Yong A, Hawthorne AB. Diagnostic accuracy of faecal calprotectin estimation in prediction of abnormal small bowel radiology. Aliment Pharmacol Ther. 2004;20(6):615-21.	Small bowel only
6.	Fagerberg UL, Loof L, Myrdal U, Hansson LO, Finkel Y. Colorectal inflammation is well predicted by fecal calprotectin in children with gastrointestinal symptoms. JPGN 2005;40:450–5.	Children
7.	Henderson P, Casey A, Lawrence SJ, Kennedy NA, Kingstone K, Rogers P, et al. The diagnostic accuracy of fecal calprotectin during the investigation of suspected pediatric inflammatory bowel disease. Am J Gastroenterol 2012;107:941–9.	Children
8.	Manz M, Burri E, Rothen C, Tchanguizi N, Niederberger C, Rossi L, et al. Value of fecal calprotectin in the evaluation of patients with abdominal discomfort: an observational study. BMC Gastroenterol. 2012;12:5.	Overlap with Burri 2013
9.	Sidler MA, Leach ST, Day AS. Fecal S100A12 and fecal calprotectin as noninvasive markers for inflammatory bowel disease in children. Inflammatory Bowel Dis 2008;14:359–66.	Children
10.	Tomas AB, Vidal MV, Camps R. Fecal calprotectin as a biomarker to distinguish between organic and	Children

	functional gastrointestinal disease. Rev Esp Enferm Dig 2007;99:689–93.	
11.	Van de Vijver E, Schreuder AB, Cnossen WR, Muller Kobold AC, van Rheenen PF. Safely ruling out inflammatory bowel disease in children and teenagers without referral for endoscopy [published online ahead of print 2012]. Arch Dis Child 2012;12:1014–18.	Children

Supplement 5 Tables of study characteristics

Table of study characteristics - population

Study ID (author, year, country)	Aim	Population (N, setting, age, gender)	Study exclusions	Indication*	Clinical questions	Disease prevalence
Alrubaiy 2012 [1]; UK<br><br>Abstract	To evaluate the diagnostic value of FC as a non-invasive marker of bowel inflammation in routine out-patient gastroenterology clinic.	N=72<br>Setting: out-patients in gastroenterology clinic<br>Mean age: Female: 44 years<br>Male: 47 years<br>Female: 44/72 (61%)	NR	Chronic diarrhoea, abdominal pain, abdominal distension, rectal bleeding	Inflammatory versus non-inflammatory disease	Inflammatory disease (IBD): 2/72 (2.7%)
Banerjee 2015 [2]; UK	To assess the role of FC in aiding differential diagnosis, maximising the numbers in whom IBD could be ruled out, making colonoscopy unnecessary, yet not miss anyone with the disease.	N=119<br>Setting: newly referred patients to gastrointestinal clinic<br>Age:<br>≤40 years: 36%<br>41–60 years: 46%<br>>60 years: 18%<br>Female: 64/119 (54%)	Proven coeliac disease, pancreatic insufficiency	Diarrhoea of longer than 4 weeks duration	IBD versus IBS,<br>Organic versus non-organic disease	IBD: 12/119 (10%)
Bharathi 2005 [3]; UK<br><br>Abstract	To assess the negative predictive value of FC in excluding bowel pathology in young patients with suspected IBS.	N=58<br>Setting: NR<br>Mean age: NR<br>Female: NR	NR	Abdominal pain and/or loose stools	IBD versus IBS	IBD: 0/58
Boyd 2016 [4]; UK<br><br>Abstract	To assess an FC pilot pathway.	N=424<br>Setting: data from a primary care pilot FC pathway<br>Mean age: NR<br>Female: NR	Rejected samples	NR	IBD versus non-IBD	IBD: 23/424 (5.4%)
Burri 2013 [5]; Switzerland	To directly compare the test characteristics of FC and lactoferrin in their ability to detect organic intestinal disease	N=405<br>Setting: patients with abdominal discomfort referred for endoscopy<br>Median age (range): 63 years (18–97)<br>Female: 226 /405(56%)	<18 years	abdominal discomfort	Organic versus other disease	Organic: 143/405 (35%)
Carroccio 2003 [6]; Italy	To evaluate the positive and negative predictive values of the FC assay in identifying the organic causes of chronic diarrhea.	N=70<br>Setting: patients referred for chronic diarrhea to an outpatient gastroenterology clinic<br>Median age (range): 35 years (18–72) Female: 40/70 (57%)	Previous evaluation for chronic diarrhea, overt gastrointestinal bleeding, sigmoidoscopy or colonoscopy during the previous 2 years, familial adenomatous polyposis and hereditary nonpolyposis, colorectal cancer syndrome, pregnancy	Chronic diarrhea of unknown origin, lasting for more than 4 weeks, with or without abdominal pain	IBD versus IBS,<br>IBD versus non-IBD,<br>Organic versus non-organic disease	IBD: 11/70 (16%)

Study ID (author, year, country)	Aim	Population (N, setting, age, gender)	Study exclusions	Indication*	Clinical questions	Disease prevalence
Caviglia 2014 [7]; Italy	To asses FC concentration and evaluate the diagnostic accuracy in patients with and without bowel inflammation	N=66<br>Setting: first time referrals to outpatient gastroenterology clinic<br>Mean age (range): 42 (18-78) years<br>Female: 46/66 (70%)	Regular NSAIDs,<br>known CRC or polyps,<br>known acute gastrointestinal infection (diverticular disease)	Abdominal pain and/or altered bowel habit lasting at least 4 weeks	IBD versus IBS,<br>Inflammatory versus non-inflammatory disease	IBD: 24/66 (36%)
Conroy 2017 [8]; UK	To assess the diagnostic accuracy of FC in the detection of IBD in a primary care setting.	N=410<br>Setting: FC ordered in primary care<br>Median age (range): 42 (16–91) years<br>Female: 248/410 (60%)	< 16 years, known IBD, FC testing initiated in secondary care	NR	IBD versus non-IBD,<br>Organic versus non-organic disease	IBD: 11/410 (2.7%)
Damms 2008 [9]; Germany	To evaluate the diagnostic accuracy of the new calprotectin rapid test compared to an established ELISA test in detecting colonoscopy-proven intestinal inflammatory diseases and colorectal malignancies.	N=140<br>Setting: Patients referred to colonoscopy as in- or out-patients for clarification of lower GI symptoms<br>Mean age (range): 58 (20–85) years<br>Female: 78/144 (56%)	Known extraintestinal inflammatory diseases such as rheumatoid arthritis, chronic arthritis–sinusitis, urinary tract infection, patients on NSAID or anticoagulants	lower GI symptoms, altered bowel habit, weight loss, by reason of occult blood–rectal bleeding, search of tumor, resectional surgery, follow-up, or routine checkup	IBD versus functional disease,<br>Organic versus non-organic disease	IBD: 18/140 (13%)
De Sloovere 2017 [10]; Belgium	To evaluate two newly developed automated FC immunoassays in terms of analytical and diagnostic performance.	N=229<br>Setting: out- and inpatients with clinical suspicion of IBD and stool sample for FC measurement<br>Median age (range): 34 (15–94) years<br>Female: 150/229 (66%)	<15 years of age, absence of clinical data, unclear diagnosis or insufficient sample volume	Suspicion of IBD	IBD versus IBS,<br>IBD versus non-IBD	IBD: 46/229 (20%)
Dhaliwal 2015 [11]; UK	To determine the ability of FC to exclude IBD and optimal cut-offs for IBD in remission	N=311<br>Setting: FC samples in a secondary care setting<br>Mean age: NR<br>Female: 190/292 (65%)	NR	Suspected functional gastrointestinal disease (IBS) or IBD	IBD versus IBS	IBD: 148/311 (48%)
El-Badry 2010 [12]; Egypt	To evaluate the sensitivity and diagnostic accuracy of FC assay at different cut-off values in discriminating between functional and organic gastrointestinal disorders	N=39<br>Setting: patients referred to hospital for diagnostic differentiation of functional or organic bowel disorders<br>Mean age ±:SD:<br>IBD: 39.4 ± 15.9 years<br>IBS: 34.3 ± 15.75 years	On NSAIDs, aspirin, and/or anticoagulants, concomitant non-gastrointestinal diseases, (rheumatoid arthritis, other connective tissue inflammatory diseases or liver cirrhosis)	abdominal pain, chronic diarrhoea, weight loss and/or anorectal bleeding	IBD versus IBS,<br>IBD vs non-IBD	IBD: 9/39 (23%)

Study ID (author, year, country)	Aim	Population (N, setting, age, gender)	Study exclusions	Indication*	Clinical questions	Disease prevalence
		Organic (non-IBD): 40.2 ± 14 years<br>Female: 19 /39 (49%)				
Garcia 2006 [13]; Spain	To assess the usefulness of FC to predict the presence of pathological colonoscopy and to analyze its use to discriminate between different organic diseases	N=190<br>Setting: patients undergoing colonoscopy for medical indications<br>Mean age±SD: Other: 59.5±6.0 years<br>Polyps: 60.21± 4.53 years<br>CRC: 71.75 ± 6.64 years<br>IBD: 35.84 ±15.4 years<br>Female: 98/190 (52%)	cardiopulmonary disease, kidney or liver disease, celiac disease, known malignancy	NR	Organic versus other disease	Organic disease: 73/190 (38%)
Hogberg 2017 [14]; Sweden	To assess the value of a point of care FIT and FC test in detecting CRC, high risk adenomas HRAs and IBD in primary care.	N=384<br>Setting: FC ordered in primary care<br>Median age: 63.0 years Female: 241/384 (64.6%)	Patients declined, emergency admittance, FC not returned	Abdominal pain, change of bowel habits, diarrhoea, constipation, rectal bleeding, urgency, anaemia, incomplete stool evacuation, weight loss, Melaena, palpable mass	IBD versus non-IBD, Organic versus other disease	IBD: 10/373 (2.6%)
Jang 2016 [15]; Korea	To evaluate the initial diagnostic ability of three faecal calprotectin kits in different bowel diseases	N=41<br>Setting: patients at hospital for workup of abdominal symptoms<br>Mean age ± SD (range):<br>CD patients: 22±5 (15–31)<br>UC patients: 41±21 (19–74)<br>Intestinal BD: 54±21 (39–69)<br>IBS: 17±12 (5–31)<br>Other colitis: 38±24 (11–69)<br>Female: 13/41 (32%)	Incomplete colonoscopy, no faecal samples, CRC, history of bowel resection, uncertain diagnosis after colonoscopy, regular aspirin and/or NSAIDs	abdominal pain, changes in bowel habits, and/or anorectal bleeding	IBD versus IBS	IBD: 31/41 (76%)
Kennedy 2015 [16]; UK	To determine the most effective use of FC in the diagnosis of GI disease in patients with no prior known GI disease, at the first presentation to GI services.	N=895<br>Setting: Data of FC of patients presenting to gastrointestinal services<br>Mean age: 33.1 years (IQR: 25.6–40.7)<br>Female: 581/895 (64.9)	< 16 years or >50 years, Patients with FC tests from other hospitals, patients with confirmed GI diagnosis, on IBD treatment, insufficient detail available, severe intercurrent illness, on aspirin/NSAIDs, on corticosteroids and/or aminosalicylates	Bloody diarrhoea, watery diarrhoea, rectal bleeding, constipation, abdominal pain, weight loss, bloating, vomiting, dyspepsia, fatigue, possible extraintestinal manifestations	IBD versus functional disease,<br>Organic versus non-organic disease	IBD: 91/895 (10%)
Kok 2012 [17]; Netherlands	To quantify the diagnostic accuracy of 3 biomarker tests for the inclusion or exclusion of OBD in patients with persistent	N=382<br>Setting: Referred patients to colonoscopy from primary care (recruitment was taking place at	<18 years old, unable to give informed consent, previously diagnosed with organic disease, or positive on triple	Abdominal pain, rectal bleeding, diarrhoea, fever and weight loss	Organic versus other disease	Organic disease: 99/382 (26%)

Study ID (author, year, country)	Aim	Population (N, setting, age, gender)	Study exclusions	Indication*	Clinical questions	Disease prevalence
	lower-abdomen complaints in primary care, who need colonoscopy referral	the GP practice or straight after registering for endoscopy<br>Median age (range): 60 (18-91) years<br>Female: 209/382 (54.7%)	feces test (TFT), not requiring endoscopy			
Labaere 2014 [18]; Belgium	To evaluate six FC tests and to determine their performance for diagnosis and follow up of IBD patients	N=31<br>Setting: referred for colonoscopy to gastroenterology<br>Mean age: 36 years (16-75)<br>Female: 21/31 (68%)	Inconclusive histopathological and endoscopic evaluation, extremely watery consistency of the stool sample	Chronic diarrhoea and/or abdominal pain, iron deficiency anaemia, unexplained weight loss, or a family history of IBD	IBD versus non-IBD	IBD: 12/31 (39%)
Lee 2014 [19]; UK<br><br>Abstract	To determine whether the manufacturer's cut-off for referral are clinically useful in making a positive diagnosis in patients presenting with chronic diarrhoea.	N=122<br>Setting: FC test results from primary and secondary care<br>Mean age: NR<br>Female: NR	NR	chronic diarrhoea	Organic versus non-organic disease	Organic: 23/122 (19%)
Li 2006 [20]; China	To assess the value of FC in differential diagnosis of IBS.	N=240<br>Setting: outpatients and hospitalised patients undergoing endoscopy<br>Mean age ± SD:<br>IBS: 48±19years<br>Inflammation:42±16 years<br>Female: 121/240 (50%)	Upper gastrointestinal symptoms, stomach/small intestinal disease, diseases of heart, lung, liver, kidney, nerve, mental disorder, alcoholic, pregnant women, drug addiction, long term use of NSAIDs, colorectal adenomas	NR	Inflammatory versus non-inflammatory disease	Inflammation: 60/240 (25%)
Licata 2012 [21]; Italy	To assess the diagnostic performance of FC as a stool-screening biomarker for organic intestinal disease	N=346<br>Setting: outpatients with unexplained chronic, non-bloody diarrhoea referred for colonoscopy<br>Median age (range):<br>Inflammation: 41 (17-80) years<br>No inflammation: 38 (18-87) years<br>Female: 201/346 (58%)	Bleeding, known colorectal or gastric neoplasia, familial adenomatous polyposis and hereditary nonpolyposis colorectal cancer syndrome, history of colonic surgery, recent respiratory or urinary tract infection, acute infectious disease, pregnancy and alcohol abuse, on NSAIDs, aspirin, or anticoagulants within the previous month, CRC	Unexplained chronic (≥4 wk), non-bloody diarrhoea	Inflammatory versus non-inflammatory disease	Inflammation: 142/346 (41%)
Limburg 2000 [22]; USA	To assess and compare calprotectin and hemoglobin (Hb) as stool screening biomarkers for	N=128<br>Setting: Patients referred to colonoscopy	Abnormalities on GI x-rays, bleeding, GI endoscopy performed within preceding 2 weeks, CRC syndrome	Chronic diarrhea (≥4 wk duration) of unknown origin or chronic colitis of unknown activity	Inflammatory versus non-inflammatory disease	Inflammatory disease: 29/128 (23%)

Study ID (author, year, country)	Aim	Population (N, setting, age, gender)	Study exclusions	Indication*	Clinical questions	Disease prevalence
	colorectal inflammation in unexplained chronic diarrhoea	Mean age (range): 57 years (21-85)<br>Female: 84/128 (66%)				
Lizvan 2015 [23]; Russia	To assess the informational content of fecal noninvasive tests (calprotectin, transferrin, hemoglobin) in complex diagnosis of diseases of intestines	N=52<br>Setting: unclear<br>Mean age (range): 38.6 years (18-49)<br>Female: unclear	Anxiety symptoms, weight loss, consistent abdominal pain, fever, pathology in abdominal organs, anaemia, increased red blood cell sedimentation rate, abdominal surgery in the past 6 months prior to enrolment, NSAIDs, antibacterial agents within one month of enrolment	Abdominal pain or discomfort, changes in stool frequency over the last 3 months	Organic versus non-organic disease (IBS)	Organic disease: 36/52 (69%)
Mowat 2016 [24]; UK	To study the diagnostic accuracies of quantitative FHb and FC tests in patients presenting to primary care with bowel symptoms	N=1031<br>Setting: referred patients from primary care<br>Median age (range): 64 years (16–90)<br>Female: 564/1031 (55%)	Faecal sample not suitable for analysis,<br>returned samples outside the study period,<br>known IBD, no colonoscopy, OGD only	Rectal bleeding, anaemia, diarrhoea, altered bowel habit, abdominal pain and weight loss	IBD versus non-IBD, Organic versus non-organic disease	IBD: 34/1031 (3.3%)
Otten 2008 [25]; Netherlands	To evaluate the diagnostic accuracy of two new rapid calprotectin and lactoferrin fecal tests in assessing colonic inflammation in patients with chronic abdominal complaints.	N=139<br>Setting: patients referred for endoscopy or sigmoidoscopy by the GP (80%) or the gastroenterologist (20%)<br>Mean age: IBS: 52.3 years<br>IBD: 44.5 years<br>Female: 61/114 (54%)	<18 years, patients with a history of colonic surgery and those with iron deficiency	Bloating, pain, change in defecation frequency or consistency, or blood and mucus in stool	IBD versus IBS	IBD 23/139 (17%)
Oyaert 2014 [26]; Belgium	To compare two FC assays regarding their reliability in the use of diagnosis of IBD	N=183<br>Setting: out- and inpatients with suspicion of IBD and FC<br>Median age (range): 32 years (14-89)<br>Female: 104/183 (57%)	<14 years, previous IBD diagnosis, no colonoscopy, unclear diagnosis (indeterminate colitis), inability to collect enough faecal samples	Diarrhoea, mucous, bloody stools, weight loss, abdominal pain and cramping	IBD versus non-IBD	IBD: 51/183 (27.9%)
Oyaert 2017 [27]; Belgium	To evaluate six different automated faecal calprotectin immunoassays for the diagnosis of IBD.	N=86<br>Setting: Patients in secondary care with suspicion of IBD and FC<br>Median age (range): 35 years (14-94)<br>Female: N=61/105 (58%)	<14 years, previous IBD diagnosis, no ileocolonoscopy, unclear diagnosis (e.g. indeterminate colitis), inability to collect enough faecal sample	Diarrhoea, mucous or bloody stools, weight loss, and abdominal pain and cramping	IBD versus non-IBD	IBD: 21/86 (24%)
Pavlidis 2013 [28]; UK	To assess the diagnostic performance of FC in routine general and the impact of different cut-offs	N=962<br>Setting: Suspected IBS patients in primary care<br>Mean age ± SD: 33 ±7 years	<18 and >45 years, previous diagnosis of IBD, missing clinical data, diarrhoea for <2 weeks	Persistent abdominal pain, bloating, alteration in stool form or frequency, passage of mucus	Organic versus non-organic disease	Organic disease: 94/962 (9.8%)

Study ID (author, year, country)	Aim	Population (N, setting, age, gender)	Study exclusions	Indication*	Clinical questions	Disease prevalence
	on endoscopy and referral rates.	Female: 577/962 (60%)				
Rosenfeld 2016 [29]; Canada	To evaluate the impact of FC test results for the differentiation of GI symptoms as IBS or IBD.	N=279<br>Setting: patients for whom gastroenterologist considered FC to be an appropriate next step in diagnosis and/or management of symptoms<br>Mean age ± SD (range): 39.4 ± 13.5 years (19-79)<br>Female: NR	ischemic colitis, infectious enterocolitis or colorectal cancer, pregnancy, history of extensive bowel resection, ostomy, ileoanal pouch, NSAIDS, inability to collect a stool sample	Gastrointestinal symptoms	IBD versus IBS	IBD: 4/50 (8%)
Schoepfer 2008 [30]; Switzerland	To determine the accuracy of 4 faecal markers in a patient group admitted for workup of GI symptoms.	N=136<br>Setting: Outpatients and inpatients admitted to colonoscopy ± upper endoscopy<br>Mean age (range): CD: 41 years (20–78)<br>UC: 45 years (23–72)<br>IBS: 40 years (20–79)<br>Female: 54/94 (57%)	Incomplete ileocolonoscopy, microscopic colitis, infectious ileocolitis, colorectal cancer, colorectal polyps, unclear diagnosis (e.g., indeterminate colitis), inability to collect fecal samples, history of colorectal or small bowel surgery, regular intake of aspirin and/or an NSAID (≥2 tablets/week).	Abdominal pain, altered bowel habit, and/or anorectal bleeding	IBD versus IBS	IBD: 64/94 (68%)
Schroeder 2007 [31]; Germany	To evaluate the clinical utility of FC to detect active gastrointestinal inflammation in patients suggestive of either having IBD or IBS.	N=76<br>Setting: patients referred for diagnostic clarification of chronic diarrhoea to tertiary referral practices<br>Median age (range):<br>CD: 40 (25–59) years<br>UC: 38 (24–75) years<br>IBS: 43 (20–72) years<br>Female: 43/76 (57%)	Previous evaluation for chronic diarrhoea, rectal bleeding, sigmoidoscopy or colonoscopy during previous 2 months, colorectal cancer syndrome and pregnancy	Chronic diarrhoea	IBD versus IBS	IBD: 45/76 (59%)
Sharbatdaran 2018[32] Iran	To evaluate the level of fecal calprotectin in patients with IBD and patients without inflammatory diseases of the colon	N=90<br>Setting: Referred to secondary care Mean age ± SD: 34.69±10.42 years<br>Female: 51/90 (56.7%)	Chronic disease and illness that caused fever, Patients with calprotectin level of 50-200µg	Clinical symptoms of colon diseases	IBD versus non-IBD	IBD: 45/90 (50%)
Shitrit 2007 [33]; Israel	To assess the predictive value of faecal calprotectin in organic colonic disease.	N=72<br>Setting: patients referred to gastroenterology for colonoscopy<br>Mean age ± SD: 58 ± 20 years<br>Female: 35/72 (48%)	NSAIDs, concomitant serious illness, pregnancy, alcohol abuse, respiratory tract infection	NR	Organic versus non-organic disease	Organic disease: 28/72 (39%)
Sostres 2017[34]; Spain	To evaluate the diagnostic accuracy of FC in	N=171	NR	Rectal bleeding, change of bowel habits, anaemia	Organic versus non-organic disease	Organic disease: 37/171 (21.6%)

Study ID (author, year, country)	Aim	Population (N, setting, age, gender)	Study exclusions	Indication*	Clinical questions	Disease prevalence
Abstract	symptomatic patients referred for diagnostic colonoscopy.	Setting: Referred for diagnostic colonoscopy Median age (IQR): 62 (51-68) years<br>Female: 73/171 (42.7)				
Tan 2016[35]; UK<br><br>Abstract	To evaluate the FC diagnostic referral pathway for primary care practitioners.	N=731<br>Setting: FC tests performed in primary care Median age: 40 years<br>Female: 461/731 (63%)	NR	NR	IBD versus non-IBD	IBD: 15/731 (2.1%)
Tibble 2002 [36]; UK	To determine if the use of FC is useful in differentiating between patients with organic and nonorganic disease.	N=602<br>Setting: patients referred to gastroenterology outpatient department by general practitioners<br>Median age (range): 40 years (18–90)<br>Female: 371/602 (62%)	Symptoms of oesophageal reflux, gastroesophageal pathology, or functional or isolated dyspepsia, known IBD, colorectal carcinoma, and serious cardiopulmonary, hepatic, renal, neurologic, and psychiatric disease	Abdominal pain, diarrhoea, constipation, anaemia, bleeding, weight loss	Organic disease versus other disease	Organic disease: 263/602 (44%)
Turvill 2012 [37]; UK	To determine the NPV of a normal faecal calprotectin in excluding organic intestinal disease in patients with intestinal symptoms referred unselectively from primary care.	N=630<br>Setting: patients with intestinal symptoms referred from primary care to department of gastroenterology<br>Mean age: Normal FC: 40.5 years<br>Raised FC: 41 years<br>Female: 442/630 (70%)	Patients with fast track colorectal symptoms	Pain/discomfort, diarrhoea, constipation, bloating, bleeding	Organic versus non-organic disease	Organic disease: 109/630 (17%)
Turvill 2016 [38]; UK	To evaluate the care pathway and recruit five primary care practices to conduct an FC implementation assessment.	N=262<br>Setting: patients presenting to primary care with new lower gastrointestinal symptoms<br>Mean age ± SD: 36.8±10.9 years<br>Female: 183/262 (70%)	Patients in whom the GP suspected cancer	Diarrhoea,<br>alternating bowel habit, pain, bloating, constipation	Organic versus non-organic disease	Organic disease: 26/262 (9.9%)

Table of study characteristics – investigations

Study ID (author, year, country)	FC test and cut-off	Reference standard	Proportion without ref standard excluded	Proportion of analysis sample with reference standard	Definition target condition	Definition non-target condition
Alrubaiy 2014 [1]; UK<br><br>Abstract	NR<br>50µg/g	Colonoscopy +/- capsule endoscopy or follow up at next clinic visit	None	28/54 (52%) colonoscopy +/- capsule endoscopy<br>26/54 (48%) follow up at next clinic visit	IBD	No Bowel inflammation
Banerjee 2015 [2]; UK	IDK ELISA<br>8 µg/g<br>25µg/g	Colonoscopy with histology	22/219 excluded from study	100%	IBD (excluding microscopic colitis)	Normal colonoscopy and histology

	50μg/g<br>75μg/g<br>100μg/g<br>150μg/g					
Bharathi 2005 [3];<br>UK<br><br>Abstract	PhiCal ELISA<br>60μg/g	OGD, flexible sigmoidoscopy, colonoscopy,<br>ultrasound, small bowel studies or clinical grounds	None	38/58 imaging<br>4/58 clinical grounds<br>16/58 unclear	IBD	IBS, non-intestinal, NSAIDS use, diverticulosis
Boyd 2016 [4];<br>UK<br><br>Abstract	NR<br>50μg/g<br>150μg/g	Investigation for referred not reported or median of 12 months (range 1-37) follow-up	None	Referred patients: NR<br><br>Non-referred patients: follow up	IBD (excluding microscopic colitis)	Gastroenteritis, microscopic colitis and IBS
Burri 2013 [5];<br>Switzerland	EK-CAL ELISA and PhiCal ELISA<br>50μg/g	Colonoscopy and histology +/- upper OGD	None recorded	70/405 with additional OGD	Organic: Esophagitis, erosive gastritis, gastric ulcer, gastric carcinoma, colitis (infectious colitis, CD, UC, diverticulitis, microscopic colitis), adenomatous polyp, CRC	Normal colonoscopy, hyperplastic polyp
Carroccio 2003 [6];<br>Italy	Calprest ELISA<br>50 μg/g<br>100 μg/g	Sigmoidoscopy or colonoscopy both with biopsy	None recorded	Sigmoidoscopy with biopsy if <40 years of age or colonoscopy with biopsy if >40	IBD (including microscopic colitis)	Colonoscopy / sigmoidoscopy negative and Manning criteria positive
Caviglia 2014 [7];<br>Italy	Calprest ELISA<br>50μg/g<br>100μg/g<br>150μg/g	Colonoscopy with biopsies	None reported	100%	IBD (including indeterminate colitis)	IBS
Conroy 2017 [8];<br>UK	IDK ELISA<br>50 μg/g<br>100 μg/g<br>125 μg/g<br>150 μg/g<br>250 μg/g	Colonoscopy, sigmoidoscopy, radiological features or small bowel video capsule endoscopy	None reported	146/410 (36%) colonoscopy (n=133) or sigmoidoscopy (n=13), 104/410 (25%) other investigation, 160 (39%) no investigation	IBD (including collagenous colitis, lymphocytic colitis)	Non-IBD (no further investigation and non-IBD organic conditions: CRC, colorectal adenomatous polyps, diverticulitis, appendicitis and diversion proctitis)
Damms 2008 [9];<br>Germany	EK-CAL ELISA<br>50 μg/g<br>Prevista qualitative rapid	Colonoscopy with histology	None recorded	100%	IBD (UC and CD)	normal diagnostic findings
De Sloovere 2017 [10]<br>Belgium	EK-CAL ELISA<br>50μg/g<br>163μg/g<br>325μg/g<br><br>fCal Turbo<br>50μg/g<br>81μg/g	Ileocolonoscopy with histopathology or 'sum of all findings'	None	136/229 (59%), rest unclear	IBD (excluding microscopic colitis)	IBS

	86µg/g<br>280µg/g<br><br>Diasorin CLIA<br>43µg/g<br>50µg/g<br>135µg/g					
Dhaliwal 2015 [11];<br>UK	PhiCal ELISA<br>50 and 100µg/g	Endoscopy/histology and/or radiology	None recorded	NR	IBD excluding microscopic colitis	IBS fulfilling the Rome II criteria
El-Badry 2010 [12];<br>Egypt	PhiCal ELISA<br>50 and 100 µg/g	Colonoscopy with biopsies	None recorded	100%	IBD	IBS following Rome II criteria
Garcia 2006 [13];<br>Spain	Calprest<br>217mg/kg	Colonoscopy and histology	None recorded	100%	Organic disease: colon adenomas, CRC, IBD	Other: IBS, diverticulosis, NSAIDs or aspirin, PPI
Hogberg 2017 [14];<br>Sweden	Calpro ELISA<br>20µg/g<br>50µg/g<br>100µg/g	Bowel imaging (colonoscopy, CT colonography, double contrast barium enema) or 2-year follow-up	None	185/384 (48%) imaging, 199/384 (52%) 2-year follow-up	IBD including microscopic colitis and unspecific colitis	No or any other pathology (including CRC, HRA)
Jang 2016 [15];<br>Korea	Ridascreen ELISA<br>Quantum-Blue rapid<br>EliA FEIA<br>50µg/g	Colonoscopy	None recorded	100%	IBD including intestinal Behçet's disease	IBS
Kennedy 2015 [16];<br>UK	PhiCal ELISA<br>20 µg/g<br>50 µg/g<br>70 µg/g<br>100 µg/g	Definitive organic diagnosis, full colonoscopy or minimum of 3 years follow up	None	467/895 (52%) organic diagnosis or colonoscopy, 428/895 (48%) follow up	IBD	Functional
Kok 2012 [17];<br>Netherlands	EK-CAL ELISA<br>Quantum-Blue rapid,<br>50 µg/g	Colonoscopy, sigmoidoscopy (89.9% with biopsy), other bowel examinations, 3 month follow-up	4/423<br>from study	Colonoscopy 351/423 (91.9%), sigmoidoscopy 21/423 (5.5%), other bowel examinations 10/423 (2.6%)<br>With histology in 89.9%<br>Follow-up for inconclusive tests	Organic disease: CRC, adenomas, IBD, and diverticulitis	Other: no structural abnormalities, diverticulosis, IBS, and haemorrhoids
Labaere 2014 [18];<br>Belgium	CalproLab ELISA 50µg/g<br>Quantum-Blue rapid 50µg/g and 75µg/g<br>Calfast rapid 70µg/g<br>Calprest ELISA 50µg/g<br>EliA FEIA 15µg/g and 50µg/g<br>CerTest rapid 50µg/g	Ileocolonoscopies + biopsy	1/33excluded from study	100%	IBD	Non-organic conditions + non-IBD organic conditions (nonsteroidal anti-inflammatory drug enteropathy, adenoma, and infectious colitis)
Lee 2014 [19];<br>UK	ELISA<br>60µg/g	Investigation in Gastroenterology clinic	None recorded	Patients with positive FC test	Organic disease: IBD, Diverticulosis, Colonic	Functional

Abstract					Polyps, Infective colitis and Chronic Pancreatitis	
Li 2006 [20]; China	PhiCal ELISA 50µg/g	Colonoscopy ± biopsy	None recorded	Patients with inflammation, unclear for patients with IBS	Inflammation: IBD, chronic infectious diarrhoea, intestinal tuberculosis, Behcet disease	IBS
Licata 2012 [21]; Italy	Calprest ELISA 150 µg/g	Colonoscopy with biopsy	None recorded	100%	Inflammation: IBD, microscopic colitis, diverticulitis, polyps, ischemic colitis, nonspecific colitis, IBS or undiagnosed microscopic colitis	No inflammation: IBS, diverticulosis
Limburg 2000 [22]; USA	PhiCal ELISA 100µg/g	Colonoscopy and histology	None recorded	100% (n=32 had no biopsy)	Inflammation: CD, UC, microscopic or collagenous colitis, peridiverticulitis, and eosinophilic colitis	normal mucosa, <1cm polyps
Lizvan 2015 [23]; Russia	Quantum-Blue rapid 50µg/g	Colonoscopy	None recorded	100%	Organic disease	IBS
Mowat 2016 [24]; UK	EK-CAL ELISA 50µg/g 200µg/g	Lower endoscopy	163/1031 from analysis	100%	IBD	all non-IBD conditions and normal
Otten 2008 [25]; Netherlands	PhiCal ELISA 50µg/g CalDetect rapid 15µg/g 60µg/g	Colonoscopy or sigmoidoscopy biopsies were taken if necessary	2/144 from study	109 (96%) colonoscopy, and 5 (4%) sigmoidoscopy	IBD	IBS
Oyaert 2014 [26]; Belgium	Quantum-Blue rapid, ELiA ELISA 50µg/g	Ileocolonoscopy and histology	From study, proportion not reported	100%	IBD (excluding indeterminate colitis and microscopic colitis)	IBS, infectious colitis, oesophagitis, erosive gastritis, gastric ulcers, diverticulitis, microscopic colitis, CRC, hyperplastic polyps, adenomatous polyps, coeliac disease, arthritis
Oyaert 2017 [27]; Belgium	EliA ELISA Diasorin CLIA Quanta Flash CLIA fCAL Turbo rapid Euroimmun ELISA Orgentec Alegria ELISA 50 µg/g and test specific optimal cut-off	Ileocolonoscopy and histology	From study, proportion not reported	100%	IBD (excluding microscopic colitis)	oesophagitis, erosive gastritis, gastric ulcers, diverticulitis, microscopic colitis, colorectal cancer, hyperplastic polyps, adenomatous polyps, spondylo-arthritis, (undifferentiated) arthritis, IBS

Pavlidis 2013 [28]; UK	EK-CAL ELISA 50µg/g 100 µg/g 125 µg/g 150 µg/g 200 µg/g 249 µg/g	Colonoscopy, sigmoidoscopy, other investigations or 1 year follow-up	None	134/962 (14%) colonoscopy, 104/962 (11%) sigmoidoscopy, 130/962 (14%) other, 594/962 (62%) follow-up	Organic disease: IBD, Microscopic colitis, diversion colitis, infective diarrhoea, coeliac, NSAID, Alcohol related, Meckel's diverticulum, Rectal adenocarcinoma	Non-organic
Rosenfeld 2016 [29]; Canada	Quantum-Blue rapid, 100µg/g	Endoscopy, 26-months follow-up	None	8/41 FC negatives and 4/9 FC positives had endoscopy, remainder had 26 months follow up	IBD including microscopic colitis	IBS
Schoepfer 2008 [30]; Switzerland	PhiCal ELISA 50µg/g	Colonoscopy with biopsy	5/187 from study	100%	IBD	IBS according to Rome II criteria
Schroeder 2007 [31]; Germany	IDK ELISA 15µg/ g	Colonoscopy with biopsy	None recorded	100%	IBD	IBS according to Rome II criteria
Sharbatdaran 2018[32] Iran	EK-CAL ELISA 127.65µg/g	Colonoscopy with biopsy	None	100%	IBD	Non-IBD inflammatory bowel diseases, IBS, no diagnosed disease
Shitrit 2007 [33]; Israel	Calprest ELISA 150µg/g	Colonoscopy	None recorded	100%	Organic: IBD, carcinoma, polyps	Normal colonoscopy
Sostres 2017[34]; Spain<br><br>Abstract	EliA FEIA 50µg/g	Colonic investigations (colonoscopy)	None	100%	CRC, advanced adenoma, IBD and angiodysplasia	NR
Tan 2016[35]; UK<br><br>Abstract	NR 50µg/g	Colonoscopies, gastroscopies, MRI of small bowel	None	NR for FC positive FC negatives: 58/95colonoscopy 19/95 gastroscopy, 3/95 MRI of small bowel	IBD	NR
Tibble 2002 [36]; UK	ELISA 10mg/L=50µg/g	Barium enteroclysis/ enema and/or endoscopy/colonoscopy	None recorded	372/602 patients had a full colonoscopy	Crohn's disease, Celiac disease, Infective diarrhea, small bowel enteropathy (NSAID, Alcoholic, Radiation), Diabetic diarrhea, ulcerative colitis, Microscopic colitis, Collagenous colitis, Diverticular disease, cancer	IBS, IBS + nonulcer dyspepsia, IBS + other (lactose intolerance, angiodysplasia, hemorrhoids, and melanosis coli)
Turvill 2012 [37]; UK	PhiCal ELISA 50µg/g	Colonoscopy or barium enema ± histology, or various other investigations	None recorded	FC negative: 43% full evaluation of the colon by colonoscopy or barium enema and 60% had histology.	Organic disease: Crohn's disease, Ulcerative colitis, Indeterminate IBD, Microscopic colitis, NSAID enteropathy, Gastroenteritis, Diverticular	Non-organic: IBS

				FC positive: 75% underwent full colonic evaluation by colonoscopy or barium enema, supportive histology in 83%	disease, Bacterial overgrowth, Coeliac disease, Postoperative, Upper gastrointestinal bleed, Intussusception, Rectal polyp, Alcoholic enteropathy, Solitary rectal ulcer, Gastrinoma, Cholecystitis, Appendicitis, Bile salt malabsorption, Giardiasis, Chronic pancreatitis, Thyrotoxicosis, Small bowel bacterial overgrowth, Lactose intolerance, Sorbitol induced diarrhoea	
Turvill 2016 [38]; UK	EK-CAL ELISA 100µg/g 250µg/g	Colonoscopy, 6-week review of symptoms by GP or 6 months follow-up, hospital database	None	25% received colonoscopy, remainder received 6-week review of symptoms by GP or follow-up	Organic disease: IBD, non-specific inflammation, microscopic colitis, diverticular disease, gastroenteritis, coeliac disease, pancreatic insufficiency and a low-grade tubulovillous adenoma, haemorrhoids	IBS (incorporated all functional intestinal disease diagnoses) and non-enteric other diagnosis following a normal FC

References

1. Alrubaiy L, Malik A, Rees I, Bowen D. Usefulness of fecal calprotectin in clinical practice in a district general hospital. *Inflammatory Bowel Diseases*. 2012;18:S53-S4.
2. Banerjee A, Srinivas M, Eyre R, Ellis R, Waugh N, Bardhan KD, *et al.* Faecal calprotectin for differentiating between irritable bowel syndrome and inflammatory bowel disease: A useful screen in daily gastroenterology practice. *Frontline Gastroenterology*. 2015;6(1):20-6.
3. Bharathi S, Moncur P, Holbrook I, Kelly S. A normal facael calprotectin has a high negative predictive value in cases of suspected irritable bowel syndrome. *Gastroenterology*. 2005;128(4):A459.
4. Boyd A, Peplow C, Dhaliwal A, O'Rourke J, Davies J, Milestone A. Primary care faecal calprotectin pathway - A three year analysis. *Colorectal Disease*. 2016;18:27.
5. Burri E, Manz M, Rothen C, Rossi L, Beglinger C, Lehmann FS. Monoclonal antibody testing for fecal calprotectin is superior to polyclonal testing of fecal calprotectin and lactoferrin to identify organic intestinal disease in patients with abdominal discomfort. *Clin Chim Acta*. 2013;416:41-7.
6. Carroccio A, Iacono G, Cottone M, Di Prima L, Cartabellotta F, Cavataio F, *et al.* Diagnostic accuracy of fecal calprotectin assay in distinguishing organic causes of chronic diarrhea from irritable bowel syndrome: a prospective study in adults and children. *Clin Chem*. 2003;49(6 Pt 1):861-7.
7. Caviglia GP, Pantaleoni S, Touscoz GA, Adriani A, Rosso C, Smedile A, *et al.* Fecal calprotectin is an effective diagnostic tool that differentiates inflammatory from functional intestinal disorders. *Scandinavian Journal of Gastroenterology*. 2014;49(12):1419-24.
8. Conroy S, Hale MF, Cross SS, Swallow K, Sidhu RH, Sargur R, *et al.* Unrestricted faecal calprotectin testing performs poorly in the diagnosis of inflammatory bowel disease in patients in primary care. *J Clin Pathol*. 2017.

9. Damms A, Bischoff SC. Validation and clinical significance of a new calprotectin rapid test for the diagnosis of gastrointestinal diseases. *Int J Colorectal Dis*. 2008;23(10):985-92.
10. De Sloovere MM, De Smet D, Baert FJ, Debrabandere J, Vanpoucke HJ. Analytical and diagnostic performance of two automated fecal calprotectin immunoassays for detection of inflammatory bowel disease. *Clinical Chemistry & Laboratory Medicine*. 2017;11:11.
11. Dhaliwal A, Zeino Z, Tomkins C, Cheung M, Nwokolo C, Smith S, *et al.* Utility of faecal calprotectin in inflammatory bowel disease (IBD): what cut-offs should we apply? *Frontline Gastroenterology*. 2015;6(1):14-9.
12. El-Badry A, Sedrak H, Rashed L. Faecal calprotectin in differentiating between functional and organic bowel diseases. *Arab Journal of Gastroenterology*. 2010;11(2):70-3.
13. Garcia Sanchez Mdel V, Gonzalez R, Iglesias Flores E, Gomez Camacho F, Casais Juanena L, Cerezo Ruiz A, *et al.* [Diagnostic value of fecal calprotectin in predicting an abnormal colonoscopy]. *Med Clin (Barc)*. 2006;127(2):41-6. Precision diagnostica de la calprotectina fecal para predecir una colonoscopia patologica.
14. Hogberg C, Karling P, Rutegard J, Lilja M. Diagnosing colorectal cancer and inflammatory bowel disease in primary care: The usefulness of tests for faecal haemoglobin, faecal calprotectin, anaemia and iron deficiency. A prospective study. *Scandinavian Journal of Gastroenterology*. 2017;52(1):69-75.
15. Jang HW, Kim HS, Park SJ, Hong SP, Kim TI, Kim WH, *et al.* Accuracy of three different fecal calprotectin tests in the diagnosis of inflammatory bowel disease. *Intestinal Research*. 2016;14(4):305-13.
16. Kennedy NA, Clark A, Walkden A, Chang JC, Fasci-Spurio F, Muscat M, *et al.* Clinical utility and diagnostic accuracy of faecal calprotectin for IBD at first presentation to gastroenterology services in adults aged 16-50 years. *Journal of Crohn's & colitis*. 2015;9(1):41-9.
17. Kok L, Elias SG, Witteman BJ, Goedhard JG, Muris JW, Moons KG, *et al.* Diagnostic accuracy of point-of-care fecal calprotectin and immunochemical occult blood tests for diagnosis of organic bowel disease in primary care: the Cost-Effectiveness of a Decision Rule for Abdominal Complaints in Primary Care (CEDAR) study. *Clinical Chemistry*. 2012;58(6):989-98.
18. Labaere D, Smismans A, Van Olmen A, Christiaens P, D'Haens G, Moons V, *et al.* Comparison of six different calprotectin assays for the assessment of inflammatory bowel disease. *United European Gastroenterology Journal*. 2014;2(1):30-7.
19. Lee S, Borthwick H, Dhar A. P223. Faecal calprotectin testing in primary and secondary care - Are the current manufacturer's cut-off values clinically useful? 9th Congress of ECCO 2014; Copenhagen: European Crohn's and Colitis Organisation; 2014.
20. Li XG, Lu YM, Gu F, Yang XL. [Fecal calprotectin in differential diagnosis of irritable bowel syndrome]. *Beijing Da Xue Xue Bao*. 2006;38(3):310-3.
21. Licata A, Randazzo C, Cappello M, Calvaruso V, Butera G, Florena AM, *et al.* Fecal calprotectin in clinical practice: a noninvasive screening tool for patients with chronic diarrhea. *J Clin Gastroenterol*. 2012;46(6):504-8.
22. Limburg PJ, Ahlquist DA, Sandborn WJ, Mahoney DW, Devens ME, Harrington JJ, *et al.* Fecal calprotectin levels predict colorectal inflammation among patients with chronic diarrhea referred for colonoscopy. *Am J Gastroenterol*. 2000;95(10):2831-7.
23. Livzan MA, Lyalukova EA, Nechaeva G, Osipenko MF, Dolgih TI. [Fecal Noninvasive Tests (Calprotectin, Transferrin, Hemoglobin) in Complex Diagnosis of Diseases of Intestines]. *Eksperimental'Naia i Klinicheskaia Gastroenterologiia*. 2015(3):34-8.
24. Mowat C, Digby J, Strachan JA, Wilson R, Carey FA, Fraser CG, *et al.* Faecal haemoglobin and faecal calprotectin as indicators of bowel disease in patients presenting to primary care with bowel symptoms. *Gut*. 2016;65(9):1463-9.
25. Otten CM, Kok L, Witteman BJ, Baumgarten R, Kampman E, Moons KG, *et al.* Diagnostic performance of rapid tests for detection of fecal calprotectin and lactoferrin and their ability to discriminate inflammatory from irritable bowel syndrome. *Clin Chem Lab Med*. 2008;46(9):1275-80.
26. Oyaert M, Trouve C, Baert F, De Smet D, Langlois M, Vanpoucke H. Comparison of two immunoassays for measurement of faecal calprotectin in detection of inflammatory bowel disease: (pre)-analytical and diagnostic performance characteristics. *Clinical Chemistry & Laboratory Medicine*. 2014;52(3):391-7.
27. Oyaert M, Boel A, Jacobs J, Van den Bremt S, De Sloovere M, Vanpoucke H, *et al.* Analytical performance and diagnostic accuracy of six different faecal calprotectin assays in inflammatory bowel disease. *Clinical Chemistry & Laboratory Medicine*. 2017;21:21.

28. Pavlidis P, Chedgy FJ, Tibble JA. Diagnostic accuracy and clinical application of faecal calprotectin in adult patients presenting with gastrointestinal symptoms in primary care. *Scandinavian Journal of Gastroenterology*. 2013;48(9):1048-54.
29. Rosenfeld G, Greenup AJ, Round A, Takach O, Halparin L, Saadeddin A, *et al.* FOCUS: Future of fecal calprotectin utility study in inflammatory bowel disease. *World Journal of Gastroenterology*. 2016;22(36):8211-8.
30. Schoepfer AM, Trummler M, Seeholzer P, Seibold-Schmid B, Seibold F. Discriminating IBD from IBS: comparison of the test performance of fecal markers, blood leukocytes, CRP, and IBD antibodies. *Inflamm Bowel Dis*. 2008;14(1):32-9.
31. Schroder O, Naumann M, Shastri Y, Povse N, Stein J. Prospective evaluation of faecal neutrophil-derived proteins in identifying intestinal inflammation: combination of parameters does not improve diagnostic accuracy of calprotectin. *Aliment Pharmacol Ther*. 2007;26(7):1035-42.
32. Sharbatdaran M, Halakou A, Kashifard M, Bijani A, Firozjaei A, Hossaini A, *et al.* Fecal calprotectin Level in patients with IBD and noninflammatory disease of colon: a study in Babol, Northern, Iran. *Caspian Journal of Internal Medicine*. 2018;9(1):60-4.
33. Shitrit AB, Braverman D, Stankiewics H, Shitrit D, Peled N, Paz K. Fecal calprotectin as a predictor of abnormal colonic histology. *Dis Colon Rectum*. 2007;50(12):2188-93.
34. Sostres C, Lue A, Barra Pardos MV, Hijos G, Perales A, Puente JJ, *et al.* Combination of FOBT and fecal calprotectin may be useful for reducing unnecessary colonoscopies in symptomatic patients. *United European Gastroenterology Journal*. 2017;5 (5 Supplement 1):A779.
35. Tan M, Subramaniam Y, Cheong C, Hasan F, Moran GW, Fateen W, *et al.* The role of faecal calprotectin in a primary care referral pathway in the UK. *United European Gastroenterology Journal*. 2016;4 (5 Supplement 1):A443.
36. Tibble JA, Sigthorsson G, Foster R, Forgacs I, Bjarnason I. Use of surrogate markers of inflammation and Rome criteria to distinguish organic from nonorganic intestinal disease. *Gastroenterology*. 2002;123(2):450-60.
37. Turvill J. High negative predictive value of a normal faecal calprotectin in patients with symptomatic intestinal disease. *Frontline Gastroenterology*. 2012;3(1):21-8.
38. Turvill J, O'Connell S, Brooks A, Bradley-Wood K, Laing J, Thiagarajan S, *et al.* Evaluation of a faecal calprotectin care pathway for use in primary care. *Primary Health Care Research & Development*. 2016;17(5):428-36.

Supplement 6

Table of risk of bias and applicability concern for each included study

Study	RISK OF BIAS				APPLICABILITY CONCERNS		
	PATIENT SELECTION	INDEX TEST	REFERENCE STANDARD	FLOW AND TIMING	PATIENT SELECTION	INDEX TEST	REFERENCE STANDARD
Alrubaiy 2012	😐	☺	😐	☹	😐	☺	😐
Banerjee 2015	☺	😐	☹	☹	☹	☺	☺
Bharathi 2005	😐	😐	😐	☹	😐	☺	😐
Boyd 2016	😐	☺	☹	☹	☺	☺	☹
Burri 2013	😐	☺	☹	☹	☹	☺	☹
Carroccio 2003	☺	☺	☹	☹	☹	☺	☹
Caviglia 2014	☹	☺	☺	😐	☹	☺	☺
Conroy 2017	☺	☺	☹	☹	😐	☺	☹
Damms 2008	😐	☺	☺	☹	☹	☺	☺
De Sloovere 2017	☹	☺	😐	☹	☹	☺	😐
Dhaliwal 2015	😐	😐	😐	☹	☹	☺	😐
El Badry 2010	😐	😐	😐	☺	☹	☺	☺
Garcia 2006	😐	☹	😐	☺	☹	☺	☹
Hogberg 2017	☺	☺	☹	☹	☹	☺	☺
Jang 2016	☹	☺	😐	☹	☹	☺	😐
Kennedy 2015	☺	😐	☹	☹	☹	☺	☹
Kok 2012	😐	☺	☺	☹	☹	☺	☹
Labaere 2014	😐	☹	☺	☺	☹	☺	☺
Lee 2014	😐	😐	😐	😐	☹	☺	☹
Li 2006	☹	😐	😐	😐	☹	☺	😐
Licata 2012	☺	☹	☺	😐	☹	☺	☺
Limburg 2000	😐	☺	☹	☹	☹	☺	☹
Lizvan 2015	😐	😐	😐	😐	😐	😐	☹
Mowat 2016	☹	☺	😐	☹	☹	☺	😐
Otten 2008	☹	☺	☹	☹	☹	☺	☹
Oyaert 2014	☹	😐	☺	😐	☹	☺	☺
Oyaert 2017	☹	☹	☺	😐	☹	☺	☺
Pavlidis 2013	😐	☺	☹	☹	☺	☺	☹
Rosenfeld 2016	😐	☺	☹	☹	😐	☺	😐
Schoepfer 2008	☹	☺	☺	☺	☹	☺	☺
Schroeder 2007	☺	☺	☺	☺	☹	☺	☺
Sharbatdaran 2018	☹	☹	😐	☹	☹	😐	☺
Shitrit 2007	😐	☹	😐	😐	☹	☺	☹
Sostres 2017	😐	😐	😐	😐	☹	😐	☹

Tan 2016	😐	☺	☹	☹	😐	☺	☹
Tibble 2002	😐	☹	☹	☹	☹	☺	☹
Turvill 2012	😐	😐	☹	☹	☹	☺	☹
Turvill 2016	☺	☺	☹	☹	☺	☺	☹

☹ high 😐 unclear ☺ low

Supplement 7 Paired forest plots of sensitivity and specificity of 38 included studies by clinical question (Letters following the year indicate different 2x2 table data from the same study reference)

Study	TP	FP	FN	TN	test type	Clin question	cut-off in µg/g	Setting	test name	Sensitivity (95% CI)	Specificity (95% CI)
Banerjee 2015 a	12	48	0	50	ELISA	IBD vs IBS	8.0	secondary	IDK	1.00 [0.74, 1.00]	0.51 [0.41, 0.61]
Otten 2008b	23	5	0	86	POCT	IBD vs IBS	15.0	secondary	CalDetect	1.00 [0.85, 1.00]	0.95 [0.88, 0.98]
Schroeder 2007	42	0	3	31	ELISA	IBD vs IBS	15.0	secondary	IDK	0.93 [0.82, 0.99]	1.00 [0.89, 1.00]
Kennedy 2015a	90	289	1	277	ELISA	IBD vs functional	20.0	secondary	PhiCal	0.99 [0.94, 1.00]	0.49 [0.45, 0.53]
Banerjee 2015 b	12	46	0	52	ELISA	IBD vs IBS	25.0	secondary	IDK	1.00 [0.74, 1.00]	0.53 [0.43, 0.63]
De Sloovere 2017i	45	15	1	101	CLIA	IBD vs IBS	43.0	secondary	Diasorin	0.98 [0.88, 1.00]	0.87 [0.80, 0.93]
Carroccio 2003a	11	8	0	32	ELISA	IBD vs IBS	50.0	secondary	Calprest	1.00 [0.72, 1.00]	0.80 [0.64, 0.91]
Caviglia 2014 a	24	10	0	11	ELISA	IBD vs IBS	50.0	secondary	Calprest	1.00 [0.86, 1.00]	0.52 [0.30, 0.74]
De Sloovere 2017h	43	14	3	102	CLIA	IBD vs IBS	50.0	secondary	Diasorin	0.93 [0.82, 0.99]	0.88 [0.81, 0.93]
De Sloovere 2017a	46	55	0	61	ELISA	IBD vs IBS	50.0	secondary	EK-CAL	1.00 [0.92, 1.00]	0.53 [0.43, 0.62]
Damms 2008a	18	12	0	44	ELISA	IBD vs functional	50.0	secondary	EK-CAL	1.00 [0.81, 1.00]	0.79 [0.66, 0.88]
Jang 2016a	30	1	1	4	FEIA	IBD vs IBS	50.0	secondary	EliA	0.97 [0.83, 1.00]	0.80 [0.28, 0.99]
Banerjee 2015 c	12	39	0	59	ELISA	IBD vs IBS	50.0	secondary	IDK	1.00 [0.74, 1.00]	0.60 [0.50, 0.70]
Dhaliwal 2015	126	33	18	115	ELISA	IBD vs IBS	50.0	secondary	PhiCal	0.88 [0.81, 0.92]	0.78 [0.70, 0.84]
El-Badry 2010a	9	3	0	17	ELISA	IBD vs IBS	50.0	secondary	PhiCal	1.00 [0.66, 1.00]	0.85 [0.62, 0.97]
Otten 2008a	22	12	1	79	ELISA	IBD vs IBS	50.0	secondary	PhiCal	0.96 [0.78, 1.00]	0.87 [0.78, 0.93]
Schoepfer 2008	53	0	11	30	ELISA	IBD vs IBS	50.0	secondary	PhiCal	0.83 [0.71, 0.91]	1.00 [0.88, 1.00]
Kennedy 2015b	88	149	3	417	ELISA	IBD vs functional	50.0	secondary	PhiCal	0.97 [0.91, 0.99]	0.74 [0.70, 0.77]
Damms 2008b	16	11	2	45	POCT	IBD vs functional	50.0	secondary	Prevista	0.89 [0.65, 0.99]	0.80 [0.68, 0.90]
Jang 2016b	31	1	0	4	POCT	IBD vs IBS	50.0	secondary	Quantum-Blue	1.00 [0.89, 1.00]	0.80 [0.28, 0.99]
Jang 2016c	25	0	6	5	ELISA	IBD vs IBS	50.0	secondary	Ridascreen	0.81 [0.63, 0.93]	1.00 [0.48, 1.00]
De Sloovere 2017d	46	30	0	86	PETIA	IBD vs IBS	50.0	secondary	fCal Turbo	1.00 [0.92, 1.00]	0.74 [0.65, 0.82]
Otten 2008c	14	2	9	89	POCT	IBD vs IBS	60.0	secondary	CalDetect	0.61 [0.39, 0.80]	0.98 [0.92, 1.00]
Bharathi 2005	0	12	0	46	ELISA	IBD vs IBS	60.0	secondary	PhiCal	Not estimable	0.79 [0.67, 0.89]
Kennedy 2015c	88	112	3	454	ELISA	IBD vs functional	70.0	secondary	PhiCal	0.97 [0.91, 0.99]	0.80 [0.77, 0.83]
Banerjee 2015 d	11	25	1	73	ELISA	IBD vs IBS	75.0	secondary	IDK	0.92 [0.62, 1.00]	0.74 [0.65, 0.83]
De Sloovere 2017e	46	16	0	100	PETIA	IBD vs IBS	81.0	secondary	fCal Turbo	1.00 [0.92, 1.00]	0.86 [0.79, 0.92]
De Sloovere 2017f	45	15	1	101	PETIA	IBD vs IBS	86.0	secondary	fCal Turbo	0.98 [0.88, 1.00]	0.87 [0.80, 0.93]
Caviglia 2014 b	21	6	3	15	ELISA	IBD vs IBS	100.0	secondary	Calprest	0.88 [0.68, 0.97]	0.71 [0.48, 0.89]
Banerjee 2015 e	11	18	1	80	ELISA	IBD vs IBS	100.0	secondary	IDK	0.92 [0.62, 1.00]	0.82 [0.73, 0.89]
El-Badry 2010b	7	0	2	20	ELISA	IBD vs IBS	100.0	secondary	PhiCal	0.78 [0.40, 0.97]	1.00 [0.83, 1.00]
Kennedy 2015d	87	74	4	492	ELISA	IBD vs functional	100.0	secondary	PhiCal	0.96 [0.89, 0.99]	0.87 [0.84, 0.90]
Rosenfeld 2016	4	5	0	41	POCT	IBD vs IBS	100.0	secondary	Quantum-Blue	1.00 [0.40, 1.00]	0.89 [0.76, 0.96]
De Sloovere 2017j	36	2	10	114	CLIA	IBD vs IBS	135.0	secondary	Diasorin	0.78 [0.64, 0.89]	0.98 [0.94, 1.00]
Caviglia 2014 c	21	2	3	19	ELISA	IBD vs IBS	150.0	secondary	Calprest	0.88 [0.68, 0.97]	0.90 [0.70, 0.99]
Banerjee 2015 f	10	14	2	84	ELISA	IBD vs IBS	150.0	secondary	IDK	0.83 [0.52, 0.98]	0.86 [0.77, 0.92]
De Sloovere 2017b	45	11	1	105	ELISA	IBD vs IBS	163.0	secondary	EK-CAL	0.98 [0.88, 1.00]	0.91 [0.84, 0.95]
De Sloovere 2017g	37	4	9	112	PETIA	IBD vs IBS	280.0	secondary	fCal Turbo	0.80 [0.66, 0.91]	0.97 [0.91, 0.99]
De Sloovere 2017c	38	4	8	112	ELISA	IBD vs IBS	325.0	secondary	EK-CAL	0.83 [0.69, 0.92]	0.97 [0.91, 0.99]

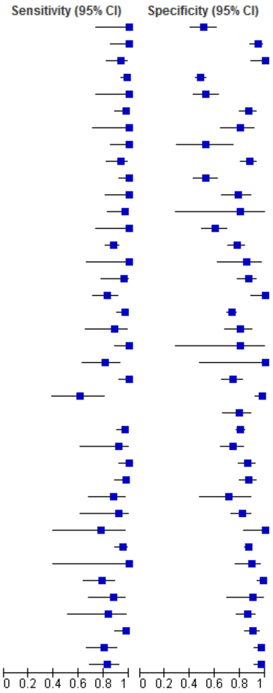

Forest plots of studies reporting data for clinical questions IBD vs IBS and IBD vs functional disease (14 studies)

Study	TP	FP	FN	TN	test type	Clin question	cut-off in µg/g	Setting	test name	Sensitivity (95% CI)	Specificity (95% CI)
Hogberg 2017 a	10	175	0	188	ELISA	IBD vs non-IBD	20.0	primary	Calpro	1.00 [0.69, 1.00]	0.52 [0.47, 0.57]
Conroy 2017a	8	140	3	259	ELISA	IBD vs non-IBD	50.0	primary	IDK	0.73 [0.39, 0.94]	0.65 [0.60, 0.70]
Boyd 2016 a	23	117	0	284	unclear	IBD vs non-IBD	50.0	primary	NR	1.00 [0.85, 1.00]	0.71 [0.66, 0.75]
Hogberg 2017 b	9	100	1	263	ELISA	IBD vs non-IBD	50.0	primary	Calpro	0.90 [0.55, 1.00]	0.72 [0.68, 0.77]
Conroy 2017b	6	78	5	321	ELISA	IBD vs non-IBD	100.0	primary	IDK	0.55 [0.23, 0.83]	0.80 [0.76, 0.84]
Hogberg 2017 c	7	51	3	312	ELISA	IBD vs non-IBD	100.0	primary	Calpro	0.70 [0.35, 0.93]	0.86 [0.82, 0.89]
Conroy 2017c	6	66	5	333	ELISA	IBD vs non-IBD	125.0	primary	IDK	0.55 [0.23, 0.83]	0.83 [0.79, 0.87]
Conroy 2017d	5	53	6	346	ELISA	IBD vs non-IBD	150.0	primary	IDK	0.45 [0.17, 0.77]	0.87 [0.83, 0.90]
Boyd 2016 b	21	44	2	357	unclear	IBD vs non-IBD	150.0	primary	NR	0.91 [0.72, 0.99]	0.89 [0.86, 0.92]
Conroy 2017e	3	26	8	373	ELISA	IBD vs non-IBD	250.0	primary	IDK	0.27 [0.06, 0.61]	0.93 [0.91, 0.96]
Labaere 2014a	10	3	2	16	FEIA	IBD vs non-IBD	15.0	secondary	EliA	0.83 [0.52, 0.98]	0.84 [0.60, 0.97]
De Sloovere 2017s	39	34	1	62	CLIA	IBD vs non-IBD	34.0	secondary	Diasorin	0.97 [0.87, 1.00]	0.65 [0.54, 0.74]
De Sloovere 2017k	40	54	0	42	ELISA	IBD vs non-IBD	50.0	secondary	EK-CAL	1.00 [0.91, 1.00]	0.44 [0.34, 0.54]
Carroccio 2003b	11	16	0	43	ELISA	IBD vs non-IBD	50.0	secondary	Calprest	1.00 [0.72, 1.00]	0.73 [0.60, 0.84]
Oyaert 2017i	21	27	0	38	ELISA	IBD vs non-IBD	50.0	secondary	Euroimmun	1.00 [0.84, 1.00]	0.58 [0.46, 0.71]
Oyaert 2017g	21	27	0	38	ELISA	IBD vs non-IBD	50.0	secondary	Orgentec	1.00 [0.84, 1.00]	0.58 [0.46, 0.71]
Oyaert 2017e	21	14	0	51	CLIA	IBD vs non-IBD	50.0	secondary	Diasorin	1.00 [0.84, 1.00]	0.78 [0.67, 0.88]
Oyaert 2017c	21	18	0	47	CLIA	IBD vs non-IBD	50.0	secondary	Quanta Flash	1.00 [0.84, 1.00]	0.72 [0.60, 0.83]
Oyaert 2017k	21	22	0	43	PETIA	IBD vs non-IBD	50.0	secondary	fCal Turbo	1.00 [0.84, 1.00]	0.66 [0.53, 0.77]
De Sloovere 2017o	40	45	0	51	PETIA	IBD vs non-IBD	50.0	secondary	fCal Turbo	1.00 [0.91, 1.00]	0.53 [0.43, 0.63]
De Sloovere 2017t	38	33	2	63	CLIA	IBD vs non-IBD	50.0	secondary	Diasorin	0.95 [0.83, 0.99]	0.66 [0.55, 0.75]
El-Badry 2010c	9	13	0	17	ELISA	IBD vs non-IBD	50.0	secondary	PhiCal	1.00 [0.66, 1.00]	0.57 [0.37, 0.75]
Labaere 2014e	10	6	2	13	POCT	IBD vs non-IBD	50.0	secondary	Quantum-Blue	0.83 [0.52, 0.98]	0.68 [0.43, 0.87]
Labaere 2014g	10	3	2	16	POCT	IBD vs non-IBD	50.0	secondary	CerTest	0.83 [0.52, 0.98]	0.84 [0.60, 0.97]
Labaere 2014b	9	1	3	18	FEIA	IBD vs non-IBD	50.0	secondary	EliA	0.75 [0.43, 0.95]	0.95 [0.74, 1.00]
Labaere 2014c	9	2	2	15	ELISA	IBD vs non-IBD	50.0	secondary	Calprest	0.82 [0.48, 0.98]	0.88 [0.64, 0.99]
Labaere 2014d	10	2	2	17	FEIA	IBD vs non-IBD	50.0	secondary	CalproLab	0.83 [0.52, 0.98]	0.89 [0.67, 0.99]
Oyaert 2014a	48	17	3	115	FEIA	IBD vs non-IBD	50.0	secondary	EliA	0.94 [0.84, 0.99]	0.87 [0.80, 0.92]
Tan 2016	15	165	0	95	unclear	IBD vs non-IBD	50.0	secondary	NR	1.00 [0.78, 1.00]	0.37 [0.31, 0.43]
Oyaert 2014b	51	37	0	95	POCT	IBD vs non-IBD	50.0	secondary	Quantum-Blue	1.00 [0.93, 1.00]	0.72 [0.63, 0.79]
Oyaert 2017a	21	22	0	43	FEIA	IBD vs non-IBD	50.0	secondary	EliA	1.00 [0.84, 1.00]	0.66 [0.53, 0.77]
Mowat 2016a	29	421	3	273	ELISA	IBD vs non-IBD	50.0	secondary	EK-CAL	0.91 [0.75, 0.98]	0.39 [0.36, 0.43]
Alrubaiy 2012	2	18	0	34	unclear	Infl vs non-infl	50.0	secondary	NR	1.00 [0.16, 1.00]	0.65 [0.51, 0.78]
Caviglia 2014d	38	10	7	11	ELISA	Infl vs non-infl	50.0	secondary	Calprest	0.84 [0.71, 0.94]	0.52 [0.30, 0.74]
Li 2006	55	6	5	54	ELISA	Infl vs non-infl	50.0	secondary	PhiCal	0.92 [0.82, 0.97]	0.90 [0.79, 0.96]
Labaere 2014h	9	2	2	15	POCT	IBD vs non-IBD	70.0	secondary	Calfast	0.82 [0.48, 0.98]	0.88 [0.64, 0.99]
Labaere 2014f	10	3	2	16	POCT	IBD vs non-IBD	75.0	secondary	Quantum-Blue	0.83 [0.52, 0.98]	0.84 [0.60, 0.97]
De Sloovere 2017u	36	20	4	76	CLIA	IBD vs non-IBD	78.0	secondary	Diasorin	0.90 [0.76, 0.97]	0.79 [0.70, 0.87]
De Sloovere 2017p	39	38	1	58	PETIA	IBD vs non-IBD	83.0	secondary	fCal Turbo	0.97 [0.87, 1.00]	0.60 [0.50, 0.70]
El-Badry 2010d	7	8	2	22	ELISA	IBD vs non-IBD	100.0	secondary	PhiCal	0.78 [0.40, 0.97]	0.73 [0.54, 0.88]
Caviglia 2014e	31	6	14	15	ELISA	Infl vs non-infl	100.0	secondary	Calprest	0.69 [0.53, 0.82]	0.71 [0.48, 0.89]
Limburg 2000	24	14	5	67	ELISA	Infl vs non-infl	100.0	secondary	PhiCal	0.83 [0.64, 0.94]	0.83 [0.73, 0.90]
Oyaert 2017f	21	2	0	63	CLIA	IBD vs non-IBD	111.0	secondary	Diasorin	1.00 [0.84, 1.00]	0.97 [0.89, 1.00]
Oyaert 2017d	21	9	0	56	CLIA	IBD vs non-IBD	115.0	secondary	Quanta Flash	1.00 [0.84, 1.00]	0.86 [0.75, 0.93]
Sharbatdaran 2018	33	3	12	40	ELISA	IBD vs non-IBD	127.65	secondary	EK-CAL	0.73 [0.58, 0.85]	0.93 [0.81, 0.99]
Caviglia 2014f	30	2	15	19	ELISA	Infl vs non-infl	150.0	secondary	Calprest	0.67 [0.51, 0.80]	0.90 [0.70, 0.99]
Licata 2012	107	24	35	180	ELISA	Infl vs non-infl	150.0	secondary	Calprest	0.75 [0.67, 0.82]	0.88 [0.83, 0.92]
De Sloovere 2017l	39	32	1	64	ELISA	IBD vs non-IBD	164.0	secondary	EK-CAL	0.97 [0.87, 1.00]	0.67 [0.56, 0.76]
Mowat 2016b	23	165	9	529	ELISA	IBD vs non-IBD	200.0	secondary	EK-CAL	0.72 [0.53, 0.86]	0.76 [0.73, 0.79]
De Sloovere 2017v	30	7	10	89	CLIA	IBD vs non-IBD	211.0	secondary	Diasorin	0.75 [0.59, 0.87]	0.93 [0.86, 0.97]
De Sloovere 2017q	35	20	5	76	PETIA	IBD vs non-IBD	243.0	secondary	fCal Turbo	0.88 [0.73, 0.96]	0.79 [0.70, 0.87]
Oyaert 2017l	21	3	0	62	PETIA	IBD vs non-IBD	285.0	secondary	fCal Turbo	1.00 [0.84, 1.00]	0.95 [0.87, 0.99]
De Sloovere 2017m	36	21	4	57	ELISA	IBD vs non-IBD	311.0	secondary	EK-CAL	0.90 [0.76, 0.97]	0.73 [0.62, 0.82]
De Sloovere 2017r	29	9	11	87	PETIA	IBD vs non-IBD	358.0	secondary	fCal Turbo	0.72 [0.56, 0.85]	0.91 [0.83, 0.96]
Oyaert 2017j	21	2	0	63	ELISA	IBD vs non-IBD	371.0	secondary	Euroimmun	1.00 [0.84, 1.00]	0.97 [0.89, 1.00]
Oyaert 2017b	18	0	3	65	FEIA	IBD vs non-IBD	376.0	secondary	EliA	0.86 [0.64, 0.97]	1.00 [0.94, 1.00]
Oyaert 2017h	21	1	0	46	ELISA	IBD vs non-IBD	477.0	secondary	Orgentec	1.00 [0.84, 1.00]	0.98 [0.89, 1.00]
De Sloovere 2017n	28	11	12	85	ELISA	IBD vs non-IBD	919.0	secondary	EK-CAL	0.70 [0.53, 0.83]	0.89 [0.80, 0.94]

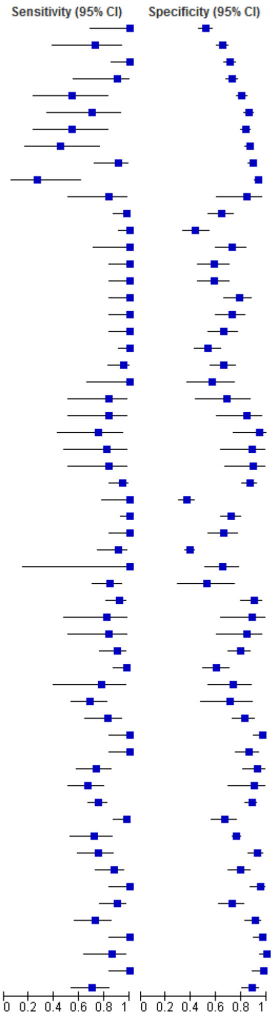

Forest plots of studies reporting data for clinical questions IBD vs non-IBD and Inflammatory vs non-inflammatory intestinal disease (16 studies)

Study	TP	FP	FN	TN	test type	Clin question	cut-off in µg/g	Setting	test name	Sensitivity (95% CI)	Specificity (95% CI)
Lee 2013	19	32	4	67	ELISA	organic vs non-organic	60.0	mix	NR	0.83 [0.61, 0.95]	0.68 [0.58, 0.77]
Hogberg 2017d	25	160	1	187	ELISA	organic vs other	20.0	primary	Calpro	0.96 [0.80, 1.00]	0.54 [0.48, 0.59]
Hogberg 2017e	20	89	6	258	ELISA	organic vs other	50.0	primary	Calpro	0.77 [0.56, 0.91]	0.74 [0.69, 0.79]
Pavlidis 2013a	77	199	17	669	ELISA	organic vs non-organic	50.0	primary	EK-CAL	0.82 [0.73, 0.89]	0.77 [0.74, 0.80]
Conroy 2017f	21	127	6	256	ELISA	organic vs non-organic	50.0	primary	IDK	0.78 [0.58, 0.91]	0.67 [0.62, 0.72]
Hogberg 2017f	12	46	14	301	ELISA	organic vs other	100.0	primary	Calpro	0.46 [0.27, 0.67]	0.87 [0.83, 0.90]
Pavlidis 2013b	73	78	21	790	ELISA	organic vs non-organic	100.0	primary	EK-CAL	0.78 [0.68, 0.86]	0.91 [0.89, 0.93]
Turvill 2016a	20	44	6	192	ELISA	organic vs non-organic	100.0	primary	EK-CAL	0.77 [0.56, 0.91]	0.81 [0.76, 0.86]
Pavlidis 2013c	67	43	27	825	ELISA	organic vs non-organic	125.0	primary	EK-CAL	0.71 [0.61, 0.80]	0.95 [0.93, 0.96]
Pavlidis 2013d	65	26	29	842	ELISA	organic vs non-organic	150.0	primary	EK-CAL	0.69 [0.59, 0.78]	0.97 [0.96, 0.98]
Pavlidis 2013e	60	9	34	859	ELISA	organic vs non-organic	200.0	primary	EK-CAL	0.64 [0.53, 0.73]	0.99 [0.98, 1.00]
Pavlidis 2013f	54	9	40	859	ELISA	organic vs non-organic	249.0	primary	EK-CAL	0.57 [0.47, 0.68]	0.99 [0.98, 1.00]
Turvill 2016b	16	14	10	222	ELISA	organic vs non-organic	250.0	primary	EK-CAL	0.62 [0.41, 0.80]	0.94 [0.90, 0.97]
Banerjee 2015g	21	48	0	50	ELISA	organic vs non-organic	8.0	secondary	IDK	1.00 [0.84, 1.00]	0.51 [0.41, 0.61]
Kennedy 2015e	139	289	17	277	ELISA	organic vs non-organic	20.0	secondary	PhiCal	0.89 [0.83, 0.94]	0.49 [0.45, 0.53]
Burri 2013c	84	16	43	218	ELISA	organic vs other	22.4	secondary	PhiCal	0.66 [0.57, 0.74]	0.93 [0.89, 0.96]
Banerjee 2015h	20	46	1	52	ELISA	organic vs non-organic	25.0	secondary	IDK	0.95 [0.76, 1.00]	0.53 [0.43, 0.63]
Carroccio 2003c	19	8	11	32	ELISA	organic vs non-organic	50.0	secondary	Calprest	0.63 [0.44, 0.80]	0.80 [0.64, 0.91]
Burri 2013a	111	13	32	249	ELISA	organic vs other	50.0	secondary	EK-CAL	0.78 [0.70, 0.84]	0.95 [0.92, 0.97]
Damms 2008c	52	13	14	43	ELISA	organic vs non-organic	50.0	secondary	EK-CAL	0.79 [0.67, 0.88]	0.77 [0.64, 0.87]
Kok 2012a	73	149	25	132	ELISA	organic vs non-organic	50.0	secondary	EK-CAL	0.74 [0.65, 0.83]	0.47 [0.41, 0.53]
Mowat 2016c	76	374	25	251	ELISA	organic vs non-organic	50.0	secondary	EK-CAL	0.75 [0.66, 0.83]	0.40 [0.36, 0.44]
Sostres 2017	27	63	10	71	FEIA	organic vs non-organic	50.0	secondary	EliA	0.73 [0.56, 0.86]	0.53 [0.44, 0.62]
Banerjee 2015i	19	39	2	59	ELISA	organic vs non-organic	50.0	secondary	IDK	0.90 [0.70, 0.99]	0.60 [0.50, 0.70]
Tibble 2002	234	71	29	268	ELISA	organic vs non-organic	50.0	secondary	NR	0.89 [0.85, 0.92]	0.79 [0.74, 0.83]
Burri 2013b	55	4	72	230	ELISA	organic vs other	50.0	secondary	PhiCal	0.43 [0.35, 0.52]	0.98 [0.96, 1.00]
El-Badry 2010e	19	3	0	17	ELISA	organic vs non-organic	50.0	secondary	PhiCal	1.00 [0.82, 1.00]	0.85 [0.62, 0.97]
Kennedy 2015f	123	149	33	417	ELISA	organic vs non-organic	50.0	secondary	PhiCal	0.79 [0.72, 0.85]	0.74 [0.70, 0.77]
Turvill 2012	91	39	18	482	ELISA	organic vs non-organic	50.0	secondary	PhiCal	0.83 [0.75, 0.90]	0.93 [0.90, 0.95]
Damms 2008d	53	16	13	40	POCT	organic vs non-organic	50.0	secondary	Prevista	0.80 [0.69, 0.89]	0.71 [0.58, 0.83]
Kok 2012b	63	131	36	150	POCT	organic vs non-organic	50.0	secondary	Quantum-Blue	0.64 [0.53, 0.73]	0.53 [0.47, 0.59]
Lizvan 2015	32	1	4	15	POCT	organic vs non-organic	50.0	secondary	Quantum-Blue	0.89 [0.74, 0.97]	0.94 [0.70, 1.00]
Kennedy 2015g	114	112	42	454	ELISA	organic vs non-organic	70.0	secondary	PhiCal	0.73 [0.65, 0.80]	0.80 [0.77, 0.83]
Banerjee 2015j	15	25	6	73	ELISA	organic vs non-organic	75.0	secondary	IDK	0.71 [0.48, 0.89]	0.74 [0.65, 0.83]
Carroccio 2003d	13	3	17	37	ELISA	organic vs non-organic	100.0	secondary	Calprest	0.43 [0.25, 0.63]	0.93 [0.80, 0.98]
Banerjee 2015k	14	18	7	80	ELISA	organic vs non-organic	100.0	secondary	IDK	0.67 [0.43, 0.85]	0.82 [0.73, 0.89]
El-Badry 2010f	15	0	4	20	ELISA	organic vs non-organic	100.0	secondary	PhiCal	0.79 [0.54, 0.94]	1.00 [0.83, 1.00]
Kennedy 2015h	109	74	47	492	ELISA	organic vs non-organic	100.0	secondary	PhiCal	0.70 [0.62, 0.77]	0.87 [0.84, 0.90]
Shitrit 2007	21	5	7	39	ELISA	organic vs non-organic	150.0	secondary	Calprest	0.75 [0.55, 0.89]	0.89 [0.75, 0.96]
Banerjee 2015l	13	14	8	84	ELISA	organic vs non-organic	150.0	secondary	IDK	0.62 [0.38, 0.82]	0.86 [0.77, 0.92]
Mowat 2016d	44	144	57	481	ELISA	organic vs non-organic	200.0	secondary	EK-CAL	0.44 [0.34, 0.54]	0.77 [0.73, 0.80]
Garcia 2006	62	22	11	95	ELISA	organic vs other	217.0	secondary	Calprest	0.85 [0.75, 0.92]	0.81 [0.73, 0.88]

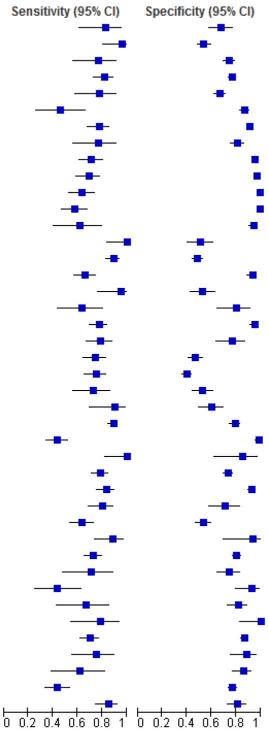

Forest plots of studies reporting data for clinical questions organic intestinal disease vs non-organic intestinal disease and organic intestinal disease vs other intestinal conditions (19 studies)

Supplement 8 2x2 table data of studies included in meta-analysis by test (50µg/g threshold) (Letters following the year indicate different 2x2 table data from the same study reference)

EK-CAL (6 studies)

Study ID	Year of study	TP	FP	TN	FN	Test type	Test name
Burri 2013a	2013	111	13	249	32	ELISA	EK-CAL
Damms 2008c	2008	52	13	43	14	ELISA	EK-CAL
De Sloovere 2017a	2017	46	55	61	0	ELISA	EK-CAL
Kok 2012a	2012	73	149	132	25	ELISA	EK-CAL
Mowat 2016 a	2016	29	421	273	3	ELISA	EK-CAL
Pavlidis 2013a	2013	77	199	669	17	ELISA	EK-CAL

ELiA (5 studies)

Study ID	Year of study	TP	FP	TN	FN	Test type	Test name
Jang 2016a	2016	30	1	4	1	FEIA	ELiA
Labaere 2014b	2014	9	1	18	3	FEIA	ELiA
Oyaert 2014a	2014	48	17	115	3	FEIA	ELiA
Oyaert 2017a	2017	21	22	43	0	FEIA	ELiA
Sostres 2017	2017	27	63	71	10	FEIA	ELiA

PhiCal (8 studies)

Study ID	Year of study	TP	FP	TN	FN	Test type	Test name
Burri 2013b	2013	55	4	230	72	ELISA	PhiCal
Dhaliwal 2015	2015	126	33	115	18	ELISA	PhiCal
El-Badry 2010c	2010	9	13	17	0	ELISA	PhiCal
Kennedy 2015b	2015	88	149	417	3	ELISA	PhiCal
Li 2006	2006	55	6	54	5	ELISA	PhiCal
Otten 2008a	2008	22	12	79	1	ELISA	PhiCal
Schoepfer 2008	2008	53	0	30	11	ELISA	PhiCal
Turvill 2012	2012	91	39	482	18	ELISA	PhiCal

Quantum-Blue (5 studies)

Study ID	Year of study	TP	FP	TN	FN	Test type	Test name
Jang 2016b	2016	31	1	4	0	POCT	Quantum-Blue
Kok 2012b	2012	63	131	150	36	POCT	Quantum-Blue
Labaere 2014e	2014	10	6	13	2	POCT	Quantum-Blue
Lizvan 2015	2015	32	1	15	4	POCT	Quantum-Blue
Oyaert 2014b	2014	51	37	95	0	POCT	Quantum-Blue

Supplement 9 2x2 table data of studies included in meta-analysis by clinical question (50μg/g threshold)

IBD versus IBS (11 studies)

Study ID	Year of study	TP	FP	TN	FN	Clinical question
Banerjee 2015	2015	12	39	59	0	IBD vs IBS
Carroccio 2003	2003	11	8	32	0	IBD vs IBS
Caviglia 2014	2014	24	10	11	0	IBD vs IBS
Damms 2008	2008	18	12	44	0	IBD vs functional
De Sloovere 2017	2017	46	55	61	0	IBD vs IBS
Dhaliwal 2015	2015	126	33	115	18	IBD vs IBS
El-Badry 2010	2010	9	3	17	0	IBD vs IBS
Jang 2016	2016	30	1	4	1	IBD vs IBS
Kennedy 2015	2015	88	149	417	3	IBD vs functional
Otten 2008	2008	22	12	79	1	IBD vs IBS
Schoepfer 2008	2008	53	0	30	11	IBD vs IBS

IBD versus non-IBD (14 studies)

Study ID	Year of study	TP	FP	TN	FN	Clinical question
Alrubaiy 2012	2012	2	18	34	0	Infl vs non-infl
Boyd 2016	2016	23	117	284	0	IBD vs non-IBD
Carroccio 2003	2003	11	16	43	0	IBD vs non-IBD
Caviglia 2014	2014	38	10	11	7	Infl vs non-infl
Conroy 2017	2017	8	140	259	3	IBD vs non-IBD
De Sloovere 2017	2017	38	33	63	2	IBD vs non-IBD
El-Badry 2010	2010	9	13	17	0	IBD vs non-IBD
Hogberg 2017	2017	9	100	263	1	IBD vs non-IBD
Labaere 2014	2014	9	1	18	3	IBD vs non-IBD
Li 2006	2006	55	6	54	5	Infl vs non-infl
Mowat 2016	2016	29	421	273	3	IBD vs non-IBD
Oyaert 2014	2014	51	37	95	0	IBD vs non-IBD
Oyaert 2017	2017	21	18	47	0	IBD vs non-IBD
Tan 2016	2016	15	165	95	0	IBD vs non-IBD

Organic versus non-organic (15 studies)

Study ID	Year of study	TP	FP	TN	FN	Clinical question
Banerjee 2015	2015	19	39	59	2	organic vs non-organic
Burri 2013	2013	111	13	249	32	organic vs other
Carroccio 2003	2003	19	8	32	11	organic vs non-organic
Conroy 2017	2017	21	127	256	6	organic vs non-organic
Damms 2008	2008	53	16	40	13	organic vs non-organic
El-Badry 2010	2010	19	3	17	0	organic vs non-organic
Hogberg 2017	2017	20	89	258	6	organic vs other
Kennedy 2015	2015	123	149	417	33	organic vs non-organic
Kok 2012	2012	63	131	150	36	organic vs non-organic
Lizvan 2015	2015	32	1	15	4	organic vs non-organic
Mowat 2016	2016	76	374	251	25	organic vs non-organic

Pavlidis 2013	2013	77	199	669	17	organic vs non-organic
Sostres 2017	2017	27	63	71	10	organic vs non-organic
Tibble 2002	2002	234	71	268	29	organic vs non-organic
Turvill 2012	2012	91	39	482	18	organic vs non-organic

Supplement 10 2x2 table data at 100μg/g threshold by clinical question

IBD versus IBS (5 studies)

Study ID	Year of study	TP	FP	TN	F N	Clinical question	Threshold μg/g
Banerjee 2015	2015	11	18	80	1	IBD vs IBS	100
Caviglia 2014	2014	21	6	15	3	IBD vs IBS	100
El-Badry 2010	2010	7	0	20	2	IBD vs IBS	100
Kennedy 2015	2015	87	74	492	4	IBD vs functional	100
Rosenfeld 2016	2016	4	5	41	0	IBD vs IBS	100

IBD versus non-IBD (5 studies)

Study ID	Year of study	TP	FP	TN	FN	Clinical question	Threshold μg/g
Caviglia 2014	2014	31	6	15	14	Infl vs non-infl	100
Conroy 2017	2017	6	78	321	5	IBD vs non-IBD	100
El-Badry 2010	2010	7	8	22	2	IBD vs non-IBD	100
Hogberg 2017	2017	7	51	312	3	IBD vs non-IBD	100
Limburg 2000	2000	24	14	67	5	Infl vs non-infl	100

Organic versus non-organic (7 studies)

Study ID	Year of study	TP	FP	TN	FN	Clinical question	Threshold μg/g
Banerjee 2015	2015	14	18	80	7	organic vs non-organic	100
Carroccio 2003	2003	13	3	37	17	organic vs non-organic	100
El-Badry 2010	2010	15	0	20	4	organic vs non-organic	100
Hogberg 2017	2017	12	46	301	14	organic vs other	100
Kennedy 2015	2015	109	74	492	47	organic vs non-organic	100
Pavlidis 2013	2013	73	78	790	21	organic vs non-organic	100
Turvill 2016	2016	20	44	192	6	organic vs non-organic	100

Supplement 11 2x2 table data at 50µg/g threshold of 28 studies considered for exploratory sensitivity analysis

Study ID	Year of study	TP	FP	TN	FN
Alrubaiy 2012	2012	2	18	34	0
Banerjee 2015c*	2015	12	39	59	0
Banerjee 2015i	2015	19	39	59	2
Boyd 2016a	2016	23	117	284	0
Burri 2013a	2013	111	13	249	32
Burri 2013b	2013	55	4	230	72
Carroccio 2003a	2003	11	8	32	0
Carroccio 2003b	2003	11	16	43	0
Carroccio 2003c	2003	19	8	32	11
Caviglia 2014a	2014	24	10	11	0
Caviglia 2014d	2014	38	10	11	7
Conroy 2017a	2017	8	140	259	3
Conroy 2017f	2017	21	127	256	6
Damms 2008a	2008	18	12	44	0
Damms 2008b	2008	16	11	45	2
Damms 2008c	2008	52	13	43	14
Damms 2008d	2008	53	16	40	13
De Sloovere 2017a	2017	46	55	61	0
De Sloovere 2017d	2017	46	30	86	0
De Sloovere 2017h	2017	43	14	102	3
De Sloovere 2017k	2017	40	54	42	0
De Sloovere 2017o	2017	40	45	51	0
De Sloovere 2017t	2017	38	33	63	2
Dhaliwal 2015	2015	126	33	115	18
El-Badry 2010a	2010	9	3	17	0
El-Badry 2010c	2010	9	13	17	0
El-Badry 2010e	2010	19	3	17	0
Hogberg 2017b	2017	9	100	263	1
Hogberg 2017e	2017	20	89	258	6
Jang 2016a	2016	30	1	4	1
Jang 2016b	2016	31	1	4	0
Jang 2016c	2016	25	0	5	6
Kennedy 2015b	2015	88	149	417	3
Kennedy 2015f	2015	123	149	417	33
Kok 2012a	2012	73	149	132	25
Kok 2012b	2012	63	131	150	36
Labaere 2014b	2014	9	1	18	3
Labaere 2014c	2014	9	2	15	2
Labaere 2014d	2014	10	2	17	2
Labaere 2014e	2014	10	6	13	2
Labaere 2014g	2014	10	3	16	2
Li 2006	2006	55	6	54	5
Lizvan 2015	2015	32	1	15	4

Mowat 2016a	2016	29	421	273	3
Mowat 2016c	2016	76	374	251	25
Otten 2008a	2008	22	12	79	1
Oyaert 2014a	2014	48	17	115	3
Oyaert 2014b	2014	51	37	95	0
Oyaert 2017a	2017	21	22	43	0
Oyaert 2017c	2017	21	18	47	0
Oyaert 2017e	2017	21	14	51	0
Oyaert 2017g	2017	21	27	38	0
Oyaert 2017i	2017	21	27	38	0
Oyaert 2017k	2017	21	22	43	0
Pavlidis 2013a	2013	77	199	669	17
Schoepfer 2008	2008	53	0	30	11
Sostres 2017	2017	27	63	71	10
Tan 2016	2016	15	165	95	0
Tibble 2002	2002	234	71	268	29
Turvill 2012	2012	91	39	482	18

*Letters indicate different 2x2 table data from the same study reference
