# Supplementary material for: Faecal calprotectin to detect inflammatory bowel disease: a systematic review and exploratory meta-analysis of test accuracy
Source: BMJ Open. 2019 Mar 8;9(3):e027428. doi: 10.1136/bmjopen-2018-027428 (PMC6429840; doi:10.1136/bmjopen-2018-027428)
Supplement: Author's manuscript [file bmjopen-2018-027428.draft_revisions.pdf]

BMJ Open

BMJ Open is committed to open peer review. As part of this commitment we make the peer review history of every article we publish publicly available.

When an article is published we post the peer reviewers' comments and the authors' responses online. We also post the versions of the paper that were used during peer review. These are the versions that the peer review comments apply to.

The versions of the paper that follow are the versions that were submitted during the peer review process. They are not the versions of record or the final published versions. They should not be cited or distributed as the published version of this manuscript.

BMJ Open is an open access journal and the full, final, typeset and author-corrected version of record of the manuscript is available on our site with no access controls, subscription charges or pay-per-view fees (http://bmjopen.bmj.com).

If you have any questions on BMJ Open's open peer review process please email info.bmjopen@bmj.com

Faecal calprotectin to detect inflammatory bowel disease - A systematic review and exploratory meta-analysis of test accuracy

Journal:	*BMJ Open*
Manuscript ID	bmjopen-2018-027428
Article Type:	Research
Date Submitted by the Author:	22-Oct-2018

Complete List of Authors:	Freeman, Karoline; University of Warwick, Warwick Medical School<br>Willis, Brian; University of Birmingham, Institute of Applied Health Research<br>Fraser, Hannah; University of Warwick, Warwick Medical School<br>Taylor-Phillips, Sian; University of Warwick, Warwick Medical School<br>Clarke, Aileen; University of Warwick, Division of Health Sciences

Keywords:	Inflammatory bowel disease < GASTROENTEROLOGY, PRIMARY CARE, Protocols & guidelines < HEALTH SERVICES ADMINISTRATION & MANAGEMENT

SCHOLARONE™
Manuscripts

Faecal calprotectin to detect inflammatory bowel disease - A systematic review and exploratory meta-analysis of test accuracy

Karoline Freeman, Brian H Willis, Hannah Fraser, Sian Taylor-Phillips, Aileen Clarke

Karoline Freeman NIHR Doctoral Fellow Division of Health Sciences, University of Warwick, Coventry, CV4 7AL, UK

Brian H Willis MRC Clinician Scientist Primary Care Clinical Sciences, Institute of Applied Health Research, University of Birmingham, Edgbaston, Birmingham, B15 2TT, UK

Hannah Fraser Research Associate Division of Health Sciences, University of Warwick, Coventry, CV4 7AL, UK

Sian Taylor-Phillips Associate Professor of Screening and Test Evaluation Division of Health Sciences, University of Warwick, Coventry, CV4 7AL, UK

Aileen Clarke Professor of Public Health & Health Services Research Division of Health Sciences, University of Warwick, Coventry, CV4 7AL, UK

Correspondence to:

Karoline Freeman, Division of Health Sciences, University of Warwick, Coventry, CV4 7AL, UK
Email: k.freeman@warwick.ac.uk

Word Count: 3385

For peer review only

For peer review only - http://bmjopen.bmj.com/site/about/guidelines.xhtml

ABSTRACT

Objective

Test accuracy of faecal calprotectin (FC) testing in primary care is inconclusive. We aimed to assess test accuracy of FC testing in primary care and compare it to secondary care estimates for the detection of inflammatory bowel disease (IBD).

Methods

Systematic review and meta-analysis of test accuracy using a bivariate random effects model. We searched MEDLINE, EMBASE, Cochrane Library and Web of Science until 31/05/2017 and included studies from auto alerts up until 31/01/2018. Eligible studies measured FC levels in stool samples to detect IBD in adult patients with chronic (at least 6-8 weeks) abdominal symptoms in primary or secondary care. Risk of bias and applicability were assessed using the Quality Assessment of Diagnostic Accuracy Studies-2 criteria. We followed the protocol registered as PROSPERO CRD 42012003287.

Results

38 out of 2168 studies were eligible including five from primary care. Comparison of test accuracy by setting was precluded by extensive heterogeneity. Overall summary estimates of sensitivity and specificity were not recorded. At a threshold of 50μg/g, sensitivity from separate meta-analysis of four assay types ranged from 0.85 (95% CI 0.75-0.92) to 0.94 (0.75-0.90) and specificity from 0.67 (0.56-0.76) to 0.88 (0.77-0.94). Across three different definitions of disease, sensitivity ranged from 0.80 (0.76-0.84) to 0.97 (0.91-0.99) and specificity from 0.67 (0.58-0.75) to 0.76 (0.66-0.84). Sensitivity appears to be lower in primary care and is further reduced at a revised threshold of 100μg/g.

Conclusions

Conclusive estimates of sensitivity and specificity of FC testing in primary care for the detection of IBD are still missing. There is insufficient evidence in the published literature to support the decision to introduce FC testing in primary care. Studies evaluating FC testing in an appropriate primary care setting are needed.

For peer review only - http://bmjopen.bmj.com/site/about/guidelines.xhtml

Strengths and limitations of this study

- This review used an innovative approach involving random combinations of test accuracy data from included studies in 25,000 meta-analyses to display the breadth of heterogeneous evidence from included studies.
- Exploratory meta-analyses investigated different test assays, clinical questions and positivity thresholds of faecal calprotectin levels.
- No overall summary estimates of sensitivity and specificity were produced due to heterogeneity.
- Comparison of test accuracy by setting was precluded by extensive heterogeneity in the small number of studies in primary care.
- The categorisation into different clinical questions was subjective because the disease categories are ill defined and studies' definitions of conditions and groups of conditions varied.

For peer review only

For peer review only - http://bmjopen.bmj.com/site/about/guidelines.xhtml

INTRODUCTION

Inflammatory bowel disease (IBD) is an organic disease caused by inflammation of the intestine. The disease is severe and progressive,[1, 2] and over 50% of people require surgery within 10 years of diagnosis.[3] Timely referral, which requires filtering of patients with a high probability of IBD from a broader group of patients with mainly irritable bowel syndrome (IBS), is vital. As many as 3.1 million people in the United States [4] and 2.5–3 million people in Europe [5] suffer from IBD with close to 300,000 patients in the UK.[6] An estimated 256,000 new cases of IBD are diagnosed annually throughout Europe.[5] Northern America and Northern Europe, including the UK, have the highest incidence rates.[7]

General practitioners refer between 10-20% of patients presenting with abdominal symptoms if no specific test is available.[8, 9] However, only about 25% of referred patients have organic disease, of which a third have IBD.[10, 11] Faecal calprotectin (FC), a regulator of the inflammatory response, could potentially aid more selective referral. Patients with high levels of FC have an increased probability of IBD. FC is a small calcium-binding protein. It is released into the intestinal lumen from activated neutrophils accumulating at the site of inflammation, and levels of FC are correlated with the level of inflammation.[12] The protein is stable at room temperature and resistant to digestion, making it a relatively easy candidate for a stool test for inflammation of the intestinal tract.

The FC test is FDA approved and recommended by gastroenterological societies across the globe for its usefulness in the diagnosis of IBD.[13-16] However, there is no clear guidance on which settings it is considered appropriate. FC testing is not supported by Medicare in the USA and Australia, but is approved for the differential diagnosis of IBD and IBS in adult patients in the UK by the National Institute for Health and Care Excellence (NICE) when referral to secondary care is being considered and cancer is not suspected (DG11).[17] At the time of the recommendations only test accuracy studies undertaken in secondary care were available to inform the decision.[18] Our knowledge on how the test performs in primary care is limited and a systematic approach to assessing the applicability of the available evidence specifically for this setting is timely. We aimed to assess the test accuracy of FC testing to detect IBD in adult patients with chronic abdominal symptoms in a primary care pathway (Figure 1). In addition we aimed to explore the differences in FC test performance between primary and secondary care.

++ Figure 1 ++

For peer review only - http://bmjopen.bmj.com/site/about/guidelines.xhtml

METHODS

Review methods

We followed the protocol by Waugh et.al.[18] registered as PROSPERO CRD 42012003287. We considered all studies included by Waugh et al.[18] for inclusion and searched MEDLINE, EMBASE, Cochrane Library and Web of Science from 01/09/2012 to 31/05/2017 (Supplement 1). Eligible studies from auto-alerts were included up until 31/01/2018. Reference lists of included studies were checked.

Studies were assessed for eligibility independently and in duplicate, and any disagreements were resolved by consensus. Records rejected at full text stage and reasons for exclusion were documented (Supplement 2).

We included studies which measured FC levels in stool samples to detect IBD in adult patients (with ≥80% of study population 18-60 years) with chronic (at least 6-8 weeks) abdominal symptoms not yet diagnosed in primary or secondary care. The reference standard to verify FC test outcomes was colonoscopy with histology, other imaging technologies and follow-up. Our main outcomes of interest were sensitivity and specificity.

Data were extracted by KF on pre-specified data extraction sheets (Supplement 3) which were checked by HF. For studies reporting sensitivity, specificity, positive and negative predictive values and total number of included patients, 2x2 tables of true positives (TP), false positives (FP), false negatives (FN), and true negatives (TN) were calculated. For studies reporting test accuracy at a lower and an upper cut-off, 2x2 data tables were extracted for the lower and the upper cut-off. Authors of relevant studies with missing 2x2 data were contacted to request data.

Quality was assessed independently and in duplicate by two reviewers using the Quality Assessment of Diagnostic Accuracy Studies-2 criteria (QUADAS-2)[19] which included definitions for the signalling questions to match the review question.

Patient involvement

For peer review only - http://bmjopen.bmj.com/site/about/guidelines.xhtml

A patient advisory group was actively involved in the funding application for the project of which this study is a part. Two representatives of the patient advisory group will be involved in dissemination of findings at local and national events for IBD patients.

Analysis

Studies were considered under three main clinical questions with different definitions of the target and non-target conditions: FC testing to differentiate: 1) IBD from IBS, 2) IBD from non-IBD conditions and 3) organic from non-organic conditions. Different FC test assays were handled independently.

We used Review Manager 5.3 (The Cochrane Collaboration, The Nordic Cochrane Centre, Copenhagen, Denmark) to produce paired forest plots of sensitivity and specificity. All analyses were performed in R version 3.4.1(Vienna, Austria).[20] Meta-analyses were undertaken using a bivariate random effects model[21] using the package *glmer*[22] with a minimum of five studies required for meta-analysis. Different assays and clinical questions were considered separately. No overall summary estimates of sensitivity and specificity were produced due to heterogeneity. Test accuracy was explored at the commonly used thresholds of 50 and 100µg faecal calprotectin per g stool sample (µg/g). Outputs from meta-analyses were entered into Review Manager to produce receiver operating characteristic (ROC) plots.

Heterogeneity was explored by meta-regression analyses with assay type and clinical question added as covariates in turn, assuming equal variances due to small numbers of studies per subgroup.

Each meta-analysis considered only one outcome per study. However, a number of studies reported 2x2 data for multiple test assays and clinical questions. We therefore undertook an exploratory sensitivity analysis. First we produced a list of all possible combinations of test assay and clinical question for each included study at a common threshold (some studies reported outcomes for one test assay and clinical question contributing one possible combination while others reported results for up to three test assays and two clinical questions resulting in 6 possible combinations). We then ran a random sample of 25,000 meta-analyses out of a possible 10 million picking one outcome per study at random in each round of meta-analysis and considering all combinations of test assays and clinical questions reported in the studies. Pairs of sensitivity and specificity from these meta-analyses were plotted in an ROC

For peer review only - http://bmjopen.bmj.com/site/about/guidelines.xhtml

plot and a 2D density plot to take account of the different definitions of disease and test assays and to allow visualisation of the variability in the data.

RESULTS

Figure 2 provides the PRISMA[23] flow diagram of study selection. Out of 2168 unique records 38 studies were eligible for inclusion. The summary Table 1 reveals extensive heterogeneity in all major aspects of test accuracy studies. Study characteristics by study can be found in Supplement 4.

++ Figure 2 ++

Table 1 Summary of study characteristics of included studies addressing the test accuracy question of FC testing in the detection of IBD

Study characteristic	Outcome
**Study characteristics:**	
Publication type (studies)	
Full text	32
Abstract	6
Year of publication (range)	2000-2018
Population size (range)	31-1031
**Geographical region (studies):**	
UK	14
Rest of Europe	16
Asia	2
Middle East	2
North Africa	1
Russia	1
Canada	1
USA	1
**Patient characteristics:**	
Age (range)	14-97 years
IBD prevalence (range)	2.1% to 76%
**FC assay type (studies)*:**	
Immunoassays	
ELISA	32
FEIA	5
CLIA	3
PETIA	2
POCT	10
**Setting (studies)****	
Primary care	5
Secondary care	29
Out- and inpatients	12
Referred patients	17
Mix	2

For peer review only - http://bmjopen.bmj.com/site/about/guidelines.xhtml

Target condition (studies)*:	
IBD	23
Inflammatory disease	5
Organic disease	19
**Non-target condition (studies)*:**	
IBS	12
Functional disease	2
Non-IBD	12
Non-organic disease	16
Non-inflammatory disease	5
Other	3
**FC test data collection (studies):**	
Prospectively for patients with eligible symptoms in primary care	1
Prospectively for patients at time of referral in primary care	1
Retrospectively of routine FC tests in primary or secondary care	10
Prospectively prior to a planned colonoscopy in secondary care	23
Prospectively during the assessment for the need of colonoscopy in secondary care	1
Unclear	2
**Reference standard (studies):**	
Colonoscopy with biopsy	13
Colonoscopy +/- biopsy	7
Endoscopy + other imaging tests	8
Endoscopy + follow-up	3
Endoscopy + other imaging tests +follow-up	5
Unclear	2

CLIA - chemiluminescent immunoassay, PETIA – particle enhanced turbimetric immunoassay, ELISA - enzyme-linked immunosorbent assay (=EIA enzyme immunoassay), FEIA - fluorescence enzyme immunoassay, POCT – point of care test, IBD – inflammatory bowel disease
*some studies evaluated multiple tests/clinical questions
**Two studies were unclear about the setting

Figure 3 summarises the assessment of risk of bias and applicability (Supplement 5 for the assessment by study). Overall, the risk of bias was high or uncertain across all four domains in at least 50% of studies. Concerns about the applicability of the patient population to a primary care setting were high in about 75% of studies.

++ Figure 3 ++

The Forest plots in Supplement 6 describe the complete evidence on test accuracy from the 38 included studies reporting all the thresholds, assays and clinical questions explored. Overall, as might be expected, sensitivity decreases and specificity increases as the threshold increases. FC testing appears to be more accurate in the detection of the more precise clinical construct of IBD than in the detection of organic disease as a whole.

For peer review only - http://bmjopen.bmj.com/site/about/guidelines.xhtml

Two studies[24, 25] compared 5 and 6 different test assays at a common threshold of 50μg/g. This allows for a test comparison in the same study population under similar study conditions. It appears that the specificity varies to a greater extent than the sensitivity (Figure 4). Both studies reported medium to strong correlations but low agreement between assays, meaning that analysing the same sample with different FC test assays will result in different values for FC.

++ Figure 4 ++

Meta-analysis at 50μg/g threshold

28/38 studies reported test accuracy at the common 50μg/g cut-off and could be considered for meta-analysis. Meta-analyses were undertaken separately for test assays and for clinical questions to explore heterogeneity and to allow for multiple outcomes from a number of studies.

All 18 assays were considered in the meta-analysis investigating effect of assay type on summary estimates of test accuracy. (This was based on the assumption that the clinical question is generic and one outcome was picked per study for analysis). Four assays (PhiCal, EK-CAL, Quantum-Blue and EliA) with 5 or more studies each could be considered in the comparison (Supplement 7 for 2x2 data). 19 studies contributed data.[10, 24-41] Five studies [10, 25, 31, 39, 40] contributed data to two different assays. Figure 5 depicts the pairs of sensitivity and false positive rates of contributing studies in the ROC space, with summary estimates by assay type. At the global 50μg/g threshold, test performance appeared to vary slightly across assays (Table 2). Quantum-Blue had the highest summary estimate for sensitivity with 0.94 (95% CI 0.75-0.99) but also the lowest specificity (0.67, 95% CI 0.56-0.76). The greatest difference in sensitivity (9%) was between EK-CAL ELISA and Quantum-Blue POCT while the greatest difference in specificity (21%) was between PhiCal ELISA and Quantum-Blue POCT suggesting greater variation in specificity than in sensitivity across assays. Whether these differences are due to differing performance of test assays or due to methodological issues in study design is difficult to ascertain. The differences are large enough to suggest that test assays should not be treated as equivalent.

++ Figure 5 ++

Table 2 Comparison of results of separate meta-analyses and meta-regression with test type and clinical question as covariates at two thresholds (numbers in brackets are numbers of studies meta-analysed)

**Test comparison at 50μg/g threshold**

For peer review only - http://bmjopen.bmj.com/site/about/guidelines.xhtml

Method	PhiCal (n=8)		EK-CAL (n=6)		Quantum-Blue (n=5)		ELiA (n=5)	
	Sensitivity (95% CI)	Specificity (95% CI)	Sensitivity (95% CI)	Specificity (95% CI)	Sensitivity (95% CI)	Specificity (95% CI)	Sensitivity (95% CI)	Specificity (95% CI)
Separate MA for each test	0.89 (0.76; 0.95)	0.88 (0.77; 0.94)	0.85 (0.75; 0.92)	0.69 (0.47; 0.85)	0.94 (0.75; 0.90)	0.67 (0.56; 0.76)	0.92 (0.78; 0.97)	0.78 (0.60; 0.89)
Meta-regression (test) with equal variances	0.89 (0.77; 0.95)	0.89 (0.79; 0.95)	0.87 (0.71; 0.95)	0.69 (0.49; 0.84)	0.92 (0.78; 0.98)	0.73 (0.50; 0.88)	0.92 (0.77; 0.97)	0.78 (0.57; 0.91)

Comparison of clinical questions at 50µg/g threshold

Method	IBD vs IBS (n=11)		IBD vs non-IBD (n=14)		Organic vs non-organic (n=15)	
	Sensitivity (95% CI)	Specificity (95% CI)	Sensitivity (95% CI)	Specificity (95% CI)	Sensitivity (95% CI)	Specificity (95% CI)
Separate MA for each clinical question	0.97 (0.91; 0.99)	0.76 (0.66; 0.84)	0.95 (0.88; 0.98)	0.67 (0.58; 0.75)	0.80 (0.76; 0.84)	0.76 (0.66; 0.83)
Meta-regression (clinical question) with equal variances	0.96 (0.92; 0.98)	0.77 (0.66; 0.85)	0.94 (0.90; 0.97)	0.67 (0.57; 0.76)	0.81 (0.74; 0.86)	0.75 (0.67; 0.82)

Comparison of clinical questions at 100µg/g threshold

Method	IBD vs IBS (5)		IBD vs non-IBD (5)		Organic vs non-organic (7)	
	Sensitivity (95% CI)	Specificity (95% CI)	Sensitivity (95% CI)	Specificity (95% CI)	Sensitivity (95% CI)	Specificity (95% CI)
Separate MA for each clinical question	0.92 (0.85; 0.96)	0.86 (0.82; 0.89)	0.72 (0.63; 0.80)	0.82 (0.78; 0.86)	0.67 (0.56; 0.76)	0.87 (0.84; 0.90)
Meta-regression (clinical question) with equal variances	0.92 (0.85; 0.96)	0.86 (0.81; 0.89)	0.73 (0.60; 0.82)	0.82 (0.77; 0.86)	0.67 (0.58; 0.75)	0.87 (0.84; 0.89)

(MA meta-analysis, CI confidence interval, IBD inflammatory bowel disease, IBS irritable bowel syndrome)

All reported 2x2 data (Supplement 8) for the clinical questions IBD versus IBS, IBD versus non-IBD and organic versus non-organic disease were considered in the meta-analysis investigating the effects of the different clinical questions on summary estimates of test accuracy under the assumption that the test is generic. 28 studies were included at the common threshold of

For peer review only - http://bmjopen.bmj.com/site/about/guidelines.xhtml

50µg/g.[10, 24-50] A total of 10 studies[30, 32, 34, 36, 37, 43, 45-48] contributed information to more than one category. The ROC plot in Figure 6 summarises the summary estimates for the comparison of the three clinical questions and the pairs of sensitivity and false positive rates for the contributing studies. Table 2 shows that specificity for IBD versus non-IBD (0.67, 95% CI 0.58-0.75) is lower than for IBD versus IBS (0.76, 95% CI 0.66-0.84), acknowledging that confidence intervals overlap, suggesting that broadening the definition of the non-target condition may produce more false positives as other non-IBD intestinal conditions are included. On the other hand, sensitivity for organic disease (0.80, 95% CI 0.76 to 0.84) is lower than for IBD (0.97, 95% CI 0.91 to 0.99) suggesting that some organic disease will be missed with FC testing as organic conditions, including adenomas and diverticulosis, are not typically associated with inflammation. Considering different definitions of the clinical questions results in greater variation in sensitivity than specificity meaning that widening the target condition had a greater impact on test accuracy than changing the non-target condition (IBS versus non-IBD versus non-organic disease).

++ Figure 6 ++

Meta-analysis at 100µg/g threshold

11 studies[30, 32, 35, 43, 45-48, 51-53] reported test accuracy at a threshold of 100µg/g (Supplement 9 for 2x2 data). Of these, five contributed to more than one category of clinical questions. As expected raising the threshold to 100µg/g reduces sensitivity and increases specificity for all clinical questions (Table 2). Insufficient numbers of studies at the 100µg/g were available to explore assay type.

Meta-regression and exploratory sensitivity analysis

Results from separate meta-analyses and from meta-regression analyses with equal variances showed similar results (Table 2 and Supplement 10).

Ten studies reported results for multiple clinical questions and eight studies compared multiple tests. Our approach of randomly selecting one test or one clinical question per study for meta-analysis uses only one set of evidence per study. Choosing a different set might have resulted in different outcomes and conclusions as the meta-analyses suggest that it might not be appropriate to consider tests and questions alike. In an attempt to capture this variation, Figure 7 displays results of 25,000 meta-analyses of 28 studies (Supplement 11 for 2x2 data) picking

For peer review only - http://bmjopen.bmj.com/site/about/guidelines.xhtml

one outcome per study at random for each round of meta-analysis. The results show overall high sensitivity and specificity irrespective of test assay and clinical question (Panel A) with slightly greater variation in sensitivity than specificity (Panel B). Median sensitivity and specificity of the 25,000 analyses and ranges were 0.9 (min 0.85, max 0.94) and 0.76 (min 0.73, max 0.79), respectively.

++ Figure 7 ++

Primary versus secondary care

Five of the eligible studies were from primary care settings.[35, 44, 47, 48, 53] The studies were too heterogeneous for meta-analysis. In an attempt to compare test performance of FC testing in primary care with secondary care only one study qualified.[47] The study evaluated the routine use of FC testing in primary care and reported lower sensitivity of 0.72 but comparable specificity of 0.65 for differentiating IBD from non-IBD at a threshold of 50µg/g when compared to our pooled estimates across different test assays including all settings (sensitivity 0.95, CI 0.88-0.98 and specificity 0.67, CI 0.58-0.75). However, indication for testing and the place of the FC test in the patient pathway were unclear in this study and the study may suffer from differential verification bias due to concerns over the reference standard. The authors explained the low sensitivity with the fact that general practitioners may have referred patients with high suspicion of IBD without testing. Another reasonable explanation could be the greater number of apparently milder IBD cases in primary care in which FC testing is falsely negative, reducing sensitivity in this setting, and demonstrating the impact of the different spectrum of disease on test accuracy estimates .[54]

DISCUSSION

None of the 38 included studies sufficiently addressed the review question of test accuracy of FC testing in a primary care pathway. The studies recruited patients with a different spectrum of disease from that anticipated for primary care, focussed on disease groups which were broader than the intended IBD group, or did not verify all patients with the preferred reference standard. Furthermore, the evidence was highly heterogeneous. Studies varied in thresholds and assays used, as well as in target conditions and settings. Eighteen different test assays were studied, although similar they could not be considered generic for meta-analysis. Clinical questions were analysed as three distinct categories which were related but which could not be pooled for meta-analysis. A number of studies compared multiple tests and / or explored different clinical

For peer review only - http://bmjopen.bmj.com/site/about/guidelines.xhtml

questions. Picking one test and one clinical question per study for meta-analysis was unjustifiable. We favoured an innovative approach which allowed us to explore the whole breadth of evidence and showed that irrespective of clinical question and test assay, test accuracy of FC testing was high. However, the variation in specificity could translate into considerable uncertainty of false positives when scaled up to population testing, while the variation in sensitivity reflects the limited value of FC testing for other organic conditions.

The review has a number of limitations. Due to heterogeneity we decided not to pool across tests and clinical questions. Instead we conducted exploratory analyses which demonstrated that the test assay and clinical question may affect the summary sensitivity and specificity by as much as 9%. We were also unable to compare primary and secondary care studies due to the small number of heterogeneous studies in primary care. Finally, the categorisation into different clinical questions was subjective because the disease categories are ill defined and studies' definitions of conditions and groups of conditions varied.

This review is broadly in line with the approaches and interpretation of the evidence of the previous review.[18] However, Waugh et al. reported pooled estimates of sensitivity (0.93, 95% CI 0.83-0.97) and specificity (0.94, 95% CI 0.73-0.99) across tests for the differential diagnosis of IBD versus IBS for five secondary care studies and this was the basis for national decisions to introduce FC testing in primary care. When compared to our equivalent analysis of 11 studies we found comparable sensitivity (0.97, CI 0.91-0.99) but considerably lower specificity (0.76, CI 0.66-0.84) suggesting that false positives might be more of a concern than previously concluded. Thus the predicted reduction in colonoscopies and subsequent cost savings may not be realised as a result of introducing FC testing into the primary care pathway in the UK.[18] Furthermore, this review found enough disagreement between tests to caution against treating tests as equivalent. Issues in homogenisation, dilutions and extraction prior to analysis as well as lack of standardisation of different assays contribute to these differences. Recommended cut-off values would have to be determined locally until these issues are resolved.[24]

None of the primary care studies assessed FC testing for the differential diagnosis of IBD versus IBS. The comparison of IBD versus non-IBD might reflect the clinical situation for FC testing more accurately, but produces more false positives as FC levels might be raised in diverticular disease, coeliac disease, rectal adenocarcinoma, non-specific inflammation, and others.[35, 53] The meaning of the additional false positives due to non-IBD inflammatory

For peer review only - http://bmjopen.bmj.com/site/about/guidelines.xhtml

conditions is debatable in the context of clinical practice where incidental findings of true disease would not be classed as a false positive test outcome but would prompt further investigations.

NICE recently endorsed a cut-off of 100µg/g for use in primary care in England and Wales, based on a primary care study which showed a 43% reduction in FC test positives compared with a threshold of 50µg/g.[47] We investigated test accuracy at this cut-off including all settings and demonstrated an increase in specificity by at least 10%, while the magnitude of the resulting decrease in sensitivity was more uncertain.

Referral of FC tested patients was consistent in three UK primary care studies (41%,[53] 42%[35] and 48%[47]) with over 25% of FC negative patients referred. This raises concerns about the impact of FC testing on colonoscopy rates as a considerable proportion of referred patients with negative FC levels were further investigated. Inappropriate use of FC testing as a screening test rather than to rule out IBD has been proposed as an explanation.[55] However, the number of investigations of referred FC negatives varied greatly among the three studies from 19%[47] to 46%[35] and even 71%[53] suggesting perhaps unsurprisingly, that the number of potentially unnecessary colonoscopies may be at least as dependent on secondary care decision-making as on the availability of FC testing in primary care.

The applicability of the spectrum of secondary care patients to a primary care setting is questionable even if studies investigated referred patients. Firstly, general practitioners might have tested more patients than they referred and, secondly, patients referred came from different pathways including cancer pathways. Consequently, the patient population of the included studies resembled a continuum of primary to secondary care patients of which only some might plausibly resemble the primary care spectrum. Additionally primary care populations may be different in different countries, it would therefore be useful to identify those studies which are relevant to primary care irrespective of the actual study setting, for instance using tailored meta-analysis.[56, 57] Furthermore, the statistical validity of any summary estimates could be evaluated using a recently developed cross-validation technique.[58] Only five studies recruited subjects from primary care populations and analysed the general practitioners' decisions on whether to test and whether to refer.[35, 44, 47, 48, 53] We found lower sensitivity for detection of disease in these studies compared to meta-analyses of all studies.

For peer review only - http://bmjopen.bmj.com/site/about/guidelines.xhtml

Despite the publication of primary care studies since the Waugh et al. review,[18] we are still lacking evidence on the defined role of FC testing in the primary care pathway for the detection of IBD. This is a concern given that it is prescribed by local and national guidance in the UK and the roll out of a national algorithm endorsed by NICE, the NHS England Chief Scientific Officer and the British Society of Gastroenterologists is imminent to improve spread and adoption of FC testing nationally.[59] The decision to introduce FC testing in primary care might have been based on overoptimistic assessments of potential test accuracy in this setting. Evidence for the test accuracy of FC testing in primary care which considers the revised cut-off of 100μg/g is needed before any recommendations for use in primary care can be made. Potentially, test accuracy studies in primary care are hampered because verification of FC test outcomes with colonoscopy may not be feasible in all tested patients. Therefore, other reference standards for assessing the performance of FC testing in primary care are also needed.

For peer review only

For peer review only - http://bmjopen.bmj.com/site/about/guidelines.xhtml

References

1. Ungaro R, Mehandru S, Allen PB, Peyrin-Biroulet L, Colombel JF. Ulcerative colitis. *Lancet*. 2017;389(10080):1756-70.
2. Torres J, Mehandru S, Colombel JF, Peyrin-Biroulet L. Crohn's disease. *Lancet*. 2017;389(10080):1741-55.
3. Frolkis AD, Dykeman J, Negron ME, Debruyn J, Jette N, Fiest KM, *et al.* Risk of surgery for inflammatory bowel diseases has decreased over time: a systematic review and meta-analysis of population-based studies. *Gastroenterology*. 2013;145(5):996-1006.
4. Dahlhamer JM, Zammitti EP, Ward BW, Wheaton AG, Croft JB. Prevalence of Inflammatory Bowel Disease Among Adults Aged >/=18 Years - United States, 2015. *MMWR Morb Mortal Wkly Rep*. 2016;65(42):1166-9.
5. Burisch J, Jess T, Martinato M, Lakatos PL. The burden of inflammatory bowel disease in Europe. *J Crohns Colitis*. 2013;7(4):322-37.
6. Rubin GP, Hungin AP, Kelly PJ, Ling J. Inflammatory bowel disease: epidemiology and management in an English general practice population. *Aliment Pharmacol Ther*. 2000;14(12):1553-9.
7. Molodecky NA, Soon IS, Rabi DM, Ghali WA, Ferris M, Chernoff G, *et al.* Increasing incidence and prevalence of the inflammatory bowel diseases with time, based on systematic review. *Gastroenterology*. 2012;142(1):46-54.e42; quiz e30.
8. van den Heuvel-Janssen HAM, Borghouts JAJ, Muris JWM, Koes BW, Bouter LM, Knottnerus JA. Chronic non-specific abdominal complaints in general practice: a prospective study on management, patient health status and course of complaints. *BMC Family Practice*. 2006;7:12-.
9. Janssen HA, Borghouts JA, Muris JW, Metsemakers JF, Koes BW, Knottnerus JA. Health status and management of chronic non-specific abdominal complaints in general practice. *The British Journal of General Practice*. 2000;50(454):375-9.
10. Kok L, Elias SG, Witteman BJ, Goedhard JG, Muris JW, Moons KG, *et al.* Diagnostic accuracy of point-of-care fecal calprotectin and immunochemical occult blood tests for diagnosis of organic bowel disease in primary care: the Cost-Effectiveness of a Decision Rule for Abdominal Complaints in Primary Care (CEDAR) study. *Clinical Chemistry*. 2012;58(6):989-98.
11. Eskeland SL, Dalen E, Sponheim J, Lind E, Brunborg C, de Lange T. European panel on the appropriateness of gastrointestinal endoscopy II guidelines help in selecting and prioritizing patients referred to colonoscopy--a quality control study. *Scand J Gastroenterol*. 2014;49(4):492-500.
12. Konikoff MR, Denson LA. Role of fecal calprotectin as a biomarker of intestinal inflammation in inflammatory bowel disease. *Inflammatory Bowel Diseases*. 2006;12(6):524-34.
13. Lichtenstein GR, Loftus EV, Isaacs KL, Regueiro MD, Gerson LB, Sands BE. ACG Clinical Guideline: Management of Crohn's Disease in Adults. *The American Journal Of Gastroenterology*. 2018;113:481.
14. The Gastroenterological Society of Australia. Australian Guidelines for General Practitioners and Physicians: Inflammatory Bowel Disease, Forth Edition. 2017 [cited 08/10/2018]; Available from: http://cart.gesa.org.au/membes/files/Clinical%20Guidelines%20and%20Updates/2017_IBD_guidelines_DRAFT_for_consultation_20102017.pdf.
15. Preiß J, Bokemeyer B, Buhr H, Dignaß A, Häuser W, Hartmann F, *et al.* Updated German Clinical Practice Guideline on "Diagnosis and treatment of Crohn's disease". 2014 [cited 08/10/2018]; Available from: https://www.dgvs.de/wp-content/uploads/2016/11/DGVS_Empfehlung_fuer_Diagnistik_und_Therapie_des_Morbus_Crohn.pdf.
16. Gomollon F, Dignass A, Annese V, Tilg H, Van Assche G, Lindsay JO, *et al.* 3rd European Evidence-based Consensus on the Diagnosis and Management of Crohn's Disease 2016: Part 1: Diagnosis and Medical Management. *J Crohns Colitis*. 2017;11(1):3-25.

For peer review only - http://bmjopen.bmj.com/site/about/guidelines.xhtml

17. National Institute for Health and Care Excellence (NICE). Faecal calprotectin diagnostic tests for inflammatory diseases of the bowel: NICE diagnostics guidance [DG11]. 2013 [cited 17/12/2015]; Available from: https://www.nice.org.uk/guidance/dg11.
18. Waugh N, Cummins E, Royle P, Kandala NB, Shyangdan D, Arasaradnam R*, et al.* Faecal calprotectin testing for differentiating amongst inflammatory and non-inflammatory bowel diseases: systematic review and economic evaluation. *Health Technology Assessment (Winchester, England)*. 2013;17(55):xv-xix, 1-211.
19. Whiting PF, Rutjes AW, Westwood ME, Mallett S, Deeks JJ, Reitsma JB*, et al.* QUADAS-2: a revised tool for the quality assessment of diagnostic accuracy studies. *Ann Intern Med*. 2011;155(8):529-36.
20. R Core Team. R: A language and environment for statistical computing. R Foundation for Statistical Computing, Vienna, Austria. [computer program]. 2017. Available from: https://www.R-project.org/.
21. Chu H, Cole SR. Bivariate meta-analysis of sensitivity and specificity with sparse data: a generalized linear mixed model approach. *J Clin Epidemiol*. 2006;59(12):1331-2; author reply 2-3.
22. Partlett C, Takwoingi Y. Meta-analysis of test accuracy studies in R: a summary of user-written programs and step-by-step guide to using glmer. Version 1.0. 2016 30/01/2018 [cited 30/01/2018]; Available from: http://methods.cochrane.org/sdt/.
23. McInnes MDF, Moher D, Thombs BD, McGrath TA, Bossuyt PM, Clifford T*, et al.* Preferred Reporting Items for a Systematic Review and Meta-analysis of Diagnostic Test Accuracy Studies: The PRISMA-DTA Statement. *Jama*. 2018;319(4):388-96.
24. Oyaert M, Boel A, Jacobs J, Van den Bremt S, De Sloovere M, Vanpoucke H*, et al.* Analytical performance and diagnostic accuracy of six different faecal calprotectin assays in inflammatory bowel disease. *Clinical Chemistry & Laboratory Medicine*. 2017;21:21.
25. Labaere D, Smismans A, Van Olmen A, Christiaens P, D'Haens G, Moons V*, et al.* Comparison of six different calprotectin assays for the assessment of inflammatory bowel disease. *United European Gastroenterology Journal*. 2014;2(1):30-7.
26. Turvill J. High negative predictive value of a normal faecal calprotectin in patients with symptomatic intestinal disease. *Frontline Gastroenterology*. 2012;3(1):21-8.
27. Schoepfer AM, Trummler M, Seeholzer P, Seibold-Schmid B, Seibold F. Discriminating IBD from IBS: comparison of the test performance of fecal markers, blood leukocytes, CRP, and IBD antibodies. *Inflamm Bowel Dis*. 2008;14(1):32-9.
28. Otten CM, Kok L, Witteman BJ, Baumgarten R, Kampman E, Moons KG*, et al.* Diagnostic performance of rapid tests for detection of fecal calprotectin and lactoferrin and their ability to discriminate inflammatory from irritable bowel syndrome. *Clin Chem Lab Med*. 2008;46(9):1275-80.
29. Li XG, Lu YM, Gu F, Yang XL. [Fecal calprotectin in differential diagnosis of irritable bowel syndrome]. *Beijing Da Xue Xue Bao*. 2006;38(3):310-3.
30. El-Badry A, Sedrak H, Rashed L. Faecal calprotectin in differentiating between functional and organic bowel diseases. *Arab Journal of Gastroenterology*. 2010;11(2):70-3.
31. Burri E, Manz M, Rothen C, Rossi L, Beglinger C, Lehmann FS. Monoclonal antibody testing for fecal calprotectin is superior to polyclonal testing of fecal calprotectin and lactoferrin to identify organic intestinal disease in patients with abdominal discomfort. *Clin Chim Acta*. 2013;416:41-7.
32. Kennedy NA, Clark A, Walkden A, Chang JC, Fasci-Spurio F, Muscat M*, et al.* Clinical utility and diagnostic accuracy of faecal calprotectin for IBD at first presentation to gastroenterology services in adults aged 16-50 years. *Journal of Crohn's & colitis*. 2015;9(1):41-9.
33. Dhaliwal A, Zeino Z, Tomkins C, Cheung M, Nwokolo C, Smith S*, et al.* Utility of faecal calprotectin in inflammatory bowel disease (IBD): what cut-offs should we apply? *Frontline Gastroenterology*. 2015;6(1):14-9.

For peer review only - http://bmjopen.bmj.com/site/about/guidelines.xhtml

34. Damms A, Bischoff SC. Validation and clinical significance of a new calprotectin rapid test for the diagnosis of gastrointestinal diseases. *Int J Colorectal Dis*. 2008;23(10):985-92.
35. Pavlidis P, Chedgy FJ, Tibble JA. Diagnostic accuracy and clinical application of faecal calprotectin in adult patients presenting with gastrointestinal symptoms in primary care. *Scandinavian Journal of Gastroenterology*. 2013;48(9):1048-54.
36. Mowat C, Digby J, Strachan JA, Wilson R, Carey FA, Fraser CG*, et al.* Faecal haemoglobin and faecal calprotectin as indicators of bowel disease in patients presenting to primary care with bowel symptoms. *Gut*. 2016;65(9):1463-9.
37. De Sloovere MM, De Smet D, Baert FJ, Debrabandere J, Vanpoucke HJ. Analytical and diagnostic performance of two automated fecal calprotectin immunoassays for detection of inflammatory bowel disease. *Clinical Chemistry & Laboratory Medicine*. 2017;11:11.
38. Livzan MA, Lyalukova EA, Nechaeva G, Osipenko MF, Dolgih TI. [Fecal Noninvasive Tests (Calprotectin, Transferrin, Hemoglobin) in Complex Diagnosis of Diseases of Intestines]. *Eksperimental'Naia i Klinicheskaia Gastroenterologiia*. 2015(3):34-8.
39. Oyaert M, Trouve C, Baert F, De Smet D, Langlois M, Vanpoucke H. Comparison of two immunoassays for measurement of faecal calprotectin in detection of inflammatory bowel disease: (pre)-analytical and diagnostic performance characteristics. *Clinical Chemistry & Laboratory Medicine*. 2014;52(3):391-7.
40. Jang HW, Kim HS, Park SJ, Hong SP, Kim TI, Kim WH*, et al.* Accuracy of three different fecal calprotectin tests in the diagnosis of inflammatory bowel disease. *Intestinal Research*. 2016;14(4):305-13.
41. Sostres C, Lue A, Barra Pardos MV, Hijos G, Perales A, Puente JJ*, et al.* Combination of FOBT and fecal calprotectin may be useful for reducing unnecessary colonoscopies in symptomatic patients. *United European Gastroenterology Journal*. 2017;5 (5 Supplement 1):A779.
42. Alrubaiy L, Malik A, Rees I, Bowen D. Usefulness of fecal calprotectin in clinical practice in a district general hospital. *Inflammatory Bowel Diseases*. 2012;18:S53-S4.
43. Banerjee A, Srinivas M, Eyre R, Ellis R, Waugh N, Bardhan KD*, et al.* Faecal calprotectin for differentiating between irritable bowel syndrome and inflammatory bowel disease: A useful screen in daily gastroenterology practice. *Frontline Gastroenterology*. 2015;6(1):20-6.
44. Boyd A, Peplow C, Dhaliwal A, O'Rourke J, Davies J, Milestone A. Primary care faecal calprotectin pathway - A three year analysis. *Colorectal Disease*. 2016;18:27.
45. Carroccio A, Iacono G, Cottone M, Di Prima L, Cartabellotta F, Cavataio F*, et al.* Diagnostic accuracy of fecal calprotectin assay in distinguishing organic causes of chronic diarrhea from irritable bowel syndrome: a prospective study in adults and children. *Clin Chem*. 2003;49(6 Pt 1):861-7.
46. Caviglia GP, Pantaleoni S, Touscoz GA, Adriani A, Rosso C, Smedile A*, et al.* Fecal calprotectin is an effective diagnostic tool that differentiates inflammatory from functional intestinal disorders. *Scandinavian Journal of Gastroenterology*. 2014;49(12):1419-24.
47. Conroy S, Hale MF, Cross SS, Swallow K, Sidhu RH, Sargur R*, et al.* Unrestricted faecal calprotectin testing performs poorly in the diagnosis of inflammatory bowel disease in patients in primary care. *J Clin Pathol*. 2017.
48. Hogberg C, Karling P, Rutegard J, Lilja M. Diagnosing colorectal cancer and inflammatory bowel disease in primary care: The usefulness of tests for faecal haemoglobin, faecal calprotectin, anaemia and iron deficiency. A prospective study. *Scandinavian Journal of Gastroenterology*. 2017;52(1):69-75.
49. Tibble JA, Sigthorsson G, Foster R, Forgacs I, Bjarnason I. Use of surrogate markers of inflammation and Rome criteria to distinguish organic from nonorganic intestinal disease. *Gastroenterology*. 2002;123(2):450-60.

For peer review only - http://bmjopen.bmj.com/site/about/guidelines.xhtml

50. Tan M, Subramaniam Y, Cheong C, Hasan F, Moran GW, Fateen W, *et al.* The role of faecal calprotectin in a primary care referral pathway in the UK. *United European Gastroenterology Journal*. 2016;4 (5 Supplement 1):A443.
51. Limburg PJ, Ahlquist DA, Sandborn WJ, Mahoney DW, Devens ME, Harrington JJ, *et al.* Fecal calprotectin levels predict colorectal inflammation among patients with chronic diarrhea referred for colonoscopy. *Am J Gastroenterol*. 2000;95(10):2831-7.
52. Rosenfeld G, Greenup AJ, Round A, Takach O, Halparin L, Saadeddin A, *et al.* FOCUS: Future of fecal calprotectin utility study in inflammatory bowel disease. *World Journal of Gastroenterology*. 2016;22(36):8211-8.
53. Turvill J, O'Connell S, Brooks A, Bradley-Wood K, Laing J, Thiagarajan S, *et al.* Evaluation of a faecal calprotectin care pathway for use in primary care. *Primary Health Care Research & Development*. 2016;17(5):428-36.
54. Willis BH. Spectrum bias--why clinicians need to be cautious when applying diagnostic test studies. *Fam Pract*. 2008;25(5):390-6.
55. S.; N, K.; K, B. B. Faecal calprotectin-a good test used badly. *Colorectal Disease*. 2017;19(S4):64.
56. Willis BH, Hyde CJ. Estimating a test's accuracy using tailored meta-analysis-How setting-specific data may aid study selection. *J Clin Epidemiol*. 2014;67(5):538-46.
57. Willis BH, Hyde CJ. What is the test's accuracy in my practice population? Tailored meta-analysis provides a plausible estimate. *J Clin Epidemiol*. 2015;68(8):847-54.
58. Willis BH, Riley RD. Measuring the statistical validity of summary meta-analysis and meta-regression results for use in clinical practice. *Stat Med*. 2017;36(21):3283-301.
59. Commissioning PC. Faecal Calprotectin in Primary Care as a Decision Diagnostic for Inflammatory Bowel Disease and Irritable Bowel Syndrome. 2018 [cited 09/07/2018]; Available from: https://www.pcc-cic.org.uk/article/updated-guidance-help-gps-easily-diagnose-serious-bowel-conditions.

For peer review only

For peer review only - http://bmjopen.bmj.com/site/about/guidelines.xhtml

Figure legend

Figure 1 Indicative pathway of faecal calprotectin testing in primary care based on NICE guidelines (DG11) and expert opinion for adult patients presenting with chronic abdominal pain to primary care (IBS irritable bowel syndrome, TFT –thyroid function test, CRP C-reactive protein, FC faecal calprotectin)

Figure 2 Flow diagram of study inclusion

Figure 3 Overview of risk of bias and applicability concern of included studies

Figure 4 Forrest plot of A) Oyaert 2016[24] comparing 6 FC tests and B) Labaere 2014[25] comparing 5 FC tests at cut-off 50µg/g

Figure 5 ROC plot of sensitivity and specificity by test (filled shapes present summary estimates with 95% confidence region)

Figure 6 ROC plot of sensitivity and specificity by clinical question (filled shapes present summary estimates with 95% confidence region)

Figure 7 Pairs of sensitivity and specificity of 25,000 meta-analyses of 28 studies picking one outcome per study at random for each round of meta-analysis at a threshold of 50 µg/g. A) Receiver operating characteristic plot, B) 2D density plot scaled to illustrate spread and density (where the highest density represents a probability of 1,000/25,000)

For peer review only

For peer review only - http://bmjopen.bmj.com/site/about/guidelines.xhtml

Author contributions

KF and HF undertook the review. KF and BW carried out the analysis. KF, BW, STP and AC contributed to the interpretation of the findings. KF drafted the manuscript. All authors critically revised the manuscript and approved the final version. KF takes responsibility for the integrity and accuracy of the data analysis. KF acts as guarantor. The corresponding author attests that all listed authors meet authorship criteria and that no others meeting the criteria have been omitted.

Acknowledgement

We would like to thank the patient advisory group of the project ‘What is the role of faecal calprotectin testing in primary care” for their input.

Funding

This study was funded by the NIHR (NIHR Doctoral Research Fellowship, Mrs Karoline Freeman, DRF-2016-09-038). The views expressed are those of the author(s) and not necessarily those of the NHS, the NIHR or the Department of Health and Social Care. The funder had no role in the study design, data collection, data analysis and interpretation, writing of the report or the decision to submit for publication.

Conflict of interest

All authors have completed the ICMJE uniform disclosure form at www.icmje.org/coi_disclosure.pdf and declare: KF is funded by the NIHR through a doctoral research fellowship. STP and AC are supported by the NIHR CLAHRC West Midlands initiative. STP is funded by the NIHR through a career development fellowship. Brian Willis is funded by an MRC Clinician Scientist Fellowship award (MR/N007999/1). HF has nothing to disclose.

Ethical approval

Ethical approval not required

Data sharing statement

No additional data available

Transparency

For peer review only

For peer review only - http://bmjopen.bmj.com/site/about/guidelines.xhtml

The lead author and manuscript's guarantor (KF) affirms that the manuscript is an honest, accurate and transparent account of the study being reported; that no important aspects of the study have been omitted; and that any discrepancies from the study as planned have been explained.

For peer review only

For peer review only - http://bmjopen.bmj.com/site/about/guidelines.xhtml

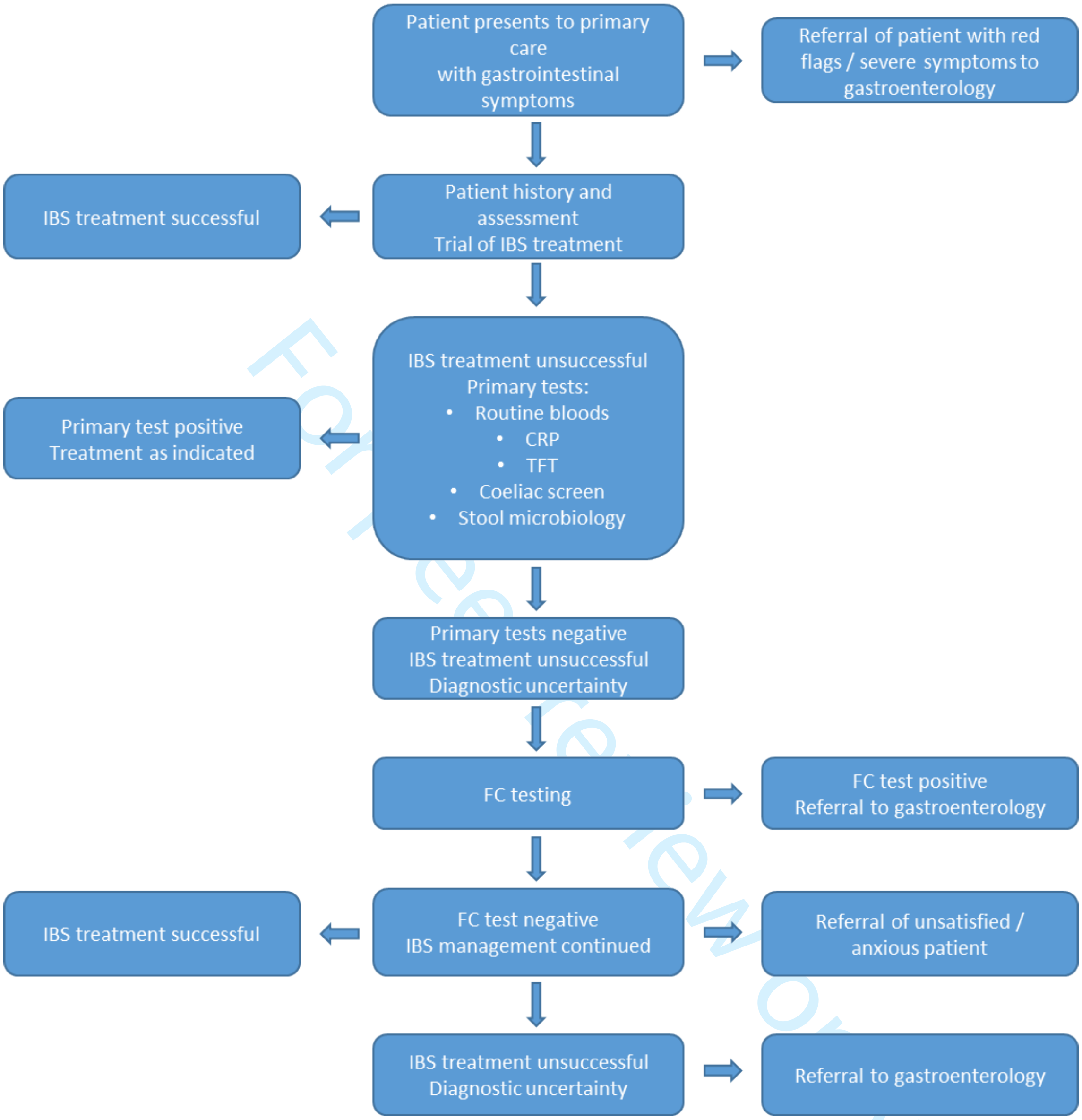

Figure 1 Indicative pathway of faecal calprotectin testing in primary care based on NICE guidelines (DG11) and expert opinion for adult patients presenting with chronic abdominal pain to primary care (IBS irritable bowel syndrome, TFT –thyroid function test, CRP C-reactive protein, FC faecal calprotectin)

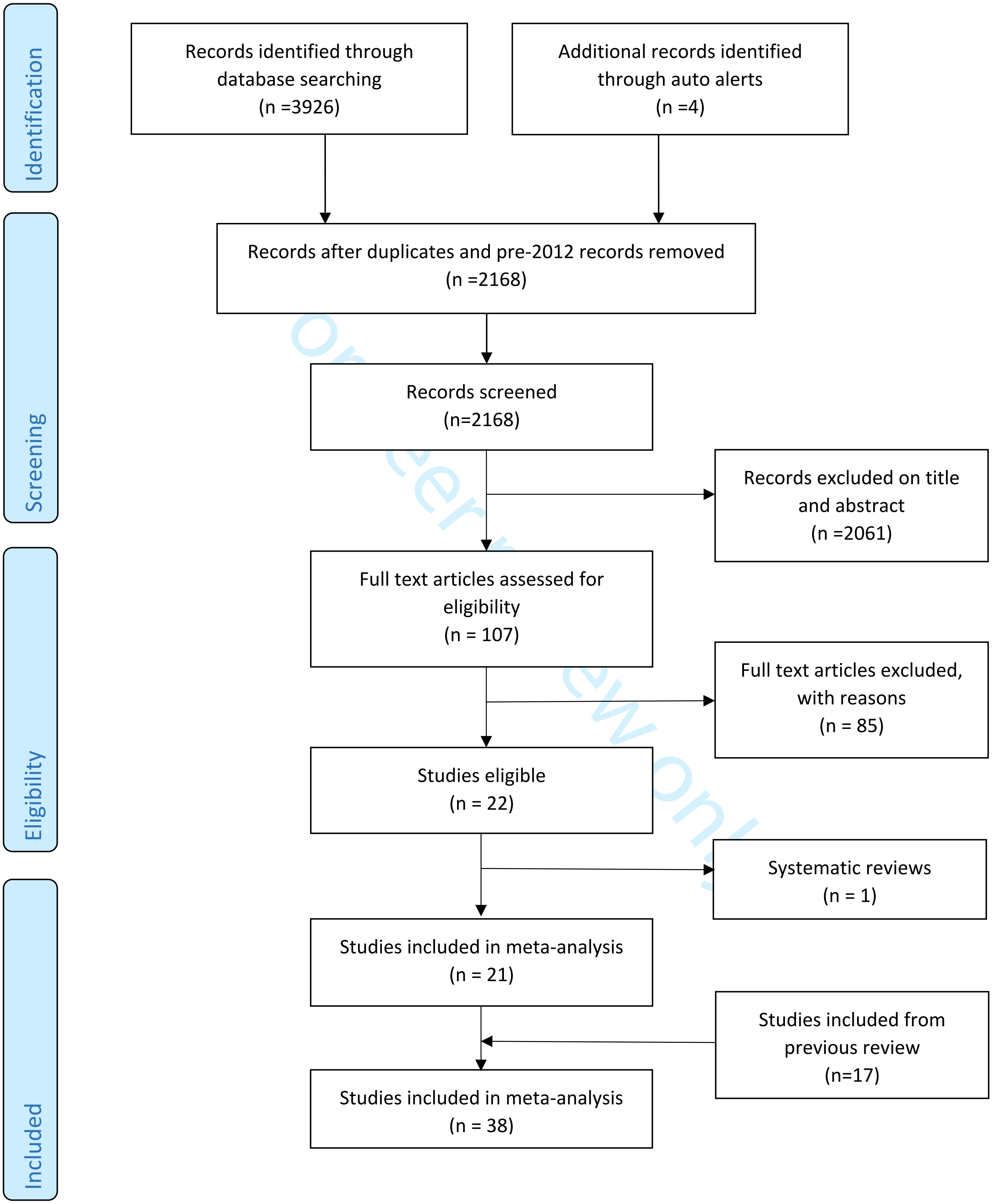
Identification
Screening
Eligibility
Included
Records identified through database searching (n =3926)
Additional records identified through auto alerts (n =4)
Records after duplicates and pre-2012 records removed (n =2168)
Records screened (n=2168)
Records excluded on title and abstract (n =2061)
Full text articles assessed for eligibility (n = 107)
Full text articles excluded, with reasons (n = 85)
Studies eligible (n = 22)
Systematic reviews (n = 1)
Studies included in meta-analysis (n = 21)
Studies included from previous review (n=17)
Studies included in meta-analysis (n = 38)

Figure 2 Flow diagram of study inclusion

For peer review only

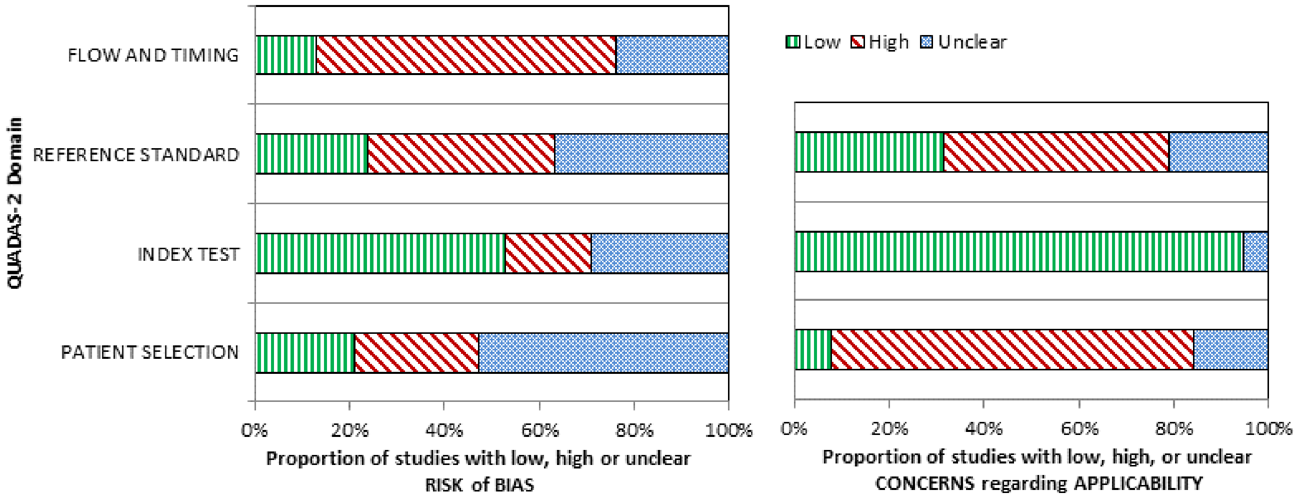

Figure 3 Overview of risk of bias and applicability concern of included studies according to QUADAS-2[15]

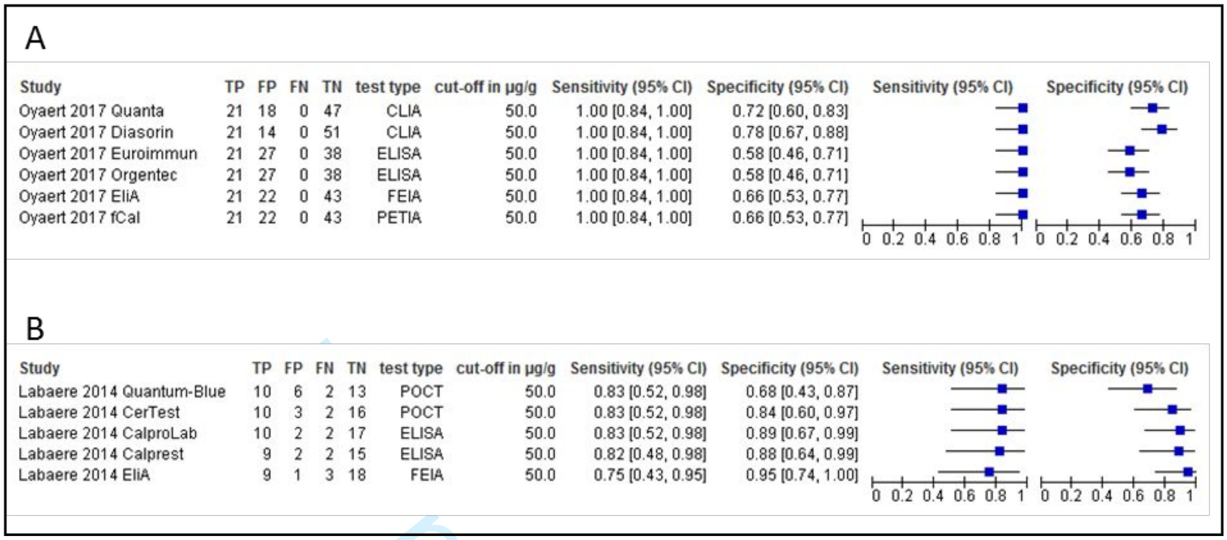

Figure 4 Forrest plot of A) Oyaert 2017[24] comparing 6 FC tests and B) Labaere 2014[25] comparing 5 FC tests at cut-off 50µg/g
(TP true positive, FP false positive, FC false negative, TN true negative, CI confidence interval, CLIA chemiluminescent immunoassay, ELISA - enzyme-linked immunosorbent assay, FEIA - fluorescence enzyme immunoassay, PETIA – particle enhanced turbimetric immunoassay, POCT point of care test)

For peer review only

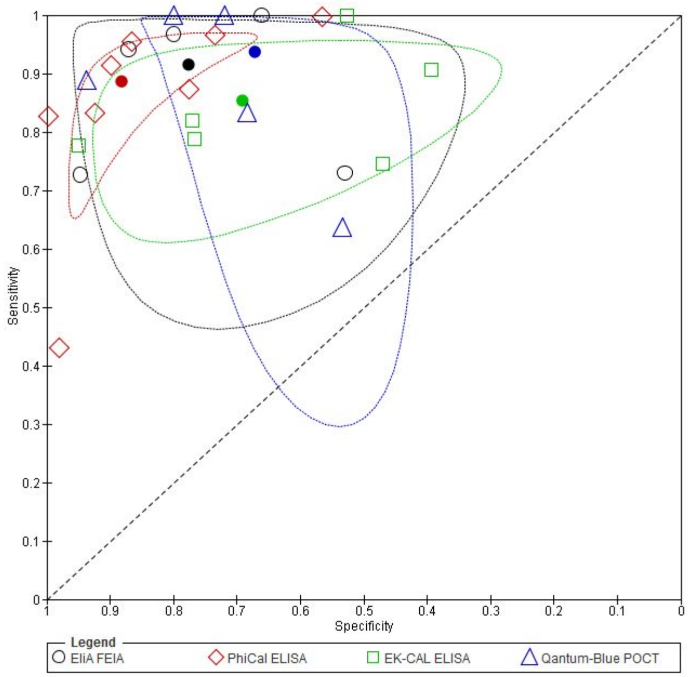

Figure 5 ROC plot of sensitivity and specificity by test (filled shapes present summary estimates with 95% confidence region)

For peer review only - http://bmjopen.bmj.com/site/about/guidelines.xhtml

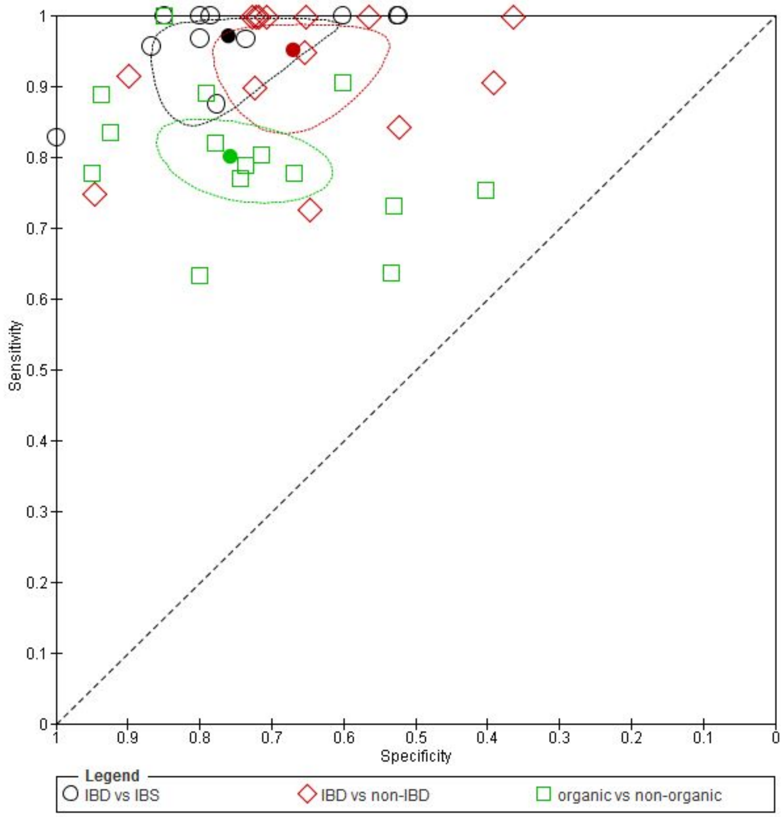

Figure 6 ROC plot of sensitivity and specificity by clinical question (filled shapes present summary estimates with 95% confidence region)

review only

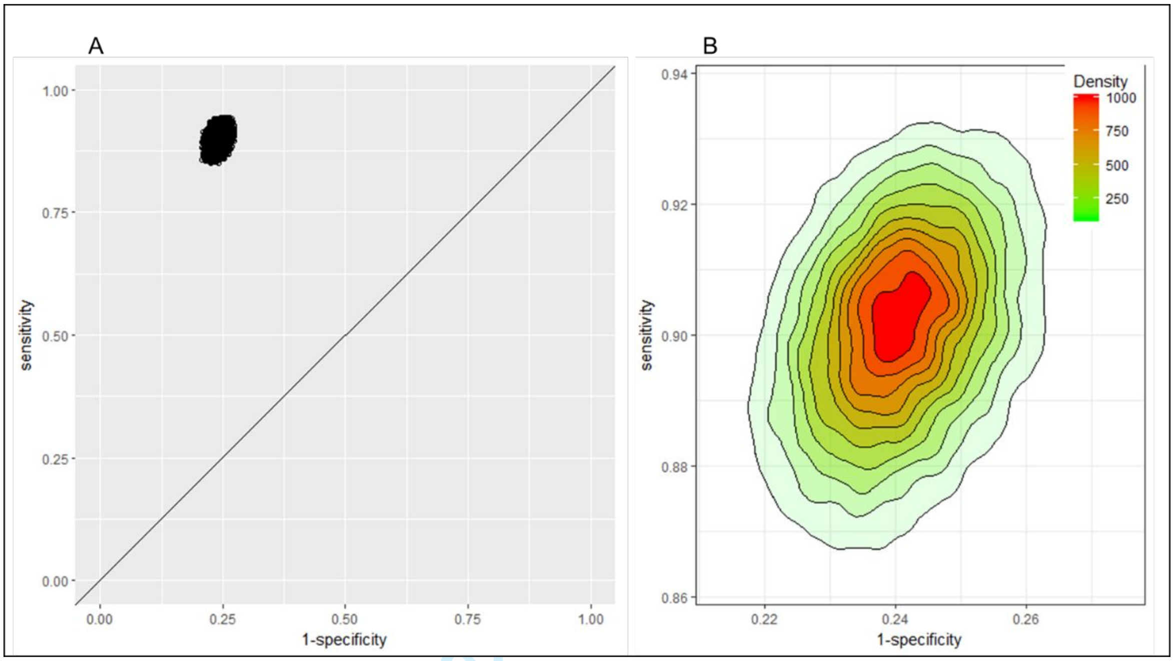

Figure 7 Pairs of sensitivity and specificity of 25,000 meta-analyses of 28 studies picking one outcome per study at random for each round of meta-analysis at a threshold of 50µg/g. A) Receiver operating characteristic plot, B) 2D density plot scaled to illustrate spread and density (where the highest density represents a probability of 1,000/25,000)

For peer review only

PRISMA-DTA Checklist

Section/topic	#	PRISMA-DTA Checklist Item	Reported on page #
**TITLE / ABSTRACT**			
Title	1	Identify the report as a systematic review (+/- meta-analysis) of diagnostic test accuracy (DTA) studies.	p1
Abstract	2	Abstract: See PRISMA-DTA for abstracts.	p2
**INTRODUCTION**			
Rationale	3	Describe the rationale for the review in the context of what is already known.	p4
Clinical role of index test	D1	State the scientific and clinical background, including the intended use and clinical role of the index test, and if applicable, the rationale for minimally acceptable test accuracy (or minimum difference in accuracy for comparative design).	p4
Objectives	4	Provide an explicit statement of question(s) being addressed in terms of participants, index test(s), and target condition(s).	p4
**METHODS**			
Protocol and registration	5	Indicate if a review protocol exists, if and where it can be accessed (e.g., Web address), and, if available, provide registration information including registration number.	p5
Eligibility criteria	6	Specify study characteristics (participants, setting, index test(s), reference standard(s), target condition(s), and study design) and report characteristics (e.g., years considered, language, publication status) used as criteria for eligibility, giving rationale.	p5
Information sources	7	Describe all information sources (e.g., databases with dates of coverage, contact with study authors to identify additional studies) in the search and date last searched.	p5
Search	8	Present full search strategies for all electronic databases and other sources searched, including any limits used, such that they could be repeated.	Suppl1
Study selection	9	State the process for selecting studies (i.e., screening, eligibility, included in systematic review, and, if applicable, included in the meta-analysis).	p5
Data collection process	10	Describe method of data extraction from reports (e.g., piloted forms, independently, in duplicate) and any processes for obtaining and confirming data from investigators.	p5
Definitions for data extraction	11	Provide definitions used in data extraction and classifications of target condition(s), index test(s), reference standard(s) and other characteristics (e.g. study design, clinical setting).	p5 and Suppl3
Risk of bias and applicability	12	Describe methods used for assessing risk of bias in individual studies and concerns regarding the applicability to the review question.	p5
Diagnostic accuracy measures	13	State the principal diagnostic accuracy measure(s) reported (e.g. sensitivity, specificity) and state the unit of assessment (e.g. per-patient, per-lesion).	p5
Synthesis of results	14	Describe methods of handling data, combining results of studies and describing variability between studies. This could include, but is not limited to: a) handling of multiple definitions of target condition. b) handling of multiple thresholds of test positivity, c) handling multiple index test readers, d) handling of indeterminate test results, e) grouping and comparing tests, f) handling of different reference standards	p6

For peer review only

PRISMA-DTA Checklist

Section/topic	#	PRISMA-DTA Checklist Item	Reported on page #
Meta-analysis	D2	Report the statistical methods used for meta-analyses, if performed.	p6
Additional analyses	16	Describe methods of additional analyses (e.g., sensitivity or subgroup analyses, meta-regression), if done, indicating which were pre-specified.	p6
**RESULTS**			
Study selection	17	Provide numbers of studies screened, assessed for eligibility, included in the review (and included in meta-analysis, if applicable) with reasons for exclusions at each stage, ideally with a flow diagram.	Figure2 and Suppl2
Study characteristics	18	For each included study provide citations and present key characteristics including: a) participant characteristics (presentation, prior testing), b) clinical setting, c) study design, d) target condition definition, e) index test, f) reference standard, g) sample size, h) funding sources	Table1 and Suppl4
Risk of bias and applicability	19	Present evaluation of risk of bias and concerns regarding applicability for each study.	Figure3 and Suppl5
Results of individual studies	20	For each analysis in each study (e.g. unique combination of index test, reference standard, and positivity threshold) report 2x2 data (TP, FP, FN, TN) with estimates of diagnostic accuracy and confidence intervals, ideally with a forest or receiver operator characteristic (ROC) plot.	Suppl6-9,11, Figures4-6, pp8-11
Synthesis of results	21	Describe test accuracy, including variability; if meta-analysis was done, include results and confidence intervals.	Table2, pp8-11
Additional analysis	23	Give results of additional analyses, if done (e.g., sensitivity or subgroup analyses, meta-regression; analysis of index test: failure rates, proportion of inconclusive results, adverse events).	Table2, p11, Figure7
**DISCUSSION**			
Summary of evidence	24	Summarize the main findings including the strength of evidence.	pp12-14
Limitations	25	Discuss limitations from included studies (e.g. risk of bias and concerns regarding applicability) and from the review process (e.g. incomplete retrieval of identified research).	p12
Conclusions	26	Provide a general interpretation of the results in the context of other evidence. Discuss implications for future research and clinical practice (e.g. the intended use and clinical role of the index test).	pp13-14
**FUNDING**			

For peer review only

PRISMA-DTA Checklist

Funding	27	For the systematic review, describe the sources of funding and other support and the role of the funders.	p20

Adapted From: McInnes MDF, Moher D, Thombs BD, McGrath TA, Bossuyt PM, The PRISMA-DTA Group (2018). Preferred Reporting Items for a Systematic Review and Meta-analysis of Diagnostic Test Accuracy Studies: The PRISMA-DTA Statement. JAMA. 2018 Jan 23;319(4):388-396. doi: 10.1001/jama.2017.19163.

For more information, visit: **www.prisma-statement.org**.

For peer review only

For peer review only - http://bmjopen.bmj.com/site/about/guidelines.xhtml

BMJ Open

Faecal calprotectin to detect inflammatory bowel disease - A systematic review and exploratory meta-analysis of test accuracy

Journal:	*BMJ Open*
Manuscript ID	bmjopen-2018-027428.R1
Article Type:	Research
Date Submitted by the Author:	09-Jan-2019
Complete List of Authors:	Freeman, Karoline; University of Warwick, Warwick Medical School<br>Willis, Brian; University of Birmingham, Institute of Applied Health Research<br>Fraser, Hannah; University of Warwick, Warwick Medical School<br>Taylor-Phillips, Sian; University of Warwick, Warwick Medical School<br>Clarke, Aileen; University of Warwick, Warwick Medical School
**Primary Subject Heading**:	Diagnostics
Secondary Subject Heading:	Gastroenterology and hepatology, Evidence based practice, General practice / Family practice
Keywords:	Inflammatory bowel disease < GASTROENTEROLOGY, PRIMARY CARE, Protocols & guidelines < HEALTH SERVICES ADMINISTRATION & MANAGEMENT

SCHOLARONE™
Manuscripts

Faecal calprotectin to detect inflammatory bowel disease - A systematic review and exploratory meta-analysis of test accuracy

Karoline Freeman, Brian H Willis, Hannah Fraser, Sian Taylor-Phillips, Aileen Clarke

Karoline Freeman NIHR Doctoral Fellow Division of Health Sciences, University of Warwick, Coventry, CV4 7AL, UK

Brian H Willis MRC Clinician Scientist Primary Care Clinical Sciences, Institute of Applied Health Research, University of Birmingham, Edgbaston, Birmingham, B15 2TT, UK

Hannah Fraser Research Associate Division of Health Sciences, University of Warwick, Coventry, CV4 7AL, UK

Sian Taylor-Phillips Associate Professor of Screening and Test Evaluation Division of Health Sciences, University of Warwick, Coventry, CV4 7AL, UK

Aileen Clarke Professor of Public Health & Health Services Research Division of Health Sciences, University of Warwick, Coventry, CV4 7AL, UK

Correspondence to:

Karoline Freeman, Division of Health Sciences, University of Warwick, Coventry, CV4 7AL, UK
Email: k.freeman@warwick.ac.uk

Word Count: 3385

For peer review only
For peer review only - http://bmjopen.bmj.com/site/about/guidelines.xhtml

ABSTRACT

Objective

Test accuracy of faecal calprotectin (FC) testing in primary care is inconclusive. We aimed to assess test accuracy of FC testing in primary care and compare it to secondary care estimates for the detection of inflammatory bowel disease (IBD).

Methods

Systematic review and meta-analysis of test accuracy using a bivariate random effects model. We searched MEDLINE, EMBASE, Cochrane Library and Web of Science until 31/05/2017 and included studies from auto alerts up until 31/01/2018. Eligible studies measured FC levels in stool samples to detect IBD in adult patients with chronic (at least 6-8 weeks) abdominal symptoms in primary or secondary care. Risk of bias and applicability were assessed using the Quality Assessment of Diagnostic Accuracy Studies-2 criteria. We followed the protocol registered as PROSPERO CRD 42012003287.

Results

38 out of 2168 studies were eligible including five from primary care. Comparison of test accuracy by setting was precluded by extensive heterogeneity. Overall summary estimates of sensitivity and specificity were not recorded. At a threshold of 50µg/g, sensitivity from separate meta-analysis of four assay types ranged from 0.85 (95% CI 0.75-0.92) to 0.94 (0.75-0.90) and specificity from 0.67 (0.56-0.76) to 0.88 (0.77-0.94). Across three different definitions of disease, sensitivity ranged from 0.80 (0.76-0.84) to 0.97 (0.91-0.99) and specificity from 0.67 (0.58-0.75) to 0.76 (0.66-0.84). Sensitivity appears to be lower in primary care and is further reduced at a revised threshold of 100µg/g.

Conclusions

Conclusive estimates of sensitivity and specificity of FC testing in primary care for the detection of IBD are still missing. There is insufficient evidence in the published literature to support the decision to introduce FC testing in primary care. Studies evaluating FC testing in an appropriate primary care setting are needed.

For peer review only - http://bmjopen.bmj.com/site/about/guidelines.xhtml

Strengths and limitations of this study

- This review used an innovative approach involving random combinations of test accuracy data from included studies in 25,000 meta-analyses to display the breadth of heterogeneous evidence from included studies.
- Exploratory meta-analyses investigated different test assays, clinical questions and positivity thresholds of faecal calprotectin levels.
- No overall summary estimates of sensitivity and specificity were produced due to heterogeneity.
- Comparison of test accuracy by setting was precluded by extensive heterogeneity in the small number of studies in primary care.
- The categorisation into different clinical questions was subjective because the disease categories are ill defined and studies' definitions of conditions and groups of conditions varied.

For peer review only

INTRODUCTION

Inflammatory bowel disease (IBD) is an organic disease caused by inflammation of the intestine. The disease is severe and progressive,[1, 2] and over 50% of people require surgery within 10 years of diagnosis.[3] Timely referral, which requires filtering of patients with a high probability of IBD from a broader group of patients with mainly irritable bowel syndrome (IBS), is vital. As many as 3.1 million people in the United States [4] and 2.5–3 million people in Europe [5] suffer from IBD with close to 300,000 patients in the UK.[6] An estimated 256,000 new cases of IBD are diagnosed annually throughout Europe.[5] Northern America and Northern Europe, including the UK, have the highest incidence rates.[7]

General practitioners refer between 10-20% of patients presenting with abdominal symptoms if no specific test is available.[8, 9] However, only about 25% of referred patients have organic disease, of which a third have IBD.[10, 11] Faecal calprotectin (FC), a regulator of the inflammatory response, could potentially aid more selective referral. Patients with high levels of FC have an increased probability of IBD. FC is a small calcium-binding protein. It is released into the intestinal lumen from activated neutrophils accumulating at the site of inflammation, and levels of FC are correlated with the level of inflammation.[12] The protein is stable at room temperature and resistant to digestion, making it a relatively easy candidate for a stool test for inflammation of the intestinal tract.

The FC test is FDA approved and recommended by gastroenterological societies across the globe for its usefulness in the diagnosis of IBD.[13-16] However, there is no clear guidance on which settings it is considered appropriate. FC testing is not supported by Medicare in the USA and Australia, but is approved for the differential diagnosis of IBD and IBS in adult patients in the UK by the National Institute for Health and Care Excellence (NICE) when referral to secondary care is being considered and cancer is not suspected (DG11).[17] At the time of the recommendations only test accuracy studies undertaken in secondary care were available to inform the decision.[18] Our knowledge on how the test performs in primary care is limited and a systematic approach to assessing the applicability of the available evidence specifically for this setting is timely. We aimed to assess the test accuracy of FC testing to detect IBD in adult patients with chronic abdominal symptoms in a primary care pathway (Figure 1). In addition we aimed to explore the differences in FC test performance between primary and secondary care.

++ Figure 1 ++

For peer review only - http://bmjopen.bmj.com/site/about/guidelines.xhtml

METHODS

Review methods

We followed the protocol by Waugh et.al.[18] registered as PROSPERO CRD 42012003287. We considered all studies included by Waugh et al.[18] for inclusion and searched MEDLINE, EMBASE, Cochrane Library and Web of Science from 01/09/2012 to 31/05/2017 (Supplement 1). Eligible studies from auto-alerts were included up until 31/01/2018. Reference lists of included studies were checked.

Studies were assessed for eligibility independently and in duplicate, and any disagreements were resolved by consensus. Records rejected at full text stage and reasons for exclusion were documented.

We included studies which measured FC levels in stool samples to detect IBD in adult patients (with ≥80% of study population 18-60 years) with chronic (at least 6-8 weeks) abdominal symptoms not yet diagnosed in primary or secondary care. The reference standard to verify FC test outcomes was colonoscopy with histology, other imaging technologies and follow-up. Our main outcomes of interest were sensitivity and specificity.

Data were extracted by KF on pre-specified data extraction sheets (Supplement 2) which were checked by HF. For studies reporting sensitivity, specificity, positive and negative predictive values and total number of included patients, 2x2 tables of true positives (TP), false positives (FP), false negatives (FN), and true negatives (TN) were calculated. For studies reporting test accuracy at a lower and an upper cut-off, 2x2 data tables were extracted for the lower and the upper cut-off. Authors of relevant studies with missing 2x2 data were contacted to request data.

Quality was assessed independently and in duplicate by two reviewers using the Quality Assessment of Diagnostic Accuracy Studies-2 criteria (QUADAS-2)[19] which included definitions for the signalling questions to match the review question.

Patient involvement

A patient advisory group was actively involved in the funding application for the project of which this study is a part. Two representatives of the patient advisory group will be involved in dissemination of findings at local and national events for IBD patients.

Analysis

For peer review only - http://bmjopen.bmj.com/site/about/guidelines.xhtml

Studies were considered under three main clinical questions with different definitions of the target and non-target conditions: FC testing to differentiate: 1) IBD from IBS, 2) IBD from non-IBD conditions and 3) organic from non-organic conditions. Different FC test assays were handled independently.

We used Review Manager 5.3 (The Cochrane Collaboration, The Nordic Cochrane Centre, Copenhagen, Denmark) to produce paired forest plots of sensitivity and specificity. All analyses were performed in R version 3.4.1(Vienna, Austria).[20] Meta-analyses were undertaken using a bivariate random effects model[21] using the package *glmer*[22] with a minimum of five studies required for meta-analysis. Different assays and clinical questions were considered separately. No overall summary estimates of sensitivity and specificity were produced due to heterogeneity. Test accuracy was explored at the commonly used thresholds of 50 and 100μg faecal calprotectin per g stool sample (μg/g). Outputs from meta-analyses were entered into Review Manager to produce receiver operating characteristic (ROC) plots.

Heterogeneity was explored by meta-regression analyses with assay type and clinical question added as covariates in turn. We tested the assumption of equal variances (Supplement 3) suggesting this was reasonable for the data set. Additional models assuming unequal variances did not converge.

Each meta-analysis considered only one outcome per study. However, a number of studies reported 2x2 data for multiple test assays and clinical questions. We therefore undertook an exploratory sensitivity analysis. First we produced a list of all possible combinations of test assay and clinical question for each included study at a common threshold (some studies reported outcomes for one test assay and clinical question contributing one possible combination while others reported results for up to three test assays and two clinical questions resulting in 6 possible combinations). We then ran a random sample of 25,000 meta-analyses out of a possible 10 million picking one outcome per study at random in each round of meta-analysis and considering all combinations of test assays and clinical questions reported in the studies. Pairs of sensitivity and specificity from these meta-analyses were plotted in an ROC plot and a 2D density plot to take account of the different definitions of disease and test assays and to allow visualisation of the variability in the data.

RESULTS

Figure 2 provides the PRISMA[23] flow diagram of study selection. Supplement 4 lists the records rejected at full text stage and reasons for exclusion. Out of 2168 unique records 38

For peer review only - http://bmjopen.bmj.com/site/about/guidelines.xhtml

studies were eligible for inclusion. The summary Table 1 reveals extensive heterogeneity in all major aspects of test accuracy studies. Study characteristics by study can be found in Supplement 5.

++ Figure 2 ++

Table 1 Summary of study characteristics of included studies addressing the test accuracy question of FC testing in the detection of IBD

Study characteristic	Outcome
**Study characteristics:**	
Publication type (studies)	
Full text	32
Abstract	6
Year of publication (range)	2000-2018
Population size (range)	31-1031
**Geographical region (studies):**	
UK	14
Rest of Europe	16
Asia	2
Middle East	2
North Africa	1
Russia	1
Canada	1
USA	1
**Patient characteristics:**	
Age (range)	14-97 years
IBD prevalence (range)	2.1% to 76%
**FC assay type (studies)*:**	
Immunoassays	
ELISA	32
FEIA	5
CLIA	3
PETIA	2
POCT	10
**Setting (studies)****	
Primary care	5
Secondary care	29
Out- and inpatients	12
Referred patients	17
Mix	2
**Target condition (studies)*:**	
IBD	23
Inflammatory disease	5
Organic disease	19
**Non-target condition (studies)*:**	
IBS	12
Functional disease	2
Non-IBD	12
Non-organic disease	16
Non-inflammatory disease	5
Other	3
**FC test data collection (studies):**	

For peer review only - http://bmjopen.bmj.com/site/about/guidelines.xhtml

Prospectively for patients with eligible symptoms in primary care	1
Prospectively for patients at time of referral in primary care	1
Retrospectively of routine FC tests in primary or secondary care	10
Prospectively prior to a planned colonoscopy in secondary care	23
Prospectively during the assessment for the need of colonoscopy in secondary care	1
Unclear	2
**Reference standard (studies):**	
Colonoscopy with biopsy	13
Colonoscopy +/- biopsy	7
Endoscopy + other imaging tests	8
Endoscopy + follow-up	3
Endoscopy + other imaging tests +follow-up	5
Unclear	2

CLIA - chemiluminescent immunoassay, PETIA – particle enhanced turbimetric immunoassay, ELISA - enzyme-linked immunosorbent assay (=EIA enzyme immunoassay), FEIA - fluorescence enzyme immunoassay, POCT – point of care test, IBD – inflammatory bowel disease
*some studies evaluated multiple tests/clinical questions
**Two studies were unclear about the setting

Figure 3 summarises the assessment of risk of bias and applicability (Supplement 6 for the assessment by study). Overall, the risk of bias was high or uncertain across all four domains in at least 50% of studies. Concerns about the applicability of the patient population to a primary care setting were high in about 75% of studies.

++ Figure 3 ++

The Forest plots in Supplement 7 describe the complete evidence on test accuracy from the 38 included studies reporting all the thresholds, assays and clinical questions explored. Overall, as might be expected, sensitivity decreases and specificity increases as the threshold increases. FC testing appears to be more accurate in the detection of the more precise clinical construct of IBD than in the detection of organic disease as a whole.

Two studies[24, 25] compared 5 and 6 different test assays at a common threshold of 50µg/g. This allows for a test comparison in the same study population under similar study conditions. It appears that the specificity varies to a greater extent than the sensitivity (Figure 4). Both studies reported medium to strong correlations but low agreement between assays, meaning that analysing the same sample with different FC test assays will result in different values for FC.

++ Figure 4 ++

Meta-analysis at 50µg/g threshold

For peer review only - http://bmjopen.bmj.com/site/about/guidelines.xhtml

28/38 studies reported test accuracy at the common 50µg/g cut-off and could be considered for meta-analysis. Meta-analyses were undertaken separately for test assays and for clinical questions to explore heterogeneity and to allow for multiple outcomes from a number of studies.

All 18 assays were considered in the meta-analysis investigating effect of assay type on summary estimates of test accuracy. (This was based on the assumption that the clinical question is generic and one outcome was picked per study for analysis). Four assays (PhiCal, EK-CAL, Quantum-Blue and EliA) with 5 or more studies each could be considered in the comparison (Supplement 8 for 2x2 data). 19 studies contributed data.[10, 24-41] Five studies [10, 25, 31, 39, 40] contributed data to two different assays. Figure 5 depicts the pairs of sensitivity and false positive rates of contributing studies in the ROC space, with summary estimates by assay type. At the global 50µg/g threshold, test performance appeared to vary slightly across assays (Table 2). Quantum-Blue had the highest summary estimate for sensitivity with 0.94 (95% CI 0.75-0.99) but also the lowest specificity (0.67, 95% CI 0.56-0.76). The greatest difference in sensitivity (9%) was between EK-CAL ELISA and Quantum-Blue POCT while the greatest difference in specificity (21%) was between PhiCal ELISA and Quantum-Blue POCT suggesting greater variation in specificity than in sensitivity across assays. Whether these differences are due to differing performance of test assays or due to methodological issues in study design is difficult to ascertain. The differences are large enough to suggest that test assays should not be treated as equivalent.

++ Figure 5 ++

Table 2 Comparison of results of separate meta-analyses and meta-regression with test type and clinical question as covariates at two thresholds (numbers in brackets are numbers of studies meta-analysed)

Test comparison at 50µg/g threshold								
**Method**	**PhiCal (n=8)**		**EK-CAL (n=6)**		**Quantum-Blue (n=5)**		**ELiA (n=5)**	
	**Sensitivity (95% CI)**	**Specificity (95% CI)**	**Sensitivity (95% CI)**	**Specificity (95% CI)**	**Sensitivity (95% CI)**	**Specificity (95% CI)**	**Sensitivity (95% CI)**	**Specificity (95% CI)**
**Separate MA for each test**	0.89 (0.76; 0.95)	0.88 (0.77; 0.94)	0.85 (0.75; 0.92)	0.69 (0.47; 0.85)	0.94 (0.75; 0.90)	0.67 (0.56; 0.76)	0.92 (0.78; 0.97)	0.78 (0.60; 0.89)
**Meta-regression (test) with equal variances**	0.89 (0.77; 0.95)	0.89 (0.79; 0.95)	0.87 (0.71; 0.95)	0.69 (0.49; 0.84)	0.92 (0.78; 0.98)	0.73 (0.50; 0.88)	0.92 (0.77; 0.97)	0.78 (0.57; 0.91)

Comparison of clinical questions at 50µg/g threshold						
**Method**	**IBD vs IBS (n=11)**		**IBD vs non-IBD (n=14)**		**Organic vs non-organic (n=15)**	
	**Sensitivity (95% CI)**	**Specificity (95% CI)**	**Sensitivity (95% CI)**	**Specificity (95% CI)**	**Sensitivity (95% CI)**	**Specificity (95% CI)**

For peer review only - http://bmjopen.bmj.com/site/about/guidelines.xhtml

**Separate MA for each clinical question**	0.97 (0.91; 0.99)	0.76 (0.66; 0.84)	0.95 (0.88; 0.98)	0.67 (0.58; 0.75)	0.80 (0.76; 0.84)	0.76 (0.66; 0.83)
**Meta-regression (clinical question) with equal variances**	0.96 (0.92; 0.98)	0.77 (0.66; 0.85)	0.94 (0.90; 0.97)	0.67 (0.57; 0.76)	0.81 (0.74; 0.86)	0.75 (0.67; 0.82)
**Comparison of clinical questions at 100µg/g threshold**						
**Method**	**IBD vs IBS (5)**		**IBD vs non-IBD (5)**		**Organic vs non-organic (7)**	
	**Sensitivity (95% CI)**	**Specificity (95% CI)**	**Sensitivity (95% CI)**	**Specificity (95% CI)**	**Sensitivity (95% CI)**	**Specificity (95% CI)**
**Separate MA for each clinical question**	0.92 (0.85; 0.96)	0.86 (0.82; 0.89)	0.72 (0.63; 0.80)	0.82 (0.78; 0.86)	0.67 (0.56; 0.76)	0.87 (0.84; 0.90)
**Meta-regression (clinical question) with equal variances**	0.92 (0.85; 0.96)	0.86 (0.81; 0.89)	0.73 (0.60; 0.82)	0.82 (0.77; 0.86)	0.67 (0.58; 0.75)	0.87 (0.84; 0.89)

(MA meta-analysis, CI confidence interval, IBD inflammatory bowel disease, IBS irritable bowel syndrome)

All reported 2x2 data (Supplement 9) for the clinical questions IBD versus IBS, IBD versus non-IBD and organic versus non-organic disease were considered in the meta-analysis investigating the effects of the different clinical questions on summary estimates of test accuracy under the assumption that the test is generic. 28 studies were included at the common threshold of 50µg/g.[10, 24-50] A total of 10 studies[30, 32, 34, 36, 37, 43, 45-48] contributed information to more than one category. The ROC plot in Figure 6 summarises the summary estimates for the comparison of the three clinical questions and the pairs of sensitivity and false positive rates for the contributing studies. Table 2 shows that specificity for IBD versus non-IBD (0.67, 95% CI 0.58-0.75) is lower than for IBD versus IBS (0.76, 95% CI 0.66-0.84), acknowledging that confidence intervals overlap, suggesting that broadening the definition of the non-target condition may produce more false positives as other non-IBD intestinal conditions are included. On the other hand, sensitivity for organic disease (0.80, 95% CI 0.76 to 0.84) is lower than for IBD (0.97, 95% CI 0.91 to 0.99) suggesting that some organic disease will be missed with FC testing as organic conditions, including adenomas and diverticulosis, are not typically associated with inflammation. Considering different definitions of the clinical questions results in greater variation in sensitivity than specificity meaning that widening the target condition had a greater impact on test accuracy than changing the non-target condition (IBS versus non-IBD versus non-organic disease).

For peer review only - http://bmjopen.bmj.com/site/about/guidelines.xhtml

++ Figure 6 ++

Meta-analysis at 100μg/g threshold

11 studies[30, 32, 35, 43, 45-48, 51-53] reported test accuracy at a threshold of 100μg/g (Supplement 10 for 2x2 data). Of these, five contributed to more than one category of clinical questions. As expected raising the threshold to 100μg/g reduces sensitivity and increases specificity for all clinical questions (Table 2). Insufficient numbers of studies at the 100μg/g were available to explore assay type.

Meta-regression and exploratory sensitivity analysis

Results from separate meta-analyses and from meta-regression analyses with equal variances showed similar results (Table 2 and Supplement 3).

Ten studies reported results for multiple clinical questions and eight studies compared multiple tests. Our approach of randomly selecting one test or one clinical question per study for meta-analysis uses only one set of evidence per study. Choosing a different set might have resulted in different outcomes and conclusions as the meta-analyses suggest that it might not be appropriate to consider tests and questions alike. In an attempt to capture this variation, Figure 7 displays results of 25,000 meta-analyses of 28 studies (Supplement 11 for 2x2 data) picking one outcome per study at random for each round of meta-analysis. The results show overall high sensitivity and specificity irrespective of test assay and clinical question (Panel A) with slightly greater variation in sensitivity than specificity (Panel B). Median sensitivity and specificity of the 25,000 analyses and ranges were 0.9 (min 0.85, max 0.94) and 0.76 (min 0.73, max 0.79), respectively.

++ Figure 7 ++

Primary versus secondary care

Five of the eligible studies were from primary care settings.[35, 44, 47, 48, 53] The studies were too heterogeneous for meta-analysis. In an attempt to compare test performance of FC testing in primary care with secondary care only one study qualified.[47] The study evaluated the routine use of FC testing in primary care and reported lower sensitivity of 0.72 but comparable specificity of 0.65 for differentiating IBD from non-IBD at a threshold of 50μg/g when compared to our pooled estimates across different test assays including all settings (sensitivity 0.95, CI 0.88-0.98 and specificity 0.67, CI 0.58-0.75). However, indication for testing and the place of the FC test in the patient pathway were unclear in this study and the study may suffer from differential verification bias due to concerns over the reference

For peer review only - http://bmjopen.bmj.com/site/about/guidelines.xhtml

standard. The authors explained the low sensitivity with the fact that general practitioners may have referred patients with high suspicion of IBD without testing. Another reasonable explanation could be the greater number of apparently milder IBD cases in primary care in which FC testing is falsely negative, reducing sensitivity in this setting, and demonstrating the impact of the different spectrum of disease on test accuracy estimates .[54]

DISCUSSION

None of the 38 included studies sufficiently addressed the review question of test accuracy of FC testing in a primary care pathway. The studies recruited patients with a different spectrum of disease from that anticipated for primary care, focussed on disease groups which were broader than the intended IBD group, or did not verify all patients with the preferred reference standard. Furthermore, the evidence was highly heterogeneous. Studies varied in thresholds and assays used, as well as in target conditions and settings. Eighteen different test assays were studied, although similar they could not be considered generic for meta-analysis. Clinical questions were analysed as three distinct categories which were related but which could not be pooled for meta-analysis. A number of studies compared multiple tests and / or explored different clinical questions. Picking one test and one clinical question per study for meta-analysis was unjustifiable. We favoured an innovative approach which allowed us to explore the whole breadth of evidence and showed that irrespective of clinical question and test assay, test accuracy of FC testing was high. However, the variation in specificity could translate into considerable uncertainty of false positives when scaled up to population testing, while the variation in sensitivity reflects the limited value of FC testing for other organic conditions.

The review has a number of limitations. Due to heterogeneity we decided not to pool across tests and clinical questions. Instead we conducted exploratory analyses which demonstrated that the test assay and clinical question may affect the summary sensitivity and specificity by as much as 9%. We were also unable to compare primary and secondary care studies due to the small number of heterogeneous studies in primary care. The meta-regression analyses assumed equal variances between categories of tests and clinical questions and this was supported by a statistical comparison. However, the power of this test is dependent on the number of studies per sub-group and it is possible that this assumption could be challenged with a larger data set. Finally, the categorisation into different clinical questions was subjective because the disease categories are ill defined and studies' definitions of conditions and groups of conditions varied.

For peer review only - http://bmjopen.bmj.com/site/about/guidelines.xhtml

This review is broadly in line with the approaches and interpretation of the evidence of the previous review.[18] However, Waugh et al. reported pooled estimates of sensitivity (0.93, 95% CI 0.83-0.97) and specificity (0.94, 95% CI 0.73-0.99) across tests for the differential diagnosis of IBD versus IBS for five secondary care studies and this was the basis for national decisions to introduce FC testing in primary care. When compared to our equivalent analysis of 11 studies we found comparable sensitivity (0.97, CI 0.91-0.99) but considerably lower specificity (0.76, CI 0.66-0.84) suggesting that false positives might be more of a concern than previously concluded. Thus the predicted reduction in colonoscopies and subsequent cost savings may not be realised as a result of introducing FC testing into the primary care pathway in the UK.[18] Furthermore, this review found enough disagreement between tests to caution against treating tests as equivalent. Issues in homogenisation, dilutions and extraction prior to analysis as well as lack of standardisation of different assays contribute to these differences. Recommended cut-off values would have to be determined locally until these issues are resolved.[24]

None of the primary care studies assessed FC testing for the differential diagnosis of IBD versus IBS. The comparison of IBD versus non-IBD might reflect the clinical situation for FC testing more accurately, but produces more false positives as FC levels might be raised in diverticular disease, coeliac disease, rectal adenocarcinoma, non-specific inflammation, and others.[35, 53] The meaning of the additional false positives due to non-IBD inflammatory conditions is debatable in the context of clinical practice where incidental findings of true disease would not be classed as a false positive test outcome but would prompt further investigations.

NICE recently endorsed a cut-off of 100µg/g for use in primary care in England and Wales, based on a primary care study which showed a 43% reduction in FC test positives compared with a threshold of 50µg/g.[47] We investigated test accuracy at this cut-off including all settings and demonstrated an increase in specificity by at least 10%, while the magnitude of the resulting decrease in sensitivity was more uncertain.

Referral of FC tested patients was consistent in three UK primary care studies (41%,[53] 42%[35] and 48%[47]) with over 25% of FC negative patients referred. This raises concerns about the impact of FC testing on colonoscopy rates as a considerable proportion of referred patients with negative FC levels were further investigated. Inappropriate use of FC testing as a screening test rather than to rule out IBD has been proposed as an explanation.[55] However, the number of investigations of referred FC negatives varied greatly among the three studies from 19%[47] to 46%[35] and even 71%[53] suggesting perhaps unsurprisingly, that

For peer review only - http://bmjopen.bmj.com/site/about/guidelines.xhtml

the number of potentially unnecessary colonoscopies may be at least as dependent on secondary care decision-making as on the availability of FC testing in primary care.

The applicability of the spectrum of secondary care patients to a primary care setting is questionable even if studies investigated referred patients. Firstly, general practitioners might have tested more patients than they referred and, secondly, patients referred came from different pathways including cancer pathways. Consequently, the patient population of the included studies resembled a continuum of primary to secondary care patients of which only some might plausibly resemble the primary care spectrum. Additionally primary care populations may be different in different countries, it would therefore be useful to identify those studies which are relevant to primary care irrespective of the actual study setting, for instance using tailored meta-analysis.[56, 57] Furthermore, the statistical validity of any summary estimates could be evaluated using a recently developed cross-validation technique.[58] Only five studies recruited subjects from primary care populations and analysed the general practitioners' decisions on whether to test and whether to refer.[35, 44, 47, 48, 53] We found lower sensitivity for detection of disease in these studies compared to meta-analyses of all studies.

Despite the publication of primary care studies since the Waugh et al. review,[18] we are still lacking evidence on the defined role of FC testing in the primary care pathway for the detection of IBD. This is a concern given that it is prescribed by local and national guidance in the UK and the roll out of a national algorithm endorsed by NICE, the NHS England Chief Scientific Officer and the British Society of Gastroenterologists is imminent to improve spread and adoption of FC testing nationally.[59] The decision to introduce FC testing in primary care might have been based on overoptimistic assessments of potential test accuracy in this setting. Evidence for the test accuracy of FC testing in primary care which considers the revised cut-off of 100μg/g is needed before any recommendations for use in primary care can be made. Potentially, test accuracy studies in primary care are hampered because verification of FC test outcomes with colonoscopy may not be feasible in all tested patients. Therefore, other reference standards for assessing the performance of FC testing in primary care are also needed.

For peer review only - http://bmjopen.bmj.com/site/about/guidelines.xhtml

References

1. Ungaro R, Mehandru S, Allen PB, Peyrin-Biroulet L, Colombel JF. Ulcerative colitis. *Lancet*. 2017;389(10080):1756-70.
2. Torres J, Mehandru S, Colombel JF, Peyrin-Biroulet L. Crohn's disease. *Lancet*. 2017;389(10080):1741-55.
3. Frolkis AD, Dykeman J, Negron ME, Debruyn J, Jette N, Fiest KM*, et al.* Risk of surgery for inflammatory bowel diseases has decreased over time: a systematic review and meta-analysis of population-based studies*. Gastroenterology*. 2013;145(5):996-1006.
4. Dahlhamer JM, Zammitti EP, Ward BW, Wheaton AG, Croft JB. Prevalence of Inflammatory Bowel Disease Among Adults Aged >/=18 Years - United States, 2015*. MMWR Morb Mortal Wkly Rep*. 2016;65(42):1166-9.
5. Burisch J, Jess T, Martinato M, Lakatos PL. The burden of inflammatory bowel disease in Europe*. J Crohns Colitis*. 2013;7(4):322-37.
6. Rubin GP, Hungin AP, Kelly PJ, Ling J. Inflammatory bowel disease: epidemiology and management in an English general practice population*. Aliment Pharmacol Ther*. 2000;14(12):1553-9.
7. Molodecky NA, Soon IS, Rabi DM, Ghali WA, Ferris M, Chernoff G*, et al.* Increasing incidence and prevalence of the inflammatory bowel diseases with time, based on systematic review*. Gastroenterology*. 2012;142(1):46-54.e42; quiz e30.
8. van den Heuvel-Janssen HAM, Borghouts JAJ, Muris JWM, Koes BW, Bouter LM, Knottnerus JA. Chronic non-specific abdominal complaints in general practice: a prospective study on management, patient health status and course of complaints*. BMC Family Practice*. 2006;7:12-.
9. Janssen HA, Borghouts JA, Muris JW, Metsemakers JF, Koes BW, Knottnerus JA. Health status and management of chronic non-specific abdominal complaints in general practice*. The British Journal of General Practice*. 2000;50(454):375-9.
10. Kok L, Elias SG, Witteman BJ, Goedhard JG, Muris JW, Moons KG*, et al.* Diagnostic accuracy of point-of-care fecal calprotectin and immunochemical occult blood tests for diagnosis of organic bowel disease in primary care: the Cost-Effectiveness of a Decision Rule for Abdominal Complaints in Primary Care (CEDAR) study*. Clinical Chemistry*. 2012;58(6):989-98.
11. Eskeland SL, Dalen E, Sponheim J, Lind E, Brunborg C, de Lange T. European panel on the appropriateness of gastrointestinal endoscopy II guidelines help in selecting and prioritizing patients referred to colonoscopy--a quality control study*. Scand J Gastroenterol*. 2014;49(4):492-500.
12. Konikoff MR, Denson LA. Role of fecal calprotectin as a biomarker of intestinal inflammation in inflammatory bowel disease*. Inflammatory Bowel Diseases*. 2006;12(6):524-34.
13. Lichtenstein GR, Loftus EV, Isaacs KL, Regueiro MD, Gerson LB, Sands BE. ACG Clinical Guideline: Management of Crohn's Disease in Adults*. The American Journal Of Gastroenterology*. 2018;113:481.
14. The Gastroenterological Society of Australia. Australian Guidelines for General Practitioners and Physicians: Inflammatory Bowel Disease, Forth Edition. 2017 [cited 08/10/2018]; Available from: http://cart.gesa.org.au/membes/files/Clinical%20Guidelines%20and%20Updates/2017_IBD_guidelines_DRAFT_for_consultation_20102017.pdf.
15. Preiß J, Bokemeyer B, Buhr H, Dignaß A, Häuser W, Hartmann F*, et al.* Updated German Clinical Practice Guideline on "Diagnosis and treatment of Crohn's disease". 2014 [cited 08/10/2018]; Available from: https://www.dgvs.de/wp-content/uploads/2016/11/DGVS_Empfehlung_fuer_Diagnistik_und_Therapie_des_Morbus_Crohn.pdf.
16. Gomollon F, Dignass A, Annese V, Tilg H, Van Assche G, Lindsay JO*, et al.* 3rd European Evidence-based Consensus on the Diagnosis and Management of Crohn's Disease 2016: Part 1: Diagnosis and Medical Management*. J Crohns Colitis*. 2017;11(1):3-25.

For peer review only - http://bmjopen.bmj.com/site/about/guidelines.xhtml

17. National Institute for Health and Care Excellence (NICE). Faecal calprotectin diagnostic tests for inflammatory diseases of the bowel: NICE diagnostics guidance [DG11]. 2013 [cited 17/12/2015]; Available from: https://www.nice.org.uk/guidance/dg11.
18. Waugh N, Cummins E, Royle P, Kandala NB, Shyangdan D, Arasaradnam R, *et al.* Faecal calprotectin testing for differentiating amongst inflammatory and non-inflammatory bowel diseases: systematic review and economic evaluation. *Health Technology Assessment (Winchester, England)*. 2013;17(55):xv-xix, 1-211.
19. Whiting PF, Rutjes AW, Westwood ME, Mallett S, Deeks JJ, Reitsma JB, *et al.* QUADAS-2: a revised tool for the quality assessment of diagnostic accuracy studies. *Ann Intern Med*. 2011;155(8):529-36.
20. R Core Team. R: A language and environment for statistical computing. R Foundation for Statistical Computing, Vienna, Austria. [computer program]. 2017. Available from: https://www.R-project.org/.
21. Chu H, Cole SR. Bivariate meta-analysis of sensitivity and specificity with sparse data: a generalized linear mixed model approach. *J Clin Epidemiol*. 2006;59(12):1331-2; author reply 2-3.
22. Partlett C, Takwoingi Y. Meta-analysis of test accuracy studies in R: a summary of user-written programs and step-by-step guide to using glmer. Version 1.0. 2016 30/01/2018 [cited 30/01/2018]; Available from: http://methods.cochrane.org/sdt/.
23. McInnes MDF, Moher D, Thombs BD, McGrath TA, Bossuyt PM, Clifford T, *et al.* Preferred Reporting Items for a Systematic Review and Meta-analysis of Diagnostic Test Accuracy Studies: The PRISMA-DTA Statement. *Jama*. 2018;319(4):388-96.
24. Oyaert M, Boel A, Jacobs J, Van den Bremt S, De Sloovere M, Vanpoucke H, *et al.* Analytical performance and diagnostic accuracy of six different faecal calprotectin assays in inflammatory bowel disease. *Clinical Chemistry & Laboratory Medicine*. 2017;21:21.
25. Labaere D, Smismans A, Van Olmen A, Christiaens P, D'Haens G, Moons V, *et al.* Comparison of six different calprotectin assays for the assessment of inflammatory bowel disease. *United European Gastroenterology Journal*. 2014;2(1):30-7.
26. Turvill J. High negative predictive value of a normal faecal calprotectin in patients with symptomatic intestinal disease. *Frontline Gastroenterology*. 2012;3(1):21-8.
27. Schoepfer AM, Trummler M, Seeholzer P, Seibold-Schmid B, Seibold F. Discriminating IBD from IBS: comparison of the test performance of fecal markers, blood leukocytes, CRP, and IBD antibodies. *Inflamm Bowel Dis*. 2008;14(1):32-9.
28. Otten CM, Kok L, Witteman BJ, Baumgarten R, Kampman E, Moons KG, *et al.* Diagnostic performance of rapid tests for detection of fecal calprotectin and lactoferrin and their ability to discriminate inflammatory from irritable bowel syndrome. *Clin Chem Lab Med*. 2008;46(9):1275-80.
29. Li XG, Lu YM, Gu F, Yang XL. [Fecal calprotectin in differential diagnosis of irritable bowel syndrome]. *Beijing Da Xue Xue Bao*. 2006;38(3):310-3.
30. El-Badry A, Sedrak H, Rashed L. Faecal calprotectin in differentiating between functional and organic bowel diseases. *Arab Journal of Gastroenterology*. 2010;11(2):70-3.
31. Burri E, Manz M, Rothen C, Rossi L, Beglinger C, Lehmann FS. Monoclonal antibody testing for fecal calprotectin is superior to polyclonal testing of fecal calprotectin and lactoferrin to identify organic intestinal disease in patients with abdominal discomfort. *Clin Chim Acta*. 2013;416:41-7.
32. Kennedy NA, Clark A, Walkden A, Chang JC, Fasci-Spurio F, Muscat M, *et al.* Clinical utility and diagnostic accuracy of faecal calprotectin for IBD at first presentation to gastroenterology services in adults aged 16-50 years. *Journal of Crohn's & colitis*. 2015;9(1):41-9.
33. Dhaliwal A, Zeino Z, Tomkins C, Cheung M, Nwokolo C, Smith S, *et al.* Utility of faecal calprotectin in inflammatory bowel disease (IBD): what cut-offs should we apply? *Frontline Gastroenterology*. 2015;6(1):14-9.
34. Damms A, Bischoff SC. Validation and clinical significance of a new calprotectin rapid test for the diagnosis of gastrointestinal diseases. *Int J Colorectal Dis*. 2008;23(10):985-92.

For peer review only - http://bmjopen.bmj.com/site/about/guidelines.xhtml

35. Pavlidis P, Chedgy FJ, Tibble JA. Diagnostic accuracy and clinical application of faecal calprotectin in adult patients presenting with gastrointestinal symptoms in primary care. *Scandinavian Journal of Gastroenterology*. 2013;48(9):1048-54.
36. Mowat C, Digby J, Strachan JA, Wilson R, Carey FA, Fraser CG, *et al.* Faecal haemoglobin and faecal calprotectin as indicators of bowel disease in patients presenting to primary care with bowel symptoms. *Gut*. 2016;65(9):1463-9.
37. De Sloovere MM, De Smet D, Baert FJ, Debrabandere J, Vanpoucke HJ. Analytical and diagnostic performance of two automated fecal calprotectin immunoassays for detection of inflammatory bowel disease. *Clinical Chemistry & Laboratory Medicine*. 2017;11:11.
38. Livzan MA, Lyalukova EA, Nechaeva G, Osipenko MF, Dolgih TI. [Fecal Noninvasive Tests (Calprotectin, Transferrin, Hemoglobin) in Complex Diagnosis of Diseases of Intestines]. *Eksperimental'Naia i Klinicheskaia Gastroenterologiia*. 2015(3):34-8.
39. Oyaert M, Trouve C, Baert F, De Smet D, Langlois M, Vanpoucke H. Comparison of two immunoassays for measurement of faecal calprotectin in detection of inflammatory bowel disease: (pre)-analytical and diagnostic performance characteristics. *Clinical Chemistry & Laboratory Medicine*. 2014;52(3):391-7.
40. Jang HW, Kim HS, Park SJ, Hong SP, Kim TI, Kim WH, *et al.* Accuracy of three different fecal calprotectin tests in the diagnosis of inflammatory bowel disease. *Intestinal Research*. 2016;14(4):305-13.
41. Sostres C, Lue A, Barra Pardos MV, Hijos G, Perales A, Puente JJ, *et al.* Combination of FOBT and fecal calprotectin may be useful for reducing unnecessary colonoscopies in symptomatic patients. *United European Gastroenterology Journal*. 2017;5 (5 Supplement 1):A779.
42. Alrubaiy L, Malik A, Rees I, Bowen D. Usefulness of fecal calprotectin in clinical practice in a district general hospital. *Inflammatory Bowel Diseases*. 2012;18:S53-S4.
43. Banerjee A, Srinivas M, Eyre R, Ellis R, Waugh N, Bardhan KD, *et al.* Faecal calprotectin for differentiating between irritable bowel syndrome and inflammatory bowel disease: A useful screen in daily gastroenterology practice. *Frontline Gastroenterology*. 2015;6(1):20-6.
44. Boyd A, Peplow C, Dhaliwal A, O'Rourke J, Davies J, Milestone A. Primary care faecal calprotectin pathway - A three year analysis. *Colorectal Disease*. 2016;18:27.
45. Carroccio A, Iacono G, Cottone M, Di Prima L, Cartabellotta F, Cavataio F, *et al.* Diagnostic accuracy of fecal calprotectin assay in distinguishing organic causes of chronic diarrhea from irritable bowel syndrome: a prospective study in adults and children. *Clin Chem*. 2003;49(6 Pt 1):861-7.
46. Caviglia GP, Pantaleoni S, Touscoz GA, Adriani A, Rosso C, Smedile A, *et al.* Fecal calprotectin is an effective diagnostic tool that differentiates inflammatory from functional intestinal disorders. *Scandinavian Journal of Gastroenterology*. 2014;49(12):1419-24.
47. Conroy S, Hale MF, Cross SS, Swallow K, Sidhu RH, Sargur R, *et al.* Unrestricted faecal calprotectin testing performs poorly in the diagnosis of inflammatory bowel disease in patients in primary care. *J Clin Pathol*. 2017.
48. Hogberg C, Karling P, Rutegard J, Lilja M. Diagnosing colorectal cancer and inflammatory bowel disease in primary care: The usefulness of tests for faecal haemoglobin, faecal calprotectin, anaemia and iron deficiency. A prospective study. *Scandinavian Journal of Gastroenterology*. 2017;52(1):69-75.
49. Tibble JA, Sigthorsson G, Foster R, Forgacs I, Bjarnason I. Use of surrogate markers of inflammation and Rome criteria to distinguish organic from nonorganic intestinal disease. *Gastroenterology*. 2002;123(2):450-60.
50. Tan M, Subramaniam Y, Cheong C, Hasan F, Moran GW, Fateen W, *et al.* The role of faecal calprotectin in a primary care referral pathway in the UK. *United European Gastroenterology Journal*. 2016;4 (5 Supplement 1):A443.
51. Limburg PJ, Ahlquist DA, Sandborn WJ, Mahoney DW, Devens ME, Harrington JJ, *et al.* Fecal calprotectin levels predict colorectal inflammation among patients with chronic diarrhea referred for colonoscopy. *Am J Gastroenterol*. 2000;95(10):2831-7.

For peer review only - http://bmjopen.bmj.com/site/about/guidelines.xhtml

52. Rosenfeld G, Greenup AJ, Round A, Takach O, Halparin L, Saadeddin A*, et al.* FOCUS: Future of fecal calprotectin utility study in inflammatory bowel disease. *World Journal of Gastroenterology*. 2016;22(36):8211-8.
53. Turvill J, O'Connell S, Brooks A, Bradley-Wood K, Laing J, Thiagarajan S*, et al.* Evaluation of a faecal calprotectin care pathway for use in primary care*. Primary Health Care Research & Development*. 2016;17(5):428-36.
54. Willis BH. Spectrum bias--why clinicians need to be cautious when applying diagnostic test studies*. Fam Pract*. 2008;25(5):390-6.
55. S.; N, K.; K, B. B. Faecal calprotectin-a good test used badly*. Colorectal Disease*. 2017;19(S4):64.
56. Willis BH, Hyde CJ. Estimating a test's accuracy using tailored meta-analysis-How setting-specific data may aid study selection*. J Clin Epidemiol*. 2014;67(5):538-46.
57. Willis BH, Hyde CJ. What is the test's accuracy in my practice population? Tailored meta-analysis provides a plausible estimate*. J Clin Epidemiol*. 2015;68(8):847-54.
58. Willis BH, Riley RD. Measuring the statistical validity of summary meta-analysis and meta-regression results for use in clinical practice*. Stat Med*. 2017;36(21):3283-301.
59. Commissioning PC. Faecal Calprotectin in Primary Care as a Decision Diagnostic for Inflammatory Bowel Disease and Irritable Bowel Syndrome. 2018 [cited 09/07/2018]; Available from: https://www.pcc-cic.org.uk/article/updated-guidance-help-gps-easily-diagnose-serious-bowel-conditions.

For peer review only

For peer review only - http://bmjopen.bmj.com/site/about/guidelines.xhtml

Figure legend

Figure 1 Indicative pathway of faecal calprotectin testing in primary care based on NICE guidelines (DG11) and expert opinion for adult patients presenting with chronic abdominal pain to primary care (IBS irritable bowel syndrome, TFT –thyroid function test, CRP C-reactive protein, FC faecal calprotectin)

Figure 2 Flow diagram of study inclusion

Figure 3 Overview of risk of bias and applicability concern of included studies according to QUADAS-2[19]

Figure 4 Forrest plot of A) Oyaert 2016[24] comparing 6 FC tests and B) Labaere 2014[25] comparing 5 FC tests at cut-off 50µg/g
(TP true positive, FP false positive, FC false negative, TN true negative, CI confidence interval, CLIA chemiluminescent immunoassay, ELISA - enzyme-linked immunosorbent assay, FEIA - fluorescence enzyme immunoassay, PETIA – particle enhanced turbimetric immunoassay, POCT point of care test)

Figure 5 Receiver operating characteristic (ROC) plot of sensitivity and specificity by test (filled shapes present summary estimates with 95% confidence region)

Figure 6 Receiver operating characteristic (ROC) plot of sensitivity and specificity by clinical question (filled shapes present summary estimates with 95% confidence region)

Figure 7 Pairs of sensitivity and specificity of 25,000 meta-analyses of 28 studies picking one outcome per study at random for each round of meta-analysis at a threshold of 50 µg/g. A) Receiver operating characteristic plot, B) 2D density plot scaled to illustrate spread and density (where the highest density represents a probability of 1,000/25,000)

For peer review only

Author contributions

KF and HF undertook the review. KF and BHW carried out the analysis. KF, BHW, STP and AC contributed to the interpretation of the findings. KF drafted the manuscript. All authors critically revised the manuscript and approved the final version. KF takes responsibility for the integrity and accuracy of the data analysis. KF acts as guarantor. The corresponding author attests that all listed authors meet authorship criteria and that no others meeting the criteria have been omitted.

Acknowledgement

We would like to thank the patient advisory group of the project 'What is the role of faecal calprotectin testing in primary care" for their input.

Funding

This study was funded by the NIHR (NIHR Doctoral Research Fellowship, Mrs Karoline Freeman, DRF-2016-09-038). The views expressed are those of the author(s) and not necessarily those of the NHS, the NIHR or the Department of Health and Social Care. The funder had no role in the study design, data collection, data analysis and interpretation, writing of the report or the decision to submit for publication.

Conflict of interest

All authors have completed the ICMJE uniform disclosure form at www.icmje.org/coi_disclosure.pdf and declare: KF is funded by the NIHR through a doctoral research fellowship. STP and AC are supported by the NIHR CLAHRC West Midlands initiative. STP is funded by the NIHR through a career development fellowship. BHW is funded by an MRC Clinician Scientist Fellowship award (MR/N007999/1). HF has nothing to disclose.

Ethical approval

Ethical approval not required

Data sharing statement

All data relevant to the study are included in the article or uploaded as supplementary information

Transparency

The lead author and manuscript's guarantor (KF) affirms that the manuscript is an honest, accurate and transparent account of the study being reported; that no important aspects of

For peer review only - http://bmjopen.bmj.com/site/about/guidelines.xhtml

the study have been omitted; and that any discrepancies from the study as planned have been explained.

For peer review only

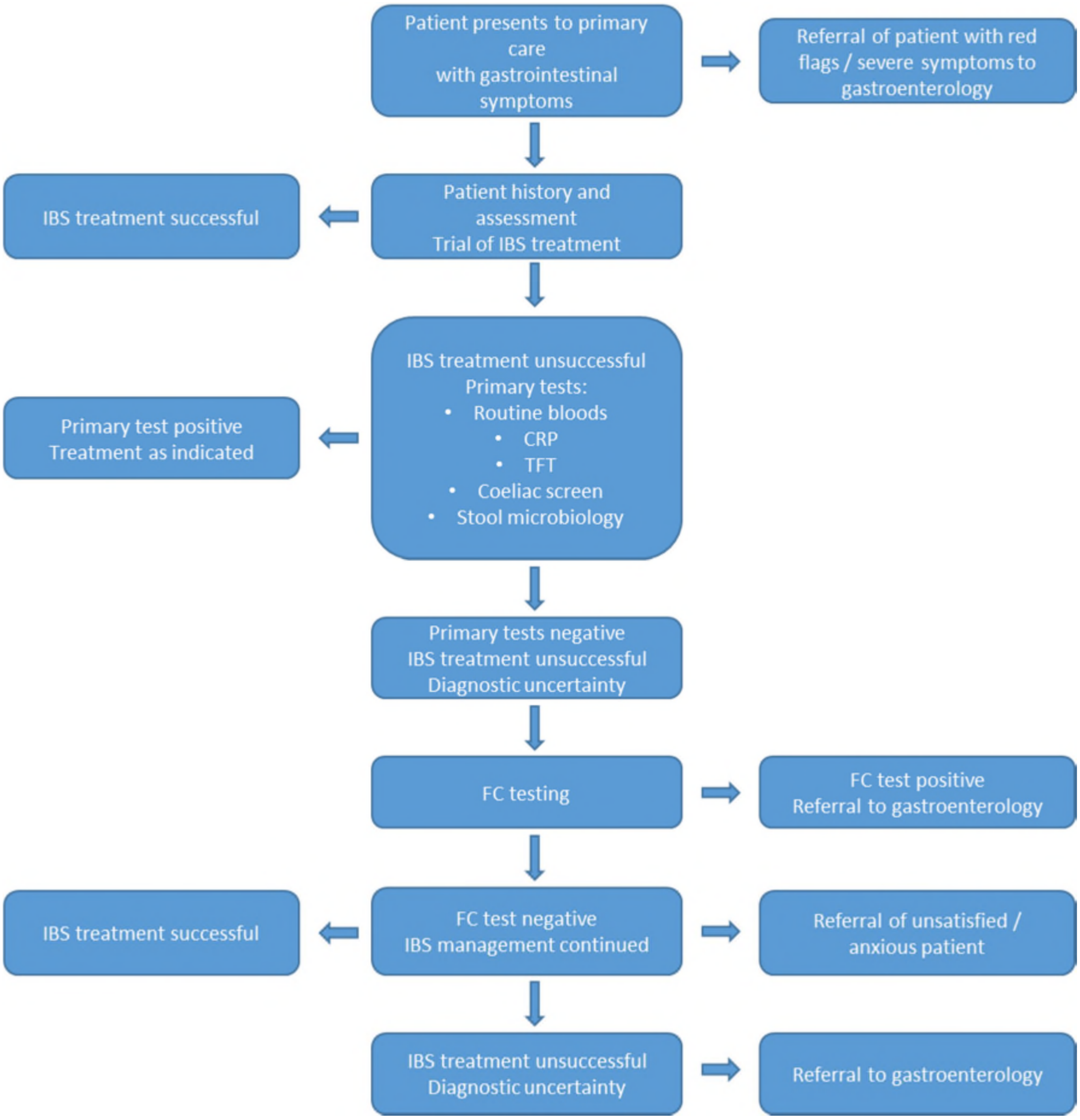
Patient presents to primary care with gastrointestinal symptoms
Referral of patient with red flags / severe symptoms to gastroenterology
IBS treatment successful
Patient history and assessment Trial of IBS treatment
Primary test positive Treatment as indicated
IBS treatment unsuccessful Primary tests:
• Routine bloods
• CRP
• TFT
• Coeliac screen
• Stool microbiology
Primary tests negative IBS treatment unsuccessful Diagnostic uncertainty
FC testing
FC test positive Referral to gastroenterology
IBS treatment successful
FC test negative IBS management continued
Referral of unsatisfied / anxious patient
IBS treatment unsuccessful Diagnostic uncertainty
Referral to gastroenterology

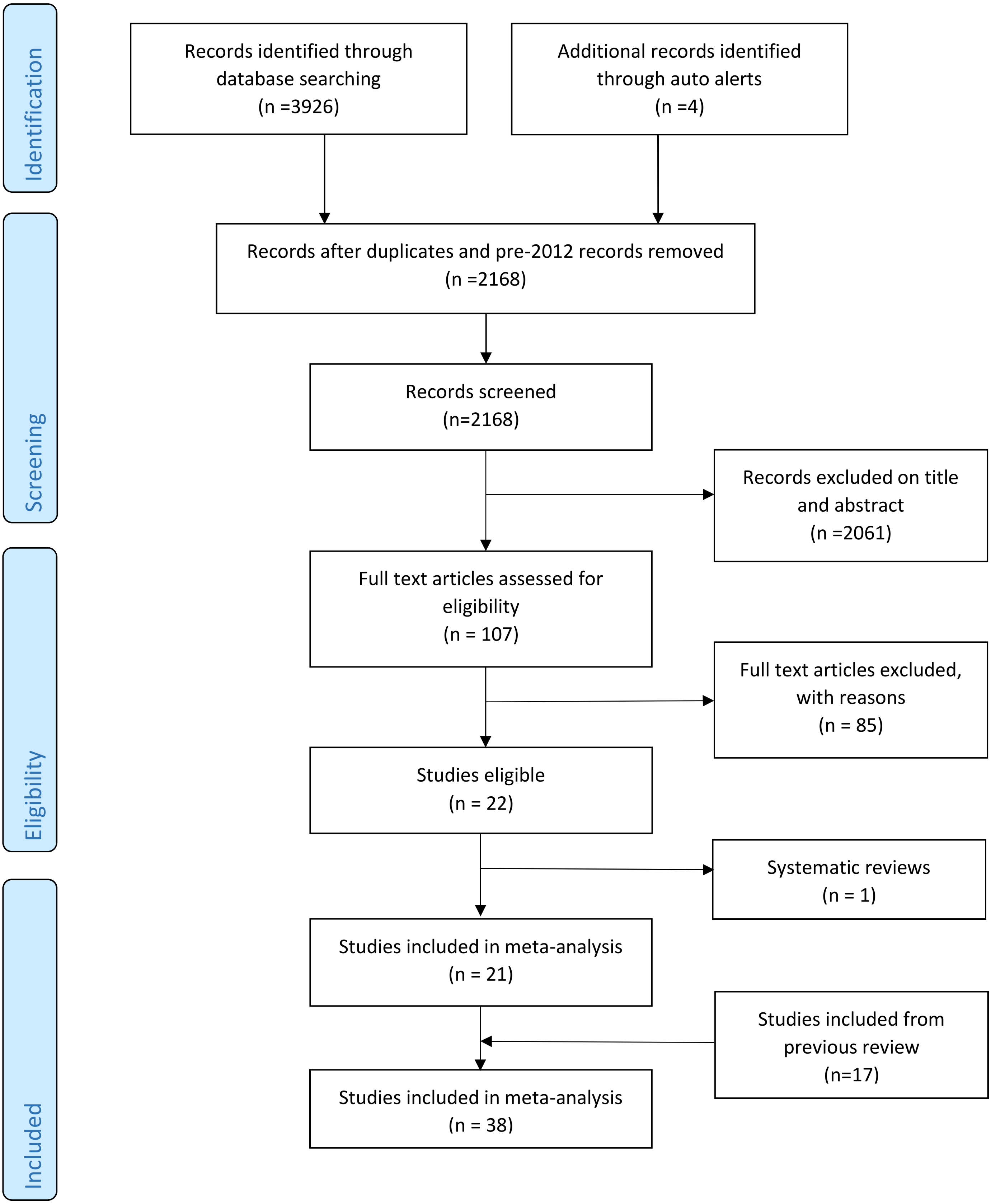
Identification
Screening
Eligibility
Included
Records identified through database searching (n =3926)
Additional records identified through auto alerts (n =4)
Records after duplicates and pre-2012 records removed (n =2168)
Records screened (n=2168)
Records excluded on title and abstract (n =2061)
Full text articles assessed for eligibility (n = 107)
Full text articles excluded, with reasons (n = 85)
Studies eligible (n = 22)
Systematic reviews (n = 1)
Studies included in meta-analysis (n = 21)
Studies included from previous review (n=17)
Studies included in meta-analysis (n = 38)

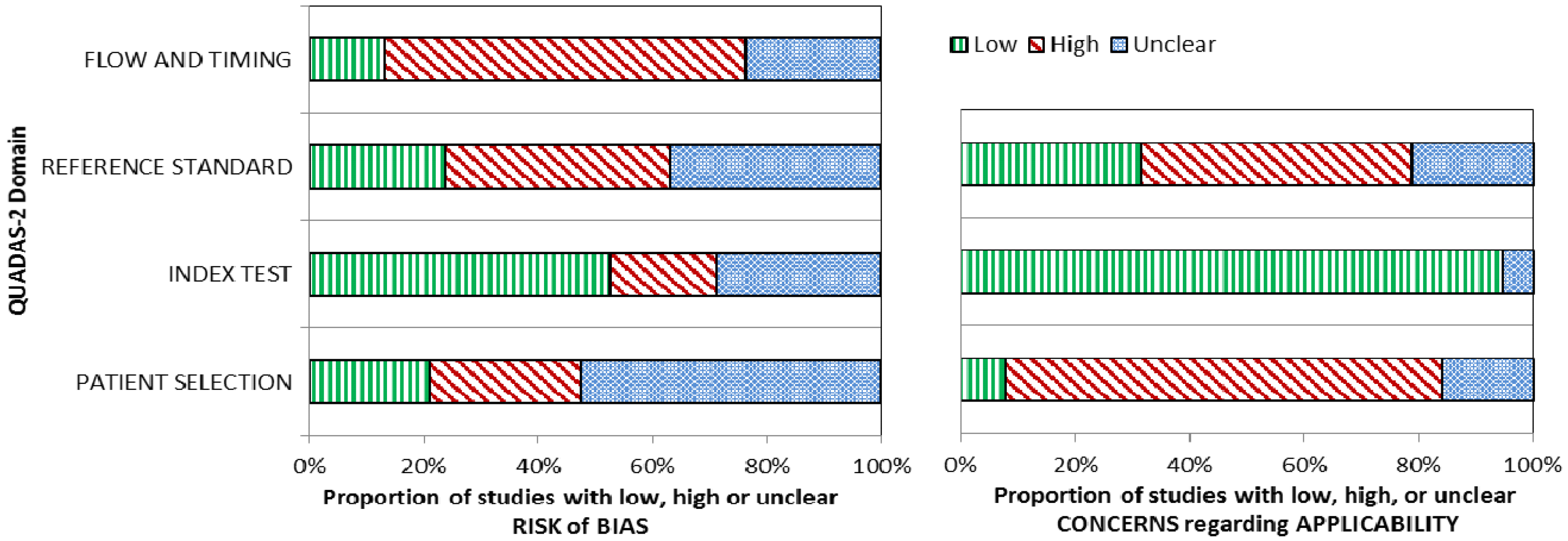
QUADAS-2 Domain
FLOW AND TIMING
REFERENCE STANDARD
INDEX TEST
PATIENT SELECTION
0%
20%
40%
60%
80%
100%
Proportion of studies with low, high or unclear RISK of BIAS
Low
High
Unclear
Proportion of studies with low, high, or unclear CONCERNS regarding APPLICABILITY

A

Study	TP	FP	FN	TN	test type	cut-off in µg/g	Sensitivity (95% CI)	Specificity (95% CI)
Oyaert 2017 Diasorin	21	14	0	51	CLIA	50.0	1.00 [0.84, 1.00]	0.78 [0.67, 0.88]
Oyaert 2017 Quanta	21	18	0	47	CLIA	50.0	1.00 [0.84, 1.00]	0.72 [0.60, 0.83]
Oyaert 2017 Orgentec	21	27	0	38	ELISA	50.0	1.00 [0.84, 1.00]	0.58 [0.46, 0.71]
Oyaert 2017 Euroimmun	21	27	0	38	ELISA	50.0	1.00 [0.84, 1.00]	0.58 [0.46, 0.71]
Oyaert 2017 EliA	21	22	0	43	FEIA	50.0	1.00 [0.84, 1.00]	0.66 [0.53, 0.77]
Oyaert 2017 fCal	21	22	0	43	PETIA	50.0	1.00 [0.84, 1.00]	0.66 [0.53, 0.77]

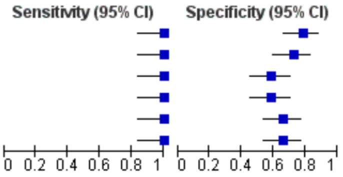

B

Study	TP	FP	FN	TN	test type	cut-off in µg/g	Sensitivity (95% CI)	Specificity (95% CI)
Labaere 2014 CerTest	10	3	2	16	POCT	50.0	0.83 [0.52, 0.98]	0.84 [0.60, 0.97]
Labaere 2014 Quantum-Blue	10	6	2	13	POCT	50.0	0.83 [0.52, 0.98]	0.68 [0.43, 0.87]
Labaere 2014 CalproLab	10	2	2	17	ELISA	50.0	0.83 [0.52, 0.98]	0.89 [0.67, 0.99]
Labaere 2014 Calprest	9	2	2	15	ELISA	50.0	0.82 [0.48, 0.98]	0.88 [0.64, 0.99]
Labaere 2014 EliA	9	1	3	18	FEIA	50.0	0.75 [0.43, 0.95]	0.95 [0.74, 1.00]

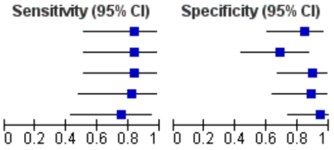

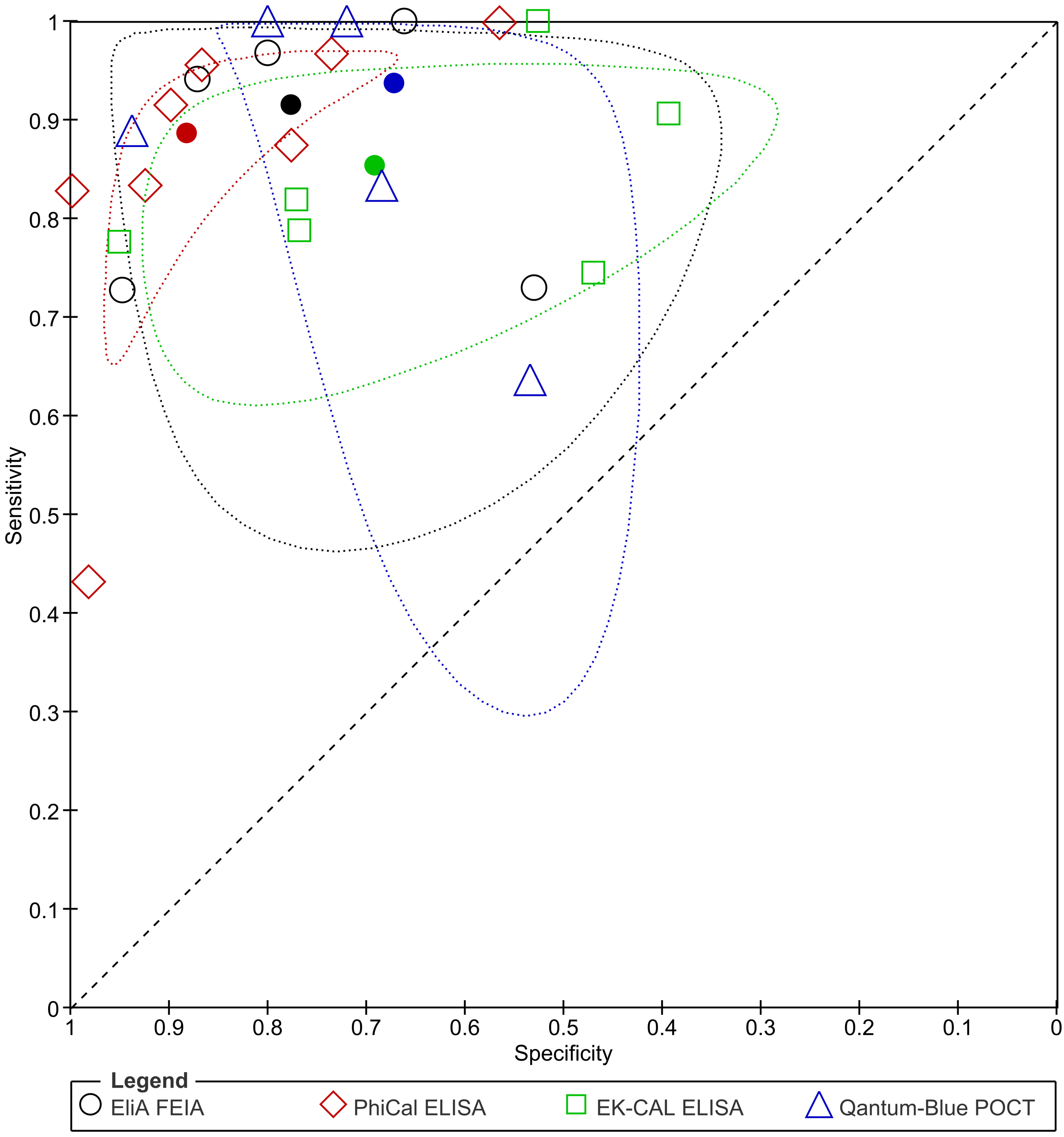
Sensitivity
Specificity
1
0.9
0.8
0.7
0.6
0.5
0.4
0.3
0.2
0.1
0
Legend
EliA FEIA
PhiCal ELISA
EK-CAL ELISA
Qantum-Blue POCT

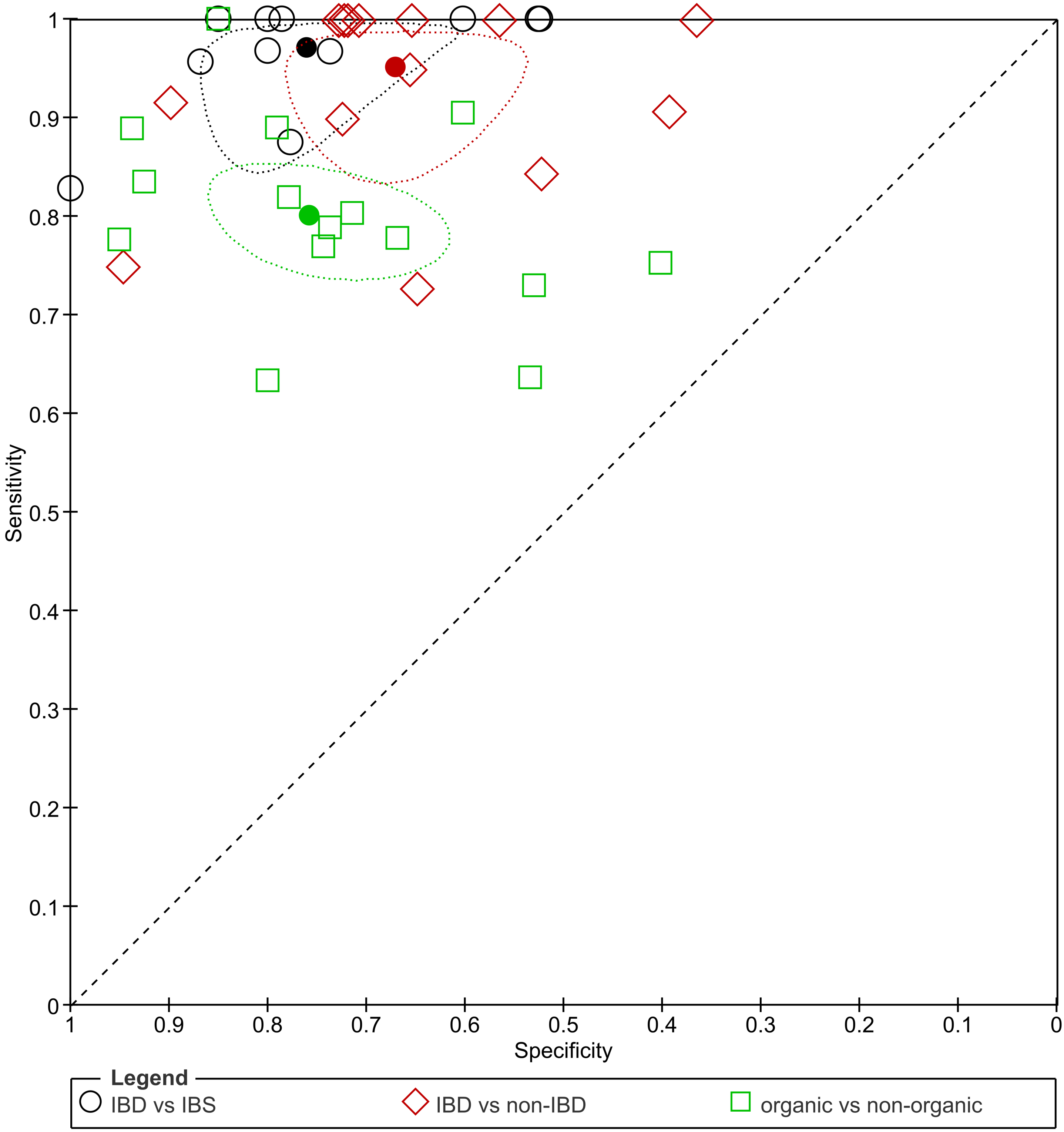
Sensitivity
1
0.9
0.8
0.7
0.6
0.5
0.4
0.3
0.2
0.1
0
Specificity
1
0.9
0.8
0.7
0.6
0.5
0.4
0.3
0.2
0.1
0
Legend
IBD vs IBS
IBD vs non-IBD
organic vs non-organic

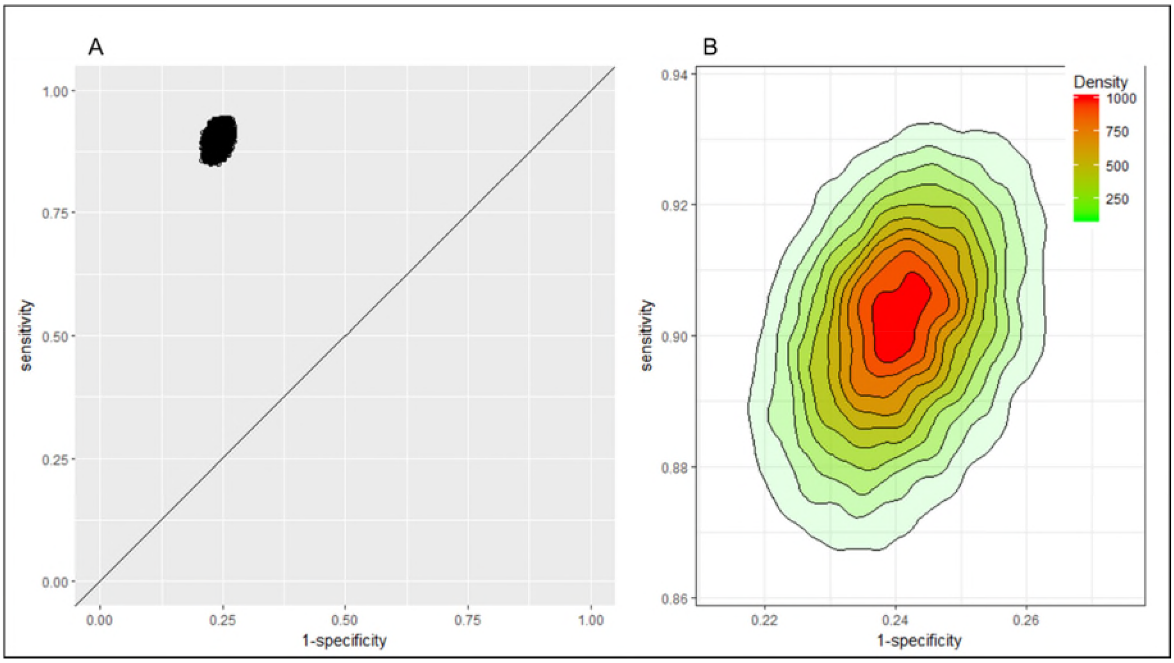
A
B
sensitivity
1-specificity
1.00
0.75
0.50
0.25
0.00
0.94
0.92
0.90
0.88
0.86
0.22
0.24
0.26
Density
1000
750
500
250

Supplement

Supplement 1 Search strategy

Ovid MEDLINE(R) 1946 to May Week 3 2017 (searched on 31/05/2017)
472 references exported

1. exp Inflammatory Bowel Diseases/di [Diagnosis]	10670
2. exp Irritable Bowel Syndrome/di [Diagnosis]	1303
3. Crohn* disease.tw.	34506
4. Ulcerative colitis.tw.	29458
5. Inflammatory bowel disease.tw.	27920
6. Irritable bowel syndrome.tw.	9092
7. (IBS or IBD).tw.	20353
8. (chronic diarrhoea or chronic diarrhea).tw.	3608
9. (abdominal pain or abdominal discomfort).tw.	42241
10. 1 or 2 or 3 or 4 or 5 or 6 or 7 or 8 or 9	124837
11. Calprotectin.tw.	1463
12. exp Leukocyte L1 Antigen Complex/an [Analysis]	606
13. 11 or 12	1594
14. 10 and 13	752
15. Limit 14 to ed=20120901-20170531	472

Ovid MEDLINE(R) Daily Update May 30, 2017, Ovid MEDLINE(R) In-Process & Other Non-Indexed Citations May 30, 2017, Ovid MEDLINE(R) Epub Ahead of Print May 30, 2017 (searched on 31/05/2017)
240 references exported

1. exp Inflammatory Bowel Diseases/di [Diagnosis]	15
2. exp Irritable Bowel Syndrome/di [Diagnosis]	3
3. Crohn* disease.tw.	4163
4. Ulcerative colitis.tw.	3292
5. Inflammatory bowel disease.tw.	4537
6. Irritable bowel syndrome.tw.	1568
7. (IBS or IBD).tw.	4167
8. (chronic diarrhoea or chronic diarrhea).tw.	332
9. (abdominal pain or abdominal discomfort).tw.	7253
10. 1 or 2 or 3 or 4 or 5 or 6 or 7 or 8 or 9	17922
11. Calprotectin.tw.	435
12. exp Leukocyte L1 Antigen Complex/an [Analysis]	3
13. 11 or 12	435
14. 10 and 13	240

Embase 1974 to 2017 May 30 (searched on 31/05/2017)
1995 references exported

1. crohn* disease.tw.	58919
2. ulcerative colitis.tw.	47316
3. inflammatory bowel disease*.tw.	56906
4. irritable bowel syndrome*.tw.	16123
5. (IBD or IBS).tw.	45994
6. (chronic diarrhoea or chronic diarrhea).tw.	5437
7. (abdominal pain or abdominal discomfort).tw.	77520
8. 1 or 2 or 3 or 4 or 5 or 6 or 7	213302
9. calprotectin.tw.	3698
10. exp calgranulin/ec [Endogenous Compound]	2298
11. 9 or 10	4798
12. 8 and 11	2731
13. exp Crohn disease/di [Diagnosis]	9738
14. exp ulcerative colitis/di [Diagnosis]	6189
15. 13 or 14	13053
16. 11 and 15	347
17. 12 or 16	2747
18. (fecal or faecal).tw.	82659
19. 17 and 18	2159
20. limit 19 to dd=20120901-20170531	1487
21. limit 19 to em=201235-201722	1975
22. 20 or 21	1995

For peer review only - http://bmjopen.bmj.com/site/about/guidelines.xhtml

Cochrane library (search on 31/05/2017)

calprotectin and (inflammatory bowel disease* or irritable bowel syndrome or crohn* disease or ulcerative colitis) - 158 hits
Other reviews: 6
Trials: 147
Technology assessments: 3
Economic evaluations: 2 (not of interest)

WoS: Science Citation Index and Conference Proceedings Citation Index 2012-2017 (search on 31/05/2017)

TS=(calprotectin and (inflammatory bowel disease* or irritable bowel syndrome or crohn* disease or ulcerative colitis)) – 1,063 hits

For peer review only

Supplement 2

Data extraction form: faecal calprotectin

First reviewer: KF Second reviewer: HF

**Study details**	
Study ID (Endnote ref)	
First author surname	
Year of publication	
Country	
Study setting	
Number of centres	
Duration of study	
Funding	
**Aim of the study**	
**Description of study format (study design / set up)**	
**Inclusion/exclusion criteria for patients**	
Inclusion criteria:	
Exclusion criteria:	
**Participants**	
**Item**	
Definition of included patients (primary care, referred, secondary care)	
Presentation (indication for FC testing)	
Previous investigations	
Total number of patients (samples) at baseline	
N excluded and reason	
N included in study	
N included in analysis	
Age mean (range)	
Adults (>60 years) n (%)	
Young adults (<18 years) n (%)	
Male n (%)	
Female n (%)	
**Index test**	
Test type	
Range in µg/g	
Cut-off in µg/g	
Cut-off pre-specified yes/no	
Time between taking the sample and freezing it	
Time between index test and reference standard	
Blinding to reference standard results	
Describe how index test was performed	
Reference standard	
Define patients and proportion who received reference standard 1	
Define patients and proportion who received reference standard 2	
Define patients and proportion who received no reference standard	
Blinding to index test results	
**Outcomes**	
Define differential diagnosis that the 2x2 applies to	
Definition of target condition (i.e. inclusion of conditions in organic category)	
Definition of IBS / functional disease	

For peer review only - http://bmjopen.bmj.com/site/about/guidelines.xhtml

Record test accuracy measures and 2x2 tables for all cut-offs and tests	
Proportion of patients referred if primary care population	
Proportion having received colonoscopy if primary care population	
Proportion of colonoscopies with abnormal findings n/N (%)	
**Authors' conclusion**	
**Reviewer's notes**	

For peer review only

Supplement 3 Between study variance of logit sensitivity and logit specificity of each random effects meta-analysis (for tests and clinical questions)

There was some statistical evidence (Chi-square = 14.145, 9df, P=0.1173) that the assumption of equal variances for tests is reasonable at the significance level of 0.05. However, the number of studies per test was small, and examination of the variances of the tests revealed that there is a substantial difference in the between-study variance of logit sensitivity and logit specificity of each test. The model did not converge when unequal variances were assumed between test types.

For clinical questions there was some statistical evidence (Chi-square = 13.328, 6df, P=0.03812) that the assumption of equal variance may not be reasonable for the 50µg/g cut-off, but reasonable for the 100µg/g cut-off (Chi-square = 2.3185, 6df, P=0.8882) with a much smaller number of studies. However, the models with non-equal variances did not converge, so results should be interpreted with caution.

**Test name**	**Between study variance (logitSe)**	**Between study variance (logitSp)**	**Correlation between logitSe and logitSp**
PhiCal	1.301	1.202	-0.94
EK-CAL	0.506	1.265	-0.47
Quantum-Blue	2.2012	0.1378	1.00
ELiA	0.9620	0.6593	0.132
**Clinical questions**			
IBD vs IBS	1.4131	0.4883	-0.81
IBD vs non-IBD	1.1198	0.4731	-0.81
Organic vs non-organic disease	0.1546	0.7890	0.48

logitSe logit sensitivity, logitSp logit specificity, IBD inflammatory bowel disease, IBS irritable bowel syndrome

For peer review only - http://bmjopen.bmj.com/site/about/guidelines.xhtml

Supplement 4 Excluded studies with reason

Studies excluded from new review

Reference of full text	Reason for exclusion
1. Anonymous. What is the faecal calprotectin test? Drug & Therapeutics Bulletin. 2014;52(9):102-4.	Review
2. Bai W, Boswell T. Clinical utility and outcome analysis of faecal calprotectin in Hawkes Bay District Health Board. New Zealand Medical Journal. 2016;129(1433):69-73.	No 2x2 data, 46/85 patients with FC test for differential diagnosis (IBD versus non-IBD) had no final diagnosis reported, FP only partially reported for patients with IBS diagnosis, not for non-IBD diagnoses, FN for IBD not reported
3. Bar-Gil Shitrit A, Koslowsky B, Livovsky DM, Shitrit D, Paz K, Adar T, Adler SN, Goldin E. A prospective study of fecal calprotectin and lactoferrin as predictors of small bowel Crohn's disease in patients undergoing capsule endoscopy. Scandinavian Journal of Gastroenterology. 2017;52(3):328-33.	Small bowel only, patient spectrum - all patients included had previous negative colonoscopy
4. Burri E, Manz M, Rothen C, Rossi L, Beglinger C, Lehmann FS. Monoclonal antibody testing for fecal calprotectin is superior to polyclonal testing of fecal calprotectin and lactoferrin to identify organic intestinal disease in patients with abdominal discomfort. Clinica Chimica Acta. 2013;416:41-7.	Overlap with previous SR
5. Burri E, Manz M, Schroeder P, Froehlich F, Rossi L, Beglinger C, Lehmann FS. Diagnostic yield of endoscopy in patients with abdominal complaints: incremental value of faecal calprotectin on guidelines of appropriateness. BMC Gastroenterology. 2014;14:57.	2x2 data is for risk of significant endoscopy finding by EPAGE alone versus EPAGE plus FC (not FC versus actual endoscopy finding)
6. Chang MH, Chou JW, Chen SM, Tsai MC, Sun YS, Lin CC, Lin CP. Faecal calprotectin as a novel biomarker for differentiating between inflammatory bowel disease and irritable bowel syndrome. Molecular Medicine Reports. 2014;10(1):522-6.	Patients with confirmed IBD/ IBS and healthy controls included, no 2x2 data, sensitivity and specificity and predictive values reported for 'optimal' cut off from ROC for IBD patients only
7. Chapman TP, Chen LY, Leaver L. Investigating young adults with chronic diarrhoea in primary care. BMJ. 2015;350:h573.	Case study, report on best clinical practice
8. Egea Valenzuela J, Pereniguez Lopez A, Perez Fernandez V, Alberca de Las Parras F, Carballo Alvarez F. Fecal calprotectin and C-reactive protein are associated with positive findings in capsule endoscopy in suspected small bowel Crohn's disease. Revista Espanola de Enfermedades Digestivas. 2016;108(7):394-400.	Small bowel only, spectrum bias - all patients included had previous negative colonoscopy
9. Egea-Valenzuela J, Alberca-de-Las-Parras F, Carballo-Alvarez F. Fecal calprotectin as a biomarker of inflammatory lesions of the small bowel seen by videocapsule endoscopy. Revista Espanola de Enfermedades Digestivas. 2015;107(4):211-4.	Small bowel only, spectrum bias - all patients included had previous negative colonoscopy
10. Elias SG, Kok L, de Wit NJ, Witteman BJ, Goedhard JG, Romberg-Camps MJ, Muris JW, Moons KG. Is there an added value of faecal calprotectin and haemoglobin in the diagnostic work-up for primary care patients suspected of significant colorectal disease? A cross-sectional diagnostic study. BMC Medicine. 2016;14(1):141.	Risk prediction model modelling the incremental diagnostic accuracy of FC test in addition to physical examination predictors
11. Emmanuel A, Landis D, Peucker M, Hungin AP. Faecal biomarker patterns in patients with symptoms of irritable bowel syndrome. Frontline Gastroenterology. 2016;7(4):275-82.	FC for characterisation of demographics and IBS subtypes in patients with known IBS
12. Garcia F, Martinez C, Juliao H, Bautista-Molano W, Valle-Onate R, Rueda JC, Romero-Sanchez C. Autoantibodies and fecal calprotectin levels in a group of Colombian patients with inflammatory bowel disease. Gazzetta Medica Italiana Archivio per le Scienze Mediche. 2017;176(3):132-41.	IBD patients only, IBS excluded, not test accuracy but characterisation of severity of IBD patients
13. Hale MF, Drew K, McAlindon ME, Sidhu R. The diagnostic accuracy of faecal calprotectin and small bowel capsule endoscopy and their correlation in suspected isolated small bowel Crohn's disease. European Journal of Gastroenterology & Hepatology. 2016;28(10):1145-50.	Small bowel only, unlcear whether included patients had normal colonoscopy
14. Hoog CM, Bark LA, Brostrom O, Sjoqvist U. Capsule endoscopic findings correlate with fecal calprotectin	Patients included have had negative colonoscopy and positive capsule endoscopy, small bowel only

For peer review only - http://bmjopen.bmj.com/site/about/guidelines.xhtml

and C-reactive protein in patients with suspected small-bowel Crohn's disease. Scandinavian Journal of Gastroenterology. 2014;49(9):1084-90.	
15. Jensen MD, Nathan T, Rafaelsen SR, Kjeldsen J. Ileoscopy reduces the need for small bowel imaging in suspected Crohn's disease. Danish Medical Journal. 2012;59(9):A4491.	Comparison is ileocolonoscopy versus ileocolonoscopy plus small bowel capsule endoscopy
16. Joosen AMCP, Kok MB, Van Der Linden IJM, Bozkurt Z, Broos H, Van Pelt J, Van Heerde M, De Groot MJM. Analytical and clinical evaluation of faecal calprotectin. [Dutch] Analytische en klinische evaluatie van de bepaling van calprotectine in feces. Nederlands Tijdschrift voor Klinische Chemie en Laboratoriumgeneeskunde. 2013;38(4):196-201.	not sufficient data for 2x2 table and no demographics about patients, don't know whether adults and/or children, only reports PPV, NPV and concordance (TP+TN/N)
17. Kalantari H, Taheri A, Yaran M. Fecal calprotectin is a useful marker to diagnose ulcerative colitis from irritable bowel syndrome. Advanced Biomedical Research. 2015;4:85.	UC patients only, 21 patients with other colonoscopy findings (Crohn's colitis, microscopic colitis, collagenous colitis, or other) were excluded
18. Kok L, Elias SG, Witteman BJM, Goedhard JG, Muris JWM, Moons KGM, De Wit NJ. Diagnostic accuracy of point-of-care fecal calprotectin and immunochemical occult blood tests for diagnosis of organic bowel disease in primary care: The cost-effectiveness of a decision rule for abdominal complaints in primary care (CEDAR) study. Clinical Chemistry. 2012;58(6):989-98.	Overlap with previous SR
19. Kopylov U, Yung DE, Engel T, Avni T, Battat R, Ben-Horin S, Plevris JN, Eliakim R, Koulaouzidis A. Fecal calprotectin for the prediction of small-bowel Crohn's disease by capsule endoscopy: a systematic review and meta-analysis. European Journal of Gastroenterology & Hepatology. 2016;28(10):1137-44.	Small bowel only, mix of patients with suspected and confirmed IBD, 5/7 studies investigated patients with negative endoscopy
20. Kotze LM, Nisihara RM, Marion SB, Cavassani MF, Kotze PG. FECAL CALPROTECTIN: levels for the ethiological diagnosis in Brazilian patients with gastrointestinal symptoms. Arquivos de Gastroenterologia. 2015;52(1):50-4.	No 2x2 data, number of IBD and IBS reported but FC levels only reported as means and medians, no measures of accuracy reported to derive 2x2 data from, data requested – no response
21. Koulaouzidis A, Sipponen T, Nemeth A, Makins R, Kopylov U, Nadler M, Giannakou A, Yung DE, Johansson GW, Bartzis L, Thorlacius H, Seidman EG, Eliakim R, Plevris JN, Toth E. Association Between Fecal Calprotectin Levels and Small-bowel Inflammation Score in Capsule Endoscopy: A Multicenter Retrospective Study. Digestive Diseases & Sciences. 2016;61(7):2033-40.	Small bowel only, mix of suspected and confirmed IBD
22. Lance S, White C. An audit on the appropriate use of faecal calprotectin testing within the Taranaki DHB: a case for a more discerning approach. New Zealand Medical Journal. 2015;128(1417):24-9.	Not sufficient information to derive 2x2 data
23. Li LQ, Zeng J, Wang S, Chen X, Jiang Z. Fecal calprotectin for diagnosis of inflammatory bowel disease: A meta-analysis. [Chinese]. World Chinese Journal of Digestology. 2016;24(31):4272-8.	Meta-analysis in Chinese, different question as included studies enrolled patients with confirmed diagnosis and healthy controls
24. Licata A, Randazzo C, Cappello M, Calvaruso V, Butera G, Florena AM, Peralta S, Camma C, Craxi A. Fecal calprotectin in clinical practice: a noninvasive screening tool for patients with chronic diarrhea. Journal of Clinical Gastroenterology. 2012;46(6):504-8.	Overlap with previous SR
25. Lozoya Angulo ME, de Las Heras Gomez I, Martinez Villanueva M, Noguera Velasco JA, Aviles Plaza F. Faecal calprotectin, an useful marker in discriminating between inflammatory bowel disease and functional gastrointestinal disorders. Gastroenterologia y Hepatologia. 2017;40(3):125-31.	Adults and children, results reported combined, author is unable to send results for adults separately
26. Manz M, Burri E, Rothen C, Tchanguizi N, Niederberger C, Rossi L, Beglinger C, Lehmann FS. Value of fecal calprotectin in the evaluation of patients with abdominal discomfort: An observational study. BMC Gastroenterology. 2012:5.	Overlap with previous SR
27. McFarlane M, Chambers S, Malik A, Lee B, Sung E, Nwokolo C, Waugh N, Arasaradnam R. Clinical outcomes at 12 months and risk of inflammatory bowel disease in patients with an intermediate raised fecal	Included patients with intermediate FC outcomes only

For peer review only - http://bmjopen.bmj.com/site/about/guidelines.xhtml

calprotectin: a 'real-world' view. BMJ Open. 2016;6(6):e011041.	
28. McFarlane M, Chambers S, Malik A, Lee B, Sung E, Nwokolo C, Waugh N, Arasaradnam R. Is NICE too optimistic about savings from normal faecal calprotectin results? Journal of Gastroenterology and Hepatology Research. 2016;5(1):1895-8.	Included patients with FC negative results only
29. Menees SB, Powell C, Kurlander J, Goel A, Chey WD. A meta-analysis of the utility of C-reactive protein, erythrocyte sedimentation rate, fecal calprotectin, and fecal lactoferrin to exclude inflammatory bowel disease in adults with IBS. American Journal of Gastroenterology. 2015;110(3):444-54.	Patients with confirmed IBD
30. Olsen PAS, Fossmark R, Qvigstad G. Fecal calprotectin in patients with suspected small bowel disease - a selection tool for small bowel capsule endoscopy? Scandinavian Journal of Gastroenterology. 2015;50(3):272-7.	Small bowel only, patients had negative / inconclusive bowel investigation
31. Osipenko MF, Livzan m A, Skalinskaia MI, Lialiukova EA. [Fecal calprotectin concentration in the differential diagnosis of bowel diseases]. Terapevticheskii Arkhiv. 2015;87(2):30-3.	Not sufficient information to derive 2x2 data
32. Polewiczowska B, Ashraf W, Grainger S, Khan SR, Sandhu K. Negative faecal calprotectin (FC) is a strong predictor of negative small bowel capsule endoscopy. United European Gastroenterology Journal. 2013;1):A188-A9.	Small bowel only, not enough information on included patients
33. Rodriguez-Moranta F, Lobaton T, Rodriguez-Alonso L, Guardiola J. [Fecal calprotectin in the diagnosis of inflammatory bowel diseases]. Gastroenterologia y Hepatologia. 2013;36(6):400-6. Calprotectina fecal en el diagnostico de enfermedades inflamatorias.	Review
34. Samuel S, Ragunath K. Colonoscopy, inflammatory bowel disease. Endoscopy. 2013;45(4):289-91.	Review
35. Schulz C, Wex T, Arnim UV, Malfertheiner P. Validation of Two Calprotectin Rapid Tests in Daily Routine. Clinical Laboratory. 2016;62(7):1249-54.	Patients had confirmed IBD / IBS diagnosis, reference standard was ELISA for test comparison
36. Seenan JP, Thomson F, Rankin K, Smith K, Gaya DR. Are we exposing patients with a mildly elevated faecal calprotectin to unnecessary investigations? Frontline Gastroenterology. 2015;6(3):156-60.	Only patients with 100-200 microgramm/g FC characterised
37. Sipponen T, Haapamaki J, Savilahti E, Alfthan H, Hamalainen E, Rautiainen H, Koskenpato J, Nuutinen H, Farkkila M. Fecal calprotectin and S100A12 have low utility in prediction of small bowel Crohn's disease detected by wireless capsule endoscopy. Scandinavian Journal of Gastroenterology. 2012;47(7):778-84.	Overlap with previous SR - excluded as only investigation of small bowel
38. Smith LA, Gaya DR. Utility of faecal calprotectin analysis in adult inflammatory bowel disease. World Journal of Gastroenterology. 2012;18(46):6782-9.	Review
39. Sood R, Gracie DJ, Law GR, Ford AC. Systematic review with meta-analysis: the accuracy of diagnosing irritable bowel syndrome with symptoms, biomarkers and/or psychological markers. Alimentary Pharmacology & Therapeutics. 2015;42(5):491-503.	Not FC
40. Sydora MJ, Sydora BC, Fedorak RN. Validation of a point-of-care desk top device to quantitate fecal calprotectin and distinguish inflammatory bowel disease from irritable bowel syndrome. Journal of Crohns & Colitis. 2012;6(2):207-14.	Overlap with previous SR - excluded as patients had known diagnosis prior to the study
41. Turvill J. High negative predictive value of a normal faecal calprotectin in patients with symptomatic intestinal disease. Frontline Gastroenterology. 2012;3(1):21-8.	Overlap with previous SR
42. von Arnim U, Wex T, Ganzert C, Schulz C, Malfertheiner P. Fecal calprotectin: a marker for clinical differentiation of microscopic colitis and irritable bowel syndrome. Clinical & Experimental Gastroenterology. 2016;9:97-103.	Spectrum bias - only included microscopic colitis patients (mixed active and in remission), no 2x2 data
43. Wagner M, Sjoberg K, Vigren L, Olesen M, Benoni C, Toth E, Carlson M. Elevated fecal levels of eosinophil granule proteins predict collagenous colitis in patients referred to colonoscopy due to chronic non-bloody	Not sufficient information to derive 2x2 data

For peer review only - http://bmjopen.bmj.com/site/about/guidelines.xhtml

	diarrhea. Scandinavian Journal of Gastroenterology. 2016;51(7):835-41.	
44.	Wang S, Shi H, Lu H, Wang F, Wei J, Wang Z, Liu C, Yuan B. Significance of fecal calprotectin in differential diagnosis of whole spectrum of digestive system diseases. [Chinese]. Chinese Journal of Gastroenterology. 2012;17(4):237-9.	Included patients had confirmed disease
45.	Wang S, Wang Z, Shi H, Heng L, Juan W, Yuan B, Wu X, Wang F. Faecal calprotectin concentrations in gastrointestinal diseases. Journal of International Medical Research. 2013;41(4):1357-61.	no 2x2 data, sens / spec reported for 2 cut-offs from ROC, but no predictive value – request for further data failed, e-mail bounced back
46.	Wassell J, Wallage M, Brewer E. Evaluation of the Quantum Blue rapid test for faecal calprotectin. Annals of Clinical Biochemistry. 2012;49(1):55-8.	Overlap with previous SR - excluded as no patient details reported

Abstracts

1.	Alzoubaidi D, Asser L, Price T, Lithgo K, Housley D, Johnson MW. Is a false positive faecal calprotectin as false as you think? Gut. 2015;64:A238.	only considered FC+ patients with normal endoscopy
2.	Astle VI, Lewis NR. Under-utilisation of faecal calprotectin to exclude ibd in patients with functional bowel disorders. Gut. 2014;63:A207-A8.	Cannot derive meaningful 2x2 data
3.	Atef S, Abdel Rahman S. The diagnostic performance of the combined use of fecal calprotectin and occult blood tests for screening of organic bowel disease in primary care setting. Biochimica Clinica. 2013;37:S658.	No test and cut off reported
4.	Aujla UI, Hayee B, Sherwood R, Chung-Faye G. Predictive value of faecal calprotectin in patients undergoing colonoscopy: A cost-effective analysis. United European Gastroenterology Journal. 2013;1):A517.	Cannot derive meaningful 2x2 table
5.	Caccaro R, Lollo G, Hatem G, Ugoni A, Buda A, D'Odorico A, Galeazzi F, D'Inca R, Savarino EV, Sturniolo GC. Are non-invasive markers of gastro-intestinal disease predictors of enteropathy at small bowel capsule endoscopy? United European Gastroenterology Journal. 2014;1):A347-A8.	small bowel only in colonoscopy negative patients
6.	Conroy S, Hale M, Cross S, Swallow K, Sidhu R, Sargur R, et al. Does the performance of faecal calprotectin testing in primary care differentiate patients with inflammatory bowel disease? Gut. 2015;64:A25.	Patients are children and adults no proportions reported
7.	Demir OM, Ahmed Z, Logan RPH. Optimising the use of faecal calprotectin for early diagnosis of IBD in primary care. Journal of Crohn's and Colitis. 2013;7:S8-S9.	Cannot derive meaningful 2x2 data
8.	Digby J, Steele RJC, Strachan JA, Mowat C. Stool tests can potentially rule out significant bowel disease in symptomatic patients in primary care. United European Gastroenterology Journal. 2014;1):A47.	Overlap of study population with Mowat 2016 suspected
9.	Eccles J, Neely A, Lynch M, Ferguson CB, Morrison G. An overview of the impact of fecal calprotectin testing in the management of patients within the gastroenterology outpatient clinic in a general hospital. Gastroenterology. 2014;1):S-798.	Over 30% are children 15-18 years
10.	Greig E, Gore S, Staveley K, Phillips I, Benneyworth R, Matull R, Thole S, Williams M. Delivering cost effective management for Irritable Bowel Syndrome (IBS) across Somerset. Gut. 2015;64:A192-A3.	Known cases of IBD and new cases included, proportions not reported
11.	Hale MF, Drew K, McAlindon ME, Lobo AJ, Sidhu R. Faecal calprotectin in patients with suspected small bowel Crohn's disease: Correlation with small bowel capsule endoscopy. Gut. 2015;64:A83.	Colonoscopy negative patients only, investigation of small bowel only
12.	Hunt N, Allcock R, Sharma A, Myers M. Diagnostic performance of faecal calprotectin in primary care. Gut. 2014;63:A159.	Not enough information for 2x2 table
13.	Kotze L, Nisihara R, Cavassani M, Valarini S, Kotze P. Fecal calprotectin levels for the ethiological diagnosis in Brazilian patients with gastrointestinal symptoms. American Journal of Gastroenterology. 2014;109:S500.	Superseeded by full text, not enough data for 2x2 table, numbers of IBD and IBS don't add up to total, no numbers for FC levels or test accuracy measures
14.	Kwok R, Peter F, Page BP, Ahmed T, Tay DXH, Woo ASJ, Constantinos A, Yip BCH, Sze KCP, Wee EWL. Diagnostic performance of faecal calprotectin in Singaporean patients. Journal of Gastroenterology and Hepatology (Australia). 2016;31:212.	No 2x2 data, don't know definition of non-colitis group

For peer review only - http://bmjopen.bmj.com/site/about/guidelines.xhtml

15. Keny B, Gaikwad S, Parkar S, Dherai AJ, Shetty D, Desai D, Joshi A, Abraham P, Gupta T, Tester Ashavaid F. Fecal calprotectin-marker for inflammatory bowel disease. Indian Journal of Clinical Biochemistry. 2016;31 (1 Supplement 1):S59.	Cannot derive meaningful 2x2 table
16. Lee S, Borthwick H, Dhar A. Faecal calprotectin testing in primary and secondary care Are the current manufacturer's cut-off values clinically useful? Journal of Crohn's and Colitis. 2014;8:S155-S6.	Superseded by full text Lee 2013
17. Maheshwari P, Junagade P, Goulding C. Evaluation of the use of fecal calprotectin as a diagnostic aid for IBD in an Irish population. Journal of Crohn's and Colitis. 2014;8:S159.	Cannot derive meaningful 2x2 table
18. Malik A, Bowen D, Rees I. Usefulness of fecal calprotectin in clinical practice in a district general hospital. Gut. 2013;62:A65.	Duplicate abstract with Alrubaiy 2012
19. McFarlane M, Chambers S, Dhaliwal A, Patel A, Nwokolo C, Arasaradnam R. Six month clinical outcomes in patients with intermediate raised faecal calprotectin levels. International Journal of Surgery. 2015;23:S48.	intermediate FC levels only
20. McFarlane M, Dhaliwal A, Chambers S, Nwokolo C, Patel A, Arasaradnam R. Clinical outcomes in patients with intermediate raised faecal calprotectin levels. United European Gastroenterology Journal. 2014;1):A371-A2.	Not sufficient data for 2x2
21. Mishreki A, Bell H, Austin C, Sheppard S, Noblett S. Does a raised faecal calprotectin level correlate with a diagnosis of IBD on colonoscopic biopsies? Colorectal Disease. 2014;16:43.	No test and cut-off reported, clinical question not specified, differential diagnosis unknown
22. Mohammed N, Smale S. Positive calprotectin but negative investigations-what next? Gut. 2012;61:A236.	positive faecal calprotectin with normal endoscopic/radiological only
23. Moroni F, Winter JW, Morris AJ, Gaya DR. What is the clinical relevance of a mildly elevated faecal calprotectin detected in new referrals to the gastroenterology clinic? Gut. 2012;61:A78-A9.	only low and intermediate FC group included
24. Mukhtar A, Sivaramakrishnan N, Hassan F. Should the cut off values of faecal calprotectin for initiating further investigations be higher than current practice? Gut. 2016;65:A243-A4.	Only FC positive patients included
25. Oyaert M, Trouve C, Baert F, De Smet D, Langlois M, Vanpoucke H. Performance characteristics of faecal calprotectin testing for diagnosis of Inflammatory Bowel Disease. Acta Clinica Belgica. 2014;69:1-2.	Superseded by full publication
26. Pantaleoni S, Touscoz GA, Caviglia GP, Adriani A, Sguazzini C, Sapone N, Reggiani S, Rizzetto M, Astegiano M. Fecal calprotectin is an effective diagnostic tool that differentiates pathological from functional intestinal disorders. Digestive and Liver Disease. 2013;45:S104-S5.	patients with known diagnosis
27. Parker C, Lamb CA, Robinson M, Mansfield JC, Gunn M. Predicting inflammatory pathology at capsule enteroscopy: What is the utility of a raised faecal calprotectin? Gut. 2015;64:A74-A5.	excluded only FC positive patients
28. Patel KV, Zaman S, Fong S, Anderson SH. Comparison of video capsule endoscopy and faecal calprotectin as diagnostic tools in patients with abdominal symptoms suggestive of small bowel Crohn's Disease. Journal of Crohn's and Colitis. 2015;9:S153-S4.	small bowel only
29. Reed O, Doyle J, Murphy S. Faecal calprotectin to screen out IBS: Is it being used correctly? Irish Journal of Medical Science. 2015;184 (6 Supplement 1):S248.	incomplete data at time of analysis
30. Rouke JO, Dhaliwal A, Sagar V, Davies J, Milestone A. Use of faecal calprotectin in primary care to distinguish irritable bowel syndrome from inflammatory bowel disease. Gut. 2015;64:A86-A7. And<br>31. Dhaliwal A, J OR, Sagar V, Burdsall J, Ransford R, Milestone A. Use of faecal calprotectin pathway in primary care to distinguish irritable bowel syndrome from inflammatory bowel disease. United European Gastroenterology Journal. 2015;1):A237.	Identical, incomplete data available at time of analysis
32. Sartain S, Stone A. Faecal calprotectin levels of 50-200: Endoscopic findings and subsequent diagnoses. Gut. 2016;65:A197-A8.	Only intermediate FC levels
33. Shastri YM, Povse N, Stein J. Prospective evaluation of faecal tumour pyruvate kinase type M2 (M2-PK) in	IBD monitoring

For peer review only - http://bmjopen.bmj.com/site/about/guidelines.xhtml

	comparison to calprotectin in IBD patients. Journal of Crohn's and Colitis. 2013;7:S75-S6.	
34.	Shitrit AB, Braverman D, Paz K, Adar T, Koslowsky B, Goldin E. Fecal calprotectin and lactoferrin as biomarkers in patients undergoing capsule endoscopy. Gastroenterology. 2013;1):S424.	Small bowel only
35.	Taylor N, Hills E, Sheen C, Al-Bahrani A, Grellier L. An audit of faecal calprotectin testing in suspected inflammatory bowel disease in the under 45's. Journal of Crohn's and Colitis. 2013;7:S131.	Clinical question not specified, non-IBD group unknown
36.	Tomkins C, Zeino Z, Nwokolo C, Smith SC, Arasaradnam R. Faecal calprotectin analysis: Does the method matter? Gut. 2012;61:A173-A4.	No meaningful 2x2 data
37.	Wu J, Bolton L, Chapman C, Chey CS, Harrison E, Kinderman H, Johnson H, Richards-Taylor A, Weaver S, McLaughlin S. Faecal calprotectin has an acceptable sensitivity for detecting small bowel crohn's disease: Results from real world clinical practice. Gut. 2016;65:A145.	Known diagnosis and small bowel only
38.	Zaman S, Patel K, Goel R, Borrow DM, Anderson SH. Comparison of video capsule endoscopy and faecal calprotectin as diagnostic tools in patients with abdominal symptoms suggestive of small bowel crohn's disease. United European Gastroenterology Journal. 2013;1):A493.	Small bowel only
39.	Zuhra N, Sartain S, Gordon J, Lloyd D. Capsule endoscopy in the investigation and management of small bowel crohn's disease. Gut. 2016;65:A167-A8.	Small bowel only, incomplete FC data

Studies from previous review (Waugh et al. 2012)

1.	Ashorn S, Honkanen T, Kolho KL, Ashorn M, Valineva T, Wei B, et al. Fecal calprotectin levels and serological responses to microbial antigens among children and adolescents with inflammatory bowel disease. Inflamm Bowel Dis 2009;15:199–205.	Children
2.	Basumani P, Bardhan K, Eyre R, Ellis R, The Rotherham Team. Faecal calprotectin: Rotherham experience (slide presentation). BSG Away day 28/06/2012 (accessed 19 July 2013). Unpublished	Published and included as Banerjee 2015
3.	Canani RB, de Horatio LT, Terrin G, Romano MT, Miele E, Staiano A, et al. Combined use of noninvasive tests is useful in the initial diagnostic approach to a child with suspected inflammatory bowel disease. JPGN 2006;42:9–15.	Children
4.	Diamanti A, Panetta F, Basso MS, Forgione A, Colistro F, Bracci F, et al. Diagnostic work-up of inflammatory bowel disease in children: the role of calprotectin assay. Inflamm Bowel Dis 2010;16:1926–30.	Children
5.	Dolwani S, Metzner M, Wassell JJ, Yong A, Hawthorne AB. Diagnostic accuracy of faecal calprotectin estimation in prediction of abnormal small bowel radiology. Aliment Pharmacol Ther. 2004;20(6):615-21.	Small bowel only
6.	Fagerberg UL, Loof L, Myrdal U, Hansson LO, Finkel Y. Colorectal inflammation is well predicted by fecal calprotectin in children with gastrointestinal symptoms. JPGN 2005;40:450–5.	Children
7.	Henderson P, Casey A, Lawrence SJ, Kennedy NA, Kingstone K, Rogers P, et al. The diagnostic accuracy of fecal calprotectin during the investigation of suspected pediatric inflammatory bowel disease. Am J Gastroenterol 2012;107:941–9.	Children
8.	Manz M, Burri E, Rothen C, Tchanguizi N, Niederberger C, Rossi L, et al. Value of fecal calprotectin in the evaluation of patients with abdominal discomfort: an observational study. BMC Gastroenterol. 2012;12:5.	Overlap with Burri 2013
9.	Sidler MA, Leach ST, Day AS. Fecal S100A12 and fecal calprotectin as noninvasive markers for inflammatory bowel disease in children. Inflammatory Bowel Dis 2008;14:359–66.	Children
10.	Tomas AB, Vidal MV, Camps R. Fecal calprotectin as a biomarker to distinguish between organic and	Children

For peer review only - http://bmjopen.bmj.com/site/about/guidelines.xhtml

	functional gastrointestinal disease. Rev Esp Enferm Dig 2007;99:689–93.	
11.	Van de Vijver E, Schreuder AB, Cnossen WR, Muller Kobold AC, van Rheenen PF. Safely ruling out inflammatory bowel disease in children and teenagers without referral for endoscopy [published online ahead of print 2012]. Arch Dis Child 2012;12:1014–18.	Children

For peer review only

Supplement 5 Tables of study characteristics

Table of study characteristics - population

Study ID (author, year, country)	Aim	Population (N, setting, age, gender)	Study exclusions	Indication*	Clinical questions	Disease prevalence
Alrubaiy 2012 [1]; UK<br><br>Abstract	To evaluate the diagnostic value of FC as a non-invasive marker of bowel inflammation in routine out-patient gastroenterology clinic.	N=72<br>Setting: out-patients in gastroenterology clinic<br>Mean age: Female: 44 years<br>Male: 47 years<br>Female: 44/72 (61%)	NR	Chronic diarrhoea, abdominal pain, abdominal distension, rectal bleeding	Inflammatory versus non-inflammatory disease	Inflammatory disease (IBD): 2/72 (2.7%)
Banerjee 2015 [2]; UK	To assess the role of FC in aiding differential diagnosis, maximising the numbers in whom IBD could be ruled out, making colonoscopy unnecessary, yet not miss anyone with the disease.	N=119<br>Setting: newly referred patients to gastrointestinal clinic<br>Age:<br>≤40 years: 36%<br>41–60 years: 46%<br>>60 years: 18%<br>Female: 64/119 (54%)	Proven coeliac disease, pancreatic insufficiency	Diarrhoea of longer than 4 weeks duration	IBD versus IBS,<br>Organic versus non-organic disease	IBD: 12/119 (10%)
Bharathi 2005 [3]; UK<br><br>Abstract	To assess the negative predictive value of FC in excluding bowel pathology in young patients with suspected IBS.	N=58<br>Setting: NR<br>Mean age: NR<br>Female: NR	NR	Abdominal pain and/or loose stools	IBD versus IBS	IBD: 0/58
Boyd 2016 [4]; UK<br><br>Abstract	To assess an FC pilot pathway.	N=424<br>Setting: data from a primary care pilot FC pathway<br>Mean age: NR<br>Female: NR	Rejected samples	NR	IBD versus non-IBD	IBD: 23/424 (5.4%)
Burri 2013 [5]; Switzerland	To directly compare the test characteristics of FC and lactoferrin in their ability to detect organic intestinal disease	N=405<br>Setting: patients with abdominal discomfort referred for endoscopy<br>Median age (range): 63 years (18–97)<br>Female: 226 /405(56%)	<18 years	abdominal discomfort	Organic versus other disease	Organic: 143/405 (35%)
Carroccio 2003 [6]; Italy	To evaluate the positive and negative predictive values of the FC assay in identifying the organic causes of chronic diarrhea.	N=70<br>Setting: patients referred for chronic diarrhea to an outpatient gastroenterology clinic<br>Median age (range): 35 years (18–72) Female: 40/70 (57%)	Previous evaluation for chronic diarrhea, overt gastrointestinal bleeding, sigmoidoscopy or colonoscopy during the previous 2 years, familial adenomatous polyposis and hereditary nonpolyposis, colorectal cancer syndrome, pregnancy	Chronic diarrhea of unknown origin, lasting for more than 4 weeks, with or without abdominal pain	IBD versus IBS,<br>IBD versus non-IBD,<br>Organic versus non-organic disease	IBD: 11/70 (16%)

For peer review only - http://bmjopen.bmj.com/site/about/guidelines.xhtml

Study ID (author, year, country)	Aim	Population (N, setting, age, gender)	Study exclusions	Indication*	Clinical questions	Disease prevalence
Caviglia 2014 [7]; Italy	To asses FC concentration and evaluate the diagnostic accuracy in patients with and without bowel inflammation	N=66<br>Setting: first time referrals to outpatient gastroenterology clinic<br>Mean age (range): 42 (18-78) years<br>Female: 46/66 (70%)	Regular NSAIDs, known CRC or polyps, known acute gastrointestinal infection (diverticular disease)	Abdominal pain and/or altered bowel habit lasting at least 4 weeks	IBD versus IBS, Inflammatory versus non-inflammatory disease	IBD: 24/66 (36%)
Conroy 2017 [8]; UK	To assess the diagnostic accuracy of FC in the detection of IBD in a primary care setting.	N=410<br>Setting: FC ordered in primary care<br>Median age (range): 42 (16–91) years<br>Female: 248/410 (60%)	< 16 years, known IBD, FC testing initiated in secondary care	NR	IBD versus non-IBD, Organic versus non-organic disease	IBD: 11/410 (2.7%)
Damms 2008 [9]; Germany	To evaluate the diagnostic accuracy of the new calprotectin rapid test compared to an established ELISA test in detecting colonoscopy-proven intestinal inflammatory diseases and colorectal malignancies.	N=140<br>Setting: Patients referred to colonoscopy as in- or out-patients for clarification of lower GI symptoms<br>Mean age (range): 58 (20–85) years<br>Female: 78/144 (56%)	Known extraintestinal inflammatory diseases such as rheumatoid arthritis, chronic arthritis–sinusitis, urinary tract infection, patients on NSAID or anticoagulants	lower GI symptoms, altered bowel habit, weight loss, by reason of occult blood–rectal bleeding, search of tumor, resectional surgery, follow-up, or routine checkup	IBD versus functional disease, Organic versus non-organic disease	IBD: 18/140 (13%)
De Sloovere 2017 [10]; Belgium	To evaluate two newly developed automated FC immunoassays in terms of analytical and diagnostic performance.	N=229<br>Setting: out- and inpatients with clinical suspicion of IBD and stool sample for FC measurement<br>Median age (range): 34 (15–94) years<br>Female: 150/229 (66%)	<15 years of age, absence of clinical data, unclear diagnosis or insufficient sample volume	Suspicion of IBD	IBD versus IBS, IBD versus non-IBD	IBD: 46/229 (20%)
Dhaliwal 2015 [11]; UK	To determine the ability of FC to exclude IBD and optimal cut-offs for IBD in remission	N=311<br>Setting: FC samples in a secondary care setting<br>Mean age: NR<br>Female: 190/292 (65%)	NR	Suspected functional gastrointestinal disease (IBS) or IBD	IBD versus IBS	IBD: 148/311 (48%)
El-Badry 2010 [12]; Egypt	To evaluate the sensitivity and diagnostic accuracy of FC assay at different cut-off values in discriminating between functional and organic gastrointestinal disorders	N=39<br>Setting: patients referred to hospital for diagnostic differentiation of functional or organic bowel disorders<br>Mean age ±:SD:<br>IBD: 39.4 ± 15.9 years<br>IBS: 34.3 ± 15.75 years	On NSAIDs, aspirin, and/or anticoagulants, concomitant non-gastrointestinal diseases, (rheumatoid arthritis, other connective tissue inflammatory diseases or liver cirrhosis)	abdominal pain, chronic diarrhoea, weight loss and/or anorectal bleeding	IBD versus IBS, IBD vs non-IBD	IBD: 9/39 (23%)

For peer review only - http://bmjopen.bmj.com/site/about/guidelines.xhtml

Study ID (author, year, country)	Aim	Population (N, setting, age, gender)	Study exclusions	Indication*	Clinical questions	Disease prevalence
		Organic (non-IBD): 40.2 ± 14 years<br>Female: 19 /39 (49%)				
Garcia 2006 [13]; Spain	To assess the usefulness of FC to predict the presence of pathological colonoscopy and to analyze its use to discriminate between different organic diseases	N=190<br>Setting: patients undergoing colonoscopy for medical indications<br>Mean age±SD: Other: 59.5±6.0 years<br>Polyps: 60.21± 4.53 years<br>CRC: 71.75 ± 6.64 years<br>IBD: 35.84 ±15.4 years<br>Female: 98/190 (52%)	cardiopulmonary disease, kidney or liver disease, celiac disease, known malignancy	NR	Organic versus other disease	Organic disease: 73/190 (38%)
Hogberg 2017 [14]; Sweden	To assess the value of a point of care FIT and FC test in detecting CRC, high risk adenomas HRAs and IBD in primary care.	N=384<br>Setting: FC ordered in primary care<br>Median age: 63.0 years Female: 241/384 (64.6%)	Patients declined, emergency admittance, FC not returned	Abdominal pain, change of bowel habits, diarrhoea, constipation, rectal bleeding, urgency, anaemia, incomplete stool evacuation, weight loss, Melaena, palpable mass	IBD versus non-IBD, Organic versus other disease	IBD: 10/373 (2.6%)
Jang 2016 [15]; Korea	To evaluate the initial diagnostic ability of three faecal calprotectin kits in different bowel diseases	N=41<br>Setting: patients at hospital for workup of abdominal symptoms<br>Mean age ± SD (range):<br>CD patients: 22±5 (15–31)<br>UC patients: 41±21 (19–74)<br>Intestinal BD: 54±21 (39–69)<br>IBS: 17±12 (5–31)<br>Other colitis: 38±24 (11–69)<br>Female: 13/41 (32%)	Incomplete colonoscopy, no faecal samples, CRC, history of bowel resection, uncertain diagnosis after colonoscopy, regular aspirin and/or NSAIDs	abdominal pain, changes in bowel habits, and/or anorectal bleeding	IBD versus IBS	IBD: 31/41 (76%)
Kennedy 2015 [16]; UK	To determine the most effective use of FC in the diagnosis of GI disease in patients with no prior known GI disease, at the first presentation to GI services.	N=895<br>Setting: Data of FC of patients presenting to gastrointestinal services<br>Mean age: 33.1 years (IQR: 25.6–40.7)<br>Female: 581/895 (64.9)	< 16 years or >50 years, Patients with FC tests from other hospitals, patients with confirmed GI diagnosis, on IBD treatment, insufficient detail available, severe intercurrent illness, on aspirin/NSAIDs, on corticosteroids and/or aminosalicylates	Bloody diarrhoea, watery diarrhoea, rectal bleeding, constipation, abdominal pain, weight loss, bloating, vomiting, dyspepsia, fatigue, possible extraintestinal manifestations	IBD versus functional disease,<br>Organic versus non-organic disease	IBD: 91/895 (10%)
Kok 2012 [17]; Netherlands	To quantify the diagnostic accuracy of 3 biomarker tests for the inclusion or exclusion of OBD in patients with persistent	N=382<br>Setting: Referred patients to colonoscopy from primary care (recruitment was taking place at	<18 years old, unable to give informed consent, previously diagnosed with organic disease, or positive on triple	Abdominal pain, rectal bleeding, diarrhoea, fever and weight loss	Organic versus other disease	Organic disease: 99/382 (26%)

For peer review only - http://bmjopen.bmj.com/site/about/guidelines.xhtml

Study ID (author, year, country)	Aim	Population (N, setting, age, gender)	Study exclusions	Indication*	Clinical questions	Disease prevalence
	lower-abdomen complaints in primary care, who need colonoscopy referral	the GP practice or straight after registering for endoscopy<br>Median age (range): 60 (18-91) years<br>Female: 209/382 (54.7%)	feces test (TFT), not requiring endoscopy			
Labaere 2014 [18]; Belgium	To evaluate six FC tests and to determine their performance for diagnosis and follow up of IBD patients	N=31<br>Setting: referred for colonoscopy to gastroenterology<br>Mean age: 36 years (16-75)<br>Female: 21/31 (68%)	Inconclusive histopathological and endoscopic evaluation, extremely watery consistency of the stool sample	Chronic diarrhoea and/or abdominal pain, iron deficiency anaemia, unexplained weight loss, or a family history of IBD	IBD versus non-IBD	IBD: 12/31 (39%)
Lee 2014 [19]; UK<br><br>Abstract	To determine whether the manufacturer's cut-off for referral are clinically useful in making a positive diagnosis in patients presenting with chronic diarrhoea.	N=122<br>Setting: FC test results from primary and secondary care<br>Mean age: NR<br>Female: NR	NR	chronic diarrhoea	Organic versus non-organic disease	Organic: 23/122 (19%)
Li 2006 [20]; China	To assess the value of FC in differential diagnosis of IBS.	N=240<br>Setting: outpatients and hospitalised patients undergoing endoscopy<br>Mean age ± SD:<br>IBS: 48±19years<br>Inflammation:42±16 years<br>Female: 121/240 (50%)	Upper gastrointestinal symptoms, stomach/small intestinal disease, diseases of heart, lung, liver, kidney, nerve, mental disorder, alcoholic, pregnant women, drug addiction, long term use of NSAIDs, colorectal adenomas	NR	Inflammatory versus non-inflammatory disease	Inflammation: 60/240 (25%)
Licata 2012 [21]; Italy	To assess the diagnostic performance of FC as a stool-screening biomarker for organic intestinal disease	N=346<br>Setting: outpatients with unexplained chronic, non-bloody diarrhoea referred for colonoscopy<br>Median age (range):<br>Inflammation: 41 (17-80) years<br>No inflammation: 38 (18-87) years<br>Female: 201/346 (58%)	Bleeding, known colorectal or gastric neoplasia, familial adenomatous polyposis and hereditary nonpolyposis colorectal cancer syndrome, history of colonic surgery, recent respiratory or urinary tract infection, acute infectious disease, pregnancy and alcohol abuse, on NSAIDs, aspirin, or anticoagulants within the previous month, CRC	Unexplained chronic (≥4 wk), non-bloody diarrhoea	Inflammatory versus non-inflammatory disease	Inflammation: 142/346 (41%)
Limburg 2000 [22]; USA	To assess and compare calprotectin and hemoglobin (Hb) as stool screening biomarkers for	N=128<br>Setting: Patients referred to colonoscopy	Abnormalities on GI x-rays, bleeding, GI endoscopy performed within preceding 2 weeks, CRC syndrome	Chronic diarrhea (≥4 wk duration) of unknown origin or chronic colitis of unknown activity	Inflammatory versus non-inflammatory disease	Inflammatory disease: 29/128 (23%)

For peer review only - http://bmjopen.bmj.com/site/about/guidelines.xhtml

Study ID (author, year, country)	Aim	Population (N, setting, age, gender)	Study exclusions	Indication*	Clinical questions	Disease prevalence
	colorectal inflammation in unexplained chronic diarrhoea	Mean age (range): 57 years (21-85)<br>Female: 84/128 (66%)				
Lizvan 2015 [23]; Russia	To assess the informational content of fecal noninvasive tests (calprotectin, transferrin, hemoglobin) in complex diagnosis of diseases of intestines	N=52<br>Setting: unclear<br>Mean age (range): 38.6 years (18-49)<br>Female: unclear	Anxiety symptoms, weight loss, consistent abdominal pain, fever, pathology in abdominal organs, anaemia, increased red blood cell sedimentation rate, abdominal surgery in the past 6 months prior to enrolment, NSAIDs, antibacterial agents within one month of enrolment	Abdominal pain or discomfort, changes in stool frequency over the last 3 months	Organic versus non-organic disease (IBS)	Organic disease: 36/52 (69%)
Mowat 2016 [24]; UK	To study the diagnostic accuracies of quantitative FHb and FC tests in patients presenting to primary care with bowel symptoms	N=1031<br>Setting: referred patients from primary care<br>Median age (range): 64 years (16–90)<br>Female: 564/1031 (55%)	Faecal sample not suitable for analysis,<br>returned samples outside the study period,<br>known IBD, no colonoscopy, OGD only	Rectal bleeding, anaemia, diarrhoea, altered bowel habit, abdominal pain and weight loss	IBD versus non-IBD, Organic versus non-organic disease	IBD: 34/1031 (3.3%)
Otten 2008 [25]; Netherlands	To evaluate the diagnostic accuracy of two new rapid calprotectin and lactoferrin fecal tests in assessing colonic inflammation in patients with chronic abdominal complaints.	N=139<br>Setting: patients referred for endoscopy or sigmoidoscopy by the GP (80%) or the gastroenterologist (20%)<br>Mean age: IBS: 52.3 years<br>IBD: 44.5 years<br>Female: 61/114 (54%)	<18 years, patients with a history of colonic surgery and those with iron deficiency	Bloating, pain, change in defecation frequency or consistency, or blood and mucus in stool	IBD versus IBS	IBD 23/139 (17%)
Oyaert 2014 [26]; Belgium	To compare two FC assays regarding their reliability in the use of diagnosis of IBD	N=183<br>Setting: out- and inpatients with suspicion of IBD and FC<br>Median age (range): 32 years (14-89)<br>Female: 104/183 (57%)	<14 years, previous IBD diagnosis, no colonoscopy, unclear diagnosis (indeterminate colitis), inability to collect enough faecal samples	Diarrhoea, mucous, bloody stools, weight loss, abdominal pain and cramping	IBD versus non-IBD	IBD: 51/183 (27.9%)
Oyaert 2017 [27]; Belgium	To evaluate six different automated faecal calprotectin immunoassays for the diagnosis of IBD.	N=86<br>Setting: Patients in secondary care with suspicion of IBD and FC<br>Median age (range): 35 years (14-94)<br>Female: N=61/105 (58%)	<14 years, previous IBD diagnosis, no ileocolonoscopy, unclear diagnosis (e.g. indeterminate colitis), inability to collect enough faecal sample	Diarrhoea, mucous or bloody stools, weight loss, and abdominal pain and cramping	IBD versus non-IBD	IBD: 21/86 (24%)
Pavlidis 2013 [28]; UK	To assess the diagnostic performance of FC in routine general and the impact of different cut-offs	N=962<br>Setting: Suspected IBS patients in primary care<br>Mean age ± SD: 33 ±7 years	<18 and >45 years, previous diagnosis of IBD, missing clinical data, diarrhoea for <2 weeks	Persistent abdominal pain, bloating, alteration in stool form or frequency, passage of mucus	Organic versus non-organic disease	Organic disease: 94/962 (9.8%)

For peer review only - http://bmjopen.bmj.com/site/about/guidelines.xhtml

Study ID (author, year, country)	Aim	Population (N, setting, age, gender)	Study exclusions	Indication*	Clinical questions	Disease prevalence
	on endoscopy and referral rates.	Female: 577/962 (60%)				
Rosenfeld 2016 [29]; Canada	To evaluate the impact of FC test results for the differentiation of GI symptoms as IBS or IBD.	N=279<br>Setting: patients for whom gastroenterologist considered FC to be an appropriate next step in diagnosis and/or management of symptoms<br>Mean age ± SD (range): 39.4 ± 13.5 years (19-79)<br>Female: NR	ischemic colitis, infectious enterocolitis or colorectal cancer, pregnancy, history of extensive bowel resection, ostomy, ileoanal pouch, NSAIDS, inability to collect a stool sample	Gastrointestinal symptoms	IBD versus IBS	IBD: 4/50 (8%)
Schoepfer 2008 [30]; Switzerland	To determine the accuracy of 4 faecal markers in a patient group admitted for workup of GI symptoms.	N=136<br>Setting: Outpatients and inpatients admitted to colonoscopy ± upper endoscopy<br>Mean age (range): CD: 41 years (20–78)<br>UC: 45 years (23–72)<br>IBS: 40 years (20–79)<br>Female: 54/94 (57%)	Incomplete ileocolonoscopy, microscopic colitis, infectious ileocolitis, colorectal cancer, colorectal polyps, unclear diagnosis (e.g., indeterminate colitis), inability to collect fecal samples, history of colorectal or small bowel surgery, regular intake of aspirin and/or an NSAID (≥2 tablets/week).	Abdominal pain, altered bowel habit, and/or anorectal bleeding	IBD versus IBS	IBD: 64/94 (68%)
Schroeder 2007 [31]; Germany	To evaluate the clinical utility of FC to detect active gastrointestinal inflammation in patients suggestive of either having IBD or IBS.	N=76<br>Setting: patients referred for diagnostic clarification of chronic diarrhoea to tertiary referral practices<br>Median age (range):<br>CD: 40 (25–59) years<br>UC: 38 (24–75) years<br>IBS: 43 (20–72) years<br>Female: 43/76 (57%)	Previous evaluation for chronic diarrhoea, rectal bleeding, sigmoidoscopy or colonoscopy during previous 2 months, colorectal cancer syndrome and pregnancy	Chronic diarrhoea	IBD versus IBS	IBD: 45/76 (59%)
Sharbatdaran 2018[32] Iran	To evaluate the level of fecal calprotectin in patients with IBD and patients without inflammatory diseases of the colon	N=90<br>Setting: Referred to secondary care Mean age ± SD: 34.69±10.42 years<br>Female: 51/90 (56.7%)	Chronic disease and illness that caused fever, Patients with calprotectin level of 50-200µg	Clinical symptoms of colon diseases	IBD versus non-IBD	IBD: 45/90 (50%)
Shitrit 2007 [33]; Israel	To assess the predictive value of faecal calprotectin in organic colonic disease.	N=72<br>Setting: patients referred to gastroenterology for colonoscopy<br>Mean age ± SD: 58 ± 20 years<br>Female: 35/72 (48%)	NSAIDs, concomitant serious illness, pregnancy, alcohol abuse, respiratory tract infection	NR	Organic versus non-organic disease	Organic disease: 28/72 (39%)
Sostres 2017[34]; Spain	To evaluate the diagnostic accuracy of FC in	N=171	NR	Rectal bleeding, change of bowel habits, anaemia	Organic versus non-organic disease	Organic disease: 37/171 (21.6%)

For peer review only - http://bmjopen.bmj.com/site/about/guidelines.xhtml

Study ID (author, year, country)	Aim	Population (N, setting, age, gender)	Study exclusions	Indication*	Clinical questions	Disease prevalence
Abstract	symptomatic patients referred for diagnostic colonoscopy.	Setting: Referred for diagnostic colonoscopy Median age (IQR): 62 (51-68) years Female: 73/171 (42.7)				
Tan 2016[35]; UK Abstract	To evaluate the FC diagnostic referral pathway for primary care practitioners.	N=731 Setting: FC tests performed in primary care Median age: 40 years Female: 461/731 (63%)	NR	NR	IBD versus non-IBD	IBD: 15/731 (2.1%)
Tibble 2002 [36]; UK	To determine if the use of FC is useful in differentiating between patients with organic and nonorganic disease.	N=602 Setting: patients referred to gastroenterology outpatient department by general practitioners Median age (range): 40 years (18–90) Female: 371/602 (62%)	Symptoms of oesophageal reflux, gastroesophageal pathology, or functional or isolated dyspepsia, known IBD, colorectal carcinoma, and serious cardiopulmonary, hepatic, renal, neurologic, and psychiatric disease	Abdominal pain, diarrhoea, constipation, anaemia, bleeding, weight loss	Organic disease versus other disease	Organic disease: 263/602 (44%)
Turvill 2012 [37]; UK	To determine the NPV of a normal faecal calprotectin in excluding organic intestinal disease in patients with intestinal symptoms referred unselectively from primary care.	N=630 Setting: patients with intestinal symptoms referred from primary care to department of gastroenterology Mean age: Normal FC: 40.5 years Raised FC: 41 years Female: 442/630 (70%)	Patients with fast track colorectal symptoms	Pain/discomfort, diarrhoea, constipation, bloating, bleeding	Organic versus non-organic disease	Organic disease: 109/630 (17%)
Turvill 2016 [38]; UK	To evaluate the care pathway and recruit five primary care practices to conduct an FC implementation assessment.	N=262 Setting: patients presenting to primary care with new lower gastrointestinal symptoms Mean age ± SD: 36.8±10.9 years Female: 183/262 (70%)	Patients in whom the GP suspected cancer	Diarrhoea, alternating bowel habit, pain, bloating, constipation	Organic versus non-organic disease	Organic disease: 26/262 (9.9%)

Table of study characteristics – investigations

Study ID (author, year, country)	FC test and cut-off	Reference standard	Proportion without ref standard excluded	Proportion of analysis sample with reference standard	Definition target condition	Definition non-target condition
Alrubaiy 2014 [1]; UK Abstract	NR 50µg/g	Colonoscopy +/- capsule endoscopy or follow up at next clinic visit	None	28/54 (52%) colonoscopy +/- capsule endoscopy 26/54 (48%) follow up at next clinic visit	IBD	No Bowel inflammation
Banerjee 2015 [2]; UK	IDK ELISA 8 µg/g 25µg/g	Colonoscopy with histology	22/219 excluded from study	100%	IBD (excluding microscopic colitis)	Normal colonoscopy and histology

For peer review only - http://bmjopen.bmj.com/site/about/guidelines.xhtml

	50µg/g<br>75µg/g<br>100µg/g<br>150µg/g					
Bharathi 2005 [3];<br>UK<br><br>Abstract	PhiCal ELISA<br>60µg/g	OGD, flexible sigmoidoscopy, colonoscopy,<br>ultrasound, small bowel studies or clinical grounds	None	38/58 imaging<br>4/58 clinical grounds<br>16/58 unclear	IBD	IBS, non-intestinal, NSAIDS use, diverticulosis
Boyd 2016 [4];<br>UK<br><br>Abstract	NR<br>50µg/g<br>150µg/g	Investigation for referred not reported or median of 12 months (range 1-37) follow-up	None	Referred patients: NR<br><br>Non-referred patients: follow up	IBD (excluding microscopic colitis)	Gastroenteritis, microscopic colitis and IBS
Burri 2013 [5];<br>Switzerland	EK-CAL ELISA and PhiCal ELISA<br>50µg/g	Colonoscopy and histology +/- upper OGD	None recorded	70/405 with additional OGD	Organic: Esophagitis, erosive gastritis, gastric ulcer, gastric carcinoma, colitis (infectious colitis, CD, UC, diverticulitis, microscopic colitis), adenomatous polyp, CRC	Normal colonoscopy, hyperplastic polyp
Carroccio 2003 [6];<br>Italy	Calprest ELISA<br>50 µg/g<br>100 µg/g	Sigmoidoscopy or colonoscopy both with biopsy	None recorded	Sigmoidoscopy with biopsy if <40 years of age or colonoscopy with biopsy if >40	IBD (including microscopic colitis)	Colonoscopy / sigmoidoscopy negative and Manning criteria positive
Caviglia 2014 [7];<br>Italy	Calprest ELISA<br>50µg/g<br>100µg/g<br>150µg/g	Colonoscopy with biopsies	None reported	100%	IBD (including indeterminate colitis)	IBS
Conroy 2017 [8];<br>UK	IDK ELISA<br>50 µg/g<br>100 µg/g<br>125 µg/g<br>150 µg/g<br>250 µg/g	Colonoscopy, sigmoidoscopy, radiological features or small bowel video capsule endoscopy	None reported	146/410 (36%) colonoscopy (n=133) or sigmoidoscopy (n=13), 104/410 (25%) other investigation, 160 (39%) no investigation	IBD (including collagenous colitis, lymphocytic colitis)	Non-IBD (no further investigation and non-IBD organic conditions: CRC, colorectal adenomatous polyps, diverticulitis, appendicitis and diversion proctitis)
Damms 2008 [9];<br>Germany	EK-CAL ELISA<br>50 µg/g<br>Prevista qualitative rapid	Colonoscopy with histology	None recorded	100%	IBD (UC and CD)	normal diagnostic findings
De Sloovere 2017 [10]<br>Belgium	EK-CAL ELISA<br>50µg/g<br>163µg/g<br>325µg/g<br><br>fCal Turbo<br>50µg/g<br>81µg/g	Ileocolonoscopy with histopathology or ‘sum of all findings’	None	136/229 (59%), rest unclear	IBD (excluding microscopic colitis)	IBS

For peer review only - http://bmjopen.bmj.com/site/about/guidelines.xhtml

	86µg/g<br>280µg/g<br><br>Diasorin CLIA<br>43µg/g<br>50µg/g<br>135µg/g					
Dhaliwal 2015 [11];<br>UK	PhiCal ELISA<br>50 and 100µg/g	Endoscopy/histology and/or radiology	None recorded	NR	IBD excluding microscopic colitis	IBS fulfilling the Rome II criteria
El-Badry 2010 [12];<br>Egypt	PhiCal ELISA<br>50 and 100 µg/g	Colonoscopy with biopsies	None recorded	100%	IBD	IBS following Rome II criteria
Garcia 2006 [13];<br>Spain	Calprest<br>217mg/kg	Colonoscopy and histology	None recorded	100%	Organic disease: colon adenomas, CRC, IBD	Other: IBS, diverticulosis, NSAIDs or aspirin, PPI
Hogberg 2017 [14];<br>Sweden	Calpro ELISA<br>20µg/g<br>50µg/g<br>100µg/g	Bowel imaging (colonoscopy, CT colonography, double contrast barium enema) or 2-year follow-up	None	185/384 (48%) imaging, 199/384 (52%) 2-year follow-up	IBD including microscopic colitis and unspecific colitis	No or any other pathology (including CRC, HRA)
Jang 2016 [15];<br>Korea	Ridascreen ELISA<br>Quantum-Blue rapid<br>EliA FEIA<br>50µg/g	Colonoscopy	None recorded	100%	IBD including intestinal Behçet's disease	IBS
Kennedy 2015 [16];<br>UK	PhiCal ELISA<br>20 µg/g<br>50 µg/g<br>70 µg/g<br>100 µg/g	Definitive organic diagnosis, full colonoscopy or minimum of 3 years follow up	None	467/895 (52%) organic diagnosis or colonoscopy, 428/895 (48%) follow up	IBD	Functional
Kok 2012 [17];<br>Netherlands	EK-CAL ELISA<br>Quantum-Blue rapid,<br>50 µg/g	Colonoscopy, sigmoidoscopy (89.9% with biopsy), other bowel examinations, 3 month follow-up	4/423<br>from study	Colonoscopy 351/423 (91.9%), sigmoidoscopy 21/423 (5.5%), other bowel examinations 10/423 (2.6%)<br>With histology in 89.9%<br>Follow-up for inconclusive tests	Organic disease: CRC, adenomas, IBD, and diverticulitis	Other: no structural abnormalities, diverticulosis, IBS, and haemorrhoids
Labaere 2014 [18];<br>Belgium	CalproLab ELISA 50µg/g<br>Quantum-Blue rapid<br>50µg/g and 75µg/g<br>Calfast rapid 70µg/g<br>Calprest ELISA 50µg/g<br>EliA FEIA 15µg/g and 50µg/g<br>CerTest rapid 50µg/g	Ileocolonoscopies + biopsy	1/33excluded from study	100%	IBD	Non-organic conditions + non-IBD organic conditions (nonsteroidal anti-inflammatory drug enteropathy, adenoma, and infectious colitis)
Lee 2014 [19];<br>UK	ELISA<br>60µg/g	Investigation in Gastroenterology clinic	None recorded	Patients with positive FC test	Organic disease: IBD, Diverticulosis, Colonic	Functional

For peer review only - http://bmjopen.bmj.com/site/about/guidelines.xhtml

For peer review only

Abstract					Polyps, Infective colitis and Chronic Pancreatitis	
Li 2006 [20]; China	PhiCal ELISA 50µg/g	Colonoscopy ± biopsy	None recorded	Patients with inflammation, unclear for patients with IBS	Inflammation: IBD, chronic infectious diarrhoea, intestinal tuberculosis, Behcet disease	IBS
Licata 2012 [21]; Italy	Calprest ELISA 150 µg/g	Colonoscopy with biopsy	None recorded	100%	Inflammation: IBD, microscopic colitis, diverticulitis, polyps, ischemic colitis, nonspecific colitis, IBS or undiagnosed microscopic colitis	No inflammation: IBS, diverticulosis
Limburg 2000 [22]; USA	PhiCal ELISA 100µg/g	Colonoscopy and histology	None recorded	100% (n=32 had no biopsy)	Inflammation: CD, UC, microscopic or collagenous colitis, peridiverticulitis, and eosinophilic colitis	normal mucosa, <1cm polyps
Lizvan 2015 [23]; Russia	Quantum-Blue rapid 50µg/g	Colonoscopy	None recorded	100%	Organic disease	IBS
Mowat 2016 [24]; UK	EK-CAL ELISA 50µg/g 200µg/g	Lower endoscopy	163/1031 from analysis	100%	IBD	all non-IBD conditions and normal
Otten 2008 [25]; Netherlands	PhiCal ELISA 50µg/g CalDetect rapid 15µg/g 60µg/g	Colonoscopy or sigmoidoscopy biopsies were taken if necessary	2/144 from study	109 (96%) colonoscopy, and 5 (4%) sigmoidoscopy	IBD	IBS
Oyaert 2014 [26]; Belgium	Quantum-Blue rapid, ELiA ELISA 50µg/g	Ileocolonoscopy and histology	From study, proportion not reported	100%	IBD (excluding indeterminate colitis and microscopic colitis)	IBS, infectious colitis, oesophagitis, erosive gastritis, gastric ulcers, diverticulitis, microscopic colitis, CRC, hyperplastic polyps, adenomatous polyps, coeliac disease, arthritis
Oyaert 2017 [27]; Belgium	EliA ELISA Diasorin CLIA Quanta Flash CLIA fCAL Turbo rapid Euroimmun ELISA Orgentec Alegria ELISA 50 µg/g and test specific optimal cut-off	Ileocolonoscopy and histology	From study, proportion not reported	100%	IBD (excluding microscopic colitis)	oesophagitis, erosive gastritis, gastric ulcers, diverticulitis, microscopic colitis, colorectal cancer, hyperplastic polyps, adenomatous polyps, spondylo-arthritis, (undifferentiated) arthritis, IBS

For peer review only - http://bmjopen.bmj.com/site/about/guidelines.xhtml

For peer review only

Pavlidis 2013 [28]; UK	EK-CAL ELISA 50µg/g 100 µg/g 125 µg/g 150 µg/g 200 µg/g 249 µg/g	Colonoscopy, sigmoidoscopy, other investigations or 1 year follow-up	None	134/962 (14%) colonoscopy, 104/962 (11%) sigmoidoscopy, 130/962 (14%) other, 594/962 (62%) follow-up	Organic disease: IBD, Microscopic colitis, diversion colitis, infective diarrhoea, coeliac, NSAID, Alcohol related, Meckel's diverticulum, Rectal adenocarcinoma	Non-organic
Rosenfeld 2016 [29]; Canada	Quantum-Blue rapid, 100µg/g	Endoscopy, 26-months follow-up	None	8/41 FC negatives and 4/9 FC positives had endoscopy, remainder had 26 months follow up	IBD including microscopic colitis	IBS
Schoepfer 2008 [30]; Switzerland	PhiCal ELISA 50µg/g	Colonoscopy with biopsy	5/187 from study	100%	IBD	IBS according to Rome II criteria
Schroeder 2007 [31]; Germany	IDK ELISA 15µg/ g	Colonoscopy with biopsy	None recorded	100%	IBD	IBS according to Rome II criteria
Sharbatdaran 2018[32] Iran	EK-CAL ELISA 127.65µg/g	Colonoscopy with biopsy	None	100%	IBD	Non-IBD inflammatory bowel diseases, IBS, no diagnosed disease
Shitrit 2007 [33]; Israel	Calprest ELISA 150µg/g	Colonoscopy	None recorded	100%	Organic: IBD, carcinoma, polyps	Normal colonoscopy
Sostres 2017[34]; Spain<br><br>Abstract	EliA FEIA 50µg/g	Colonic investigations (colonoscopy)	None	100%	CRC, advanced adenoma, IBD and angiodysplasia	NR
Tan 2016[35]; UK<br><br>Abstract	NR 50µg/g	Colonoscopies, gastroscopies, MRI of small bowel	None	NR for FC positive FC negatives: 58/95colonoscopy 19/95 gastroscopy, 3/95 MRI of small bowel	IBD	NR
Tibble 2002 [36]; UK	ELISA 10mg/L=50µg/g	Barium enteroclysis/ enema and/or endoscopy/colonoscopy	None recorded	372/602 patients had a full colonoscopy	Crohn's disease, Celiac disease, Infective diarrhea, small bowel enteropathy (NSAID, Alcoholic, Radiation), Diabetic diarrhea, ulcerative colitis, Microscopic colitis, Collagenous colitis, Diverticular disease, cancer	IBS, IBS + nonulcer dyspepsia, IBS + other (lactose intolerance, angiodysplasia, hemorrhoids, and melanosis coli)
Turvill 2012 [37]; UK	PhiCal ELISA 50µg/g	Colonoscopy or barium enema ± histology, or various other investigations	None recorded	FC negative: 43% full evaluation of the colon by colonoscopy or barium enema and 60% had histology.	Organic disease: Crohn's disease, Ulcerative colitis, Indeterminate IBD, Microscopic colitis, NSAID enteropathy, Gastroenteritis, Diverticular	Non-organic: IBS

For peer review only - http://bmjopen.bmj.com/site/about/guidelines.xhtml

				FC positive: 75% underwent full colonic evaluation by colonoscopy or barium enema, supportive histology in 83%	disease, Bacterial overgrowth, Coeliac disease, Postoperative, Upper gastrointestinal bleed, Intussusception, Rectal polyp, Alcoholic enteropathy, Solitary rectal ulcer, Gastrinoma, Cholecystitis, Appendicitis, Bile salt malabsorption, Giardiasis, Chronic pancreatitis, Thyrotoxicosis, Small bowel bacterial overgrowth, Lactose intolerance, Sorbitol induced diarrhoea	
Turvill 2016 [38]; UK	EK-CAL ELISA 100µg/g 250µg/g	Colonoscopy, 6-week review of symptoms by GP or 6 months follow-up, hospital database	None	25% received colonoscopy, remainder received 6-week review of symptoms by GP or follow-up	Organic disease: IBD, non-specific inflammation, microscopic colitis, diverticular disease, gastroenteritis, coeliac disease, pancreatic insufficiency and a low-grade tubulovillous adenoma, haemorrhoids	IBS (incorporated all functional intestinal disease diagnoses) and non-enteric other diagnosis following a normal FC

References

1. Alrubaiy L, Malik A, Rees I, Bowen D. Usefulness of fecal calprotectin in clinical practice in a district general hospital. *Inflammatory Bowel Diseases*. 2012;18:S53-S4.
2. Banerjee A, Srinivas M, Eyre R, Ellis R, Waugh N, Bardhan KD, *et al.* Faecal calprotectin for differentiating between irritable bowel syndrome and inflammatory bowel disease: A useful screen in daily gastroenterology practice. *Frontline Gastroenterology*. 2015;6(1):20-6.
3. Bharathi S, Moncur P, Holbrook I, Kelly S. A normal facael calprotectin has a high negative predictive value in cases of suspected irritable bowel syndrome. *Gastroenterology*. 2005;128(4):A459.
4. Boyd A, Peplow C, Dhaliwal A, O'Rourke J, Davies J, Milestone A. Primary care faecal calprotectin pathway - A three year analysis. *Colorectal Disease*. 2016;18:27.
5. Burri E, Manz M, Rothen C, Rossi L, Beglinger C, Lehmann FS. Monoclonal antibody testing for fecal calprotectin is superior to polyclonal testing of fecal calprotectin and lactoferrin to identify organic intestinal disease in patients with abdominal discomfort. *Clin Chim Acta*. 2013;416:41-7.
6. Carroccio A, Iacono G, Cottone M, Di Prima L, Cartabellotta F, Cavataio F, *et al.* Diagnostic accuracy of fecal calprotectin assay in distinguishing organic causes of chronic diarrhea from irritable bowel syndrome: a prospective study in adults and children. *Clin Chem*. 2003;49(6 Pt 1):861-7.
7. Caviglia GP, Pantaleoni S, Touscoz GA, Adriani A, Rosso C, Smedile A, *et al.* Fecal calprotectin is an effective diagnostic tool that differentiates inflammatory from functional intestinal disorders. *Scandinavian Journal of Gastroenterology*. 2014;49(12):1419-24.
8. Conroy S, Hale MF, Cross SS, Swallow K, Sidhu RH, Sargur R, *et al.* Unrestricted faecal calprotectin testing performs poorly in the diagnosis of inflammatory bowel disease in patients in primary care. *J Clin Pathol*. 2017.

For peer review only - http://bmjopen.bmj.com/site/about/guidelines.xhtml

9. Damms A, Bischoff SC. Validation and clinical significance of a new calprotectin rapid test for the diagnosis of gastrointestinal diseases. *Int J Colorectal Dis*. 2008;23(10):985-92.
10. De Sloovere MM, De Smet D, Baert FJ, Debrabandere J, Vanpoucke HJ. Analytical and diagnostic performance of two automated fecal calprotectin immunoassays for detection of inflammatory bowel disease. *Clinical Chemistry & Laboratory Medicine*. 2017;11:11.
11. Dhaliwal A, Zeino Z, Tomkins C, Cheung M, Nwokolo C, Smith S, *et al.* Utility of faecal calprotectin in inflammatory bowel disease (IBD): what cut-offs should we apply? *Frontline Gastroenterology*. 2015;6(1):14-9.
12. El-Badry A, Sedrak H, Rashed L. Faecal calprotectin in differentiating between functional and organic bowel diseases. *Arab Journal of Gastroenterology*. 2010;11(2):70-3.
13. Garcia Sanchez Mdel V, Gonzalez R, Iglesias Flores E, Gomez Camacho F, Casais Juanena L, Cerezo Ruiz A, *et al.* [Diagnostic value of fecal calprotectin in predicting an abnormal colonoscopy]. *Med Clin (Barc)*. 2006;127(2):41-6. Precision diagnostica de la calprotectina fecal para predecir una colonoscopia patologica.
14. Hogberg C, Karling P, Rutegard J, Lilja M. Diagnosing colorectal cancer and inflammatory bowel disease in primary care: The usefulness of tests for faecal haemoglobin, faecal calprotectin, anaemia and iron deficiency. A prospective study. *Scandinavian Journal of Gastroenterology*. 2017;52(1):69-75.
15. Jang HW, Kim HS, Park SJ, Hong SP, Kim TI, Kim WH, *et al.* Accuracy of three different fecal calprotectin tests in the diagnosis of inflammatory bowel disease. *Intestinal Research*. 2016;14(4):305-13.
16. Kennedy NA, Clark A, Walkden A, Chang JC, Fasci-Spurio F, Muscat M, *et al.* Clinical utility and diagnostic accuracy of faecal calprotectin for IBD at first presentation to gastroenterology services in adults aged 16-50 years. *Journal of Crohn's & colitis*. 2015;9(1):41-9.
17. Kok L, Elias SG, Witteman BJ, Goedhard JG, Muris JW, Moons KG, *et al.* Diagnostic accuracy of point-of-care fecal calprotectin and immunochemical occult blood tests for diagnosis of organic bowel disease in primary care: the Cost-Effectiveness of a Decision Rule for Abdominal Complaints in Primary Care (CEDAR) study. *Clinical Chemistry*. 2012;58(6):989-98.
18. Labaere D, Smismans A, Van Olmen A, Christiaens P, D'Haens G, Moons V, *et al.* Comparison of six different calprotectin assays for the assessment of inflammatory bowel disease. *United European Gastroenterology Journal*. 2014;2(1):30-7.
19. Lee S, Borthwick H, Dhar A. P223. Faecal calprotectin testing in primary and secondary care - Are the current manufacturer's cut-off values clinically useful? 9th Congress of ECCO 2014; Copenhagen: European Crohn's and Colitis Organisation; 2014.
20. Li XG, Lu YM, Gu F, Yang XL. [Fecal calprotectin in differential diagnosis of irritable bowel syndrome]. *Beijing Da Xue Xue Bao*. 2006;38(3):310-3.
21. Licata A, Randazzo C, Cappello M, Calvaruso V, Butera G, Florena AM, *et al.* Fecal calprotectin in clinical practice: a noninvasive screening tool for patients with chronic diarrhea. *J Clin Gastroenterol*. 2012;46(6):504-8.
22. Limburg PJ, Ahlquist DA, Sandborn WJ, Mahoney DW, Devens ME, Harrington JJ, *et al.* Fecal calprotectin levels predict colorectal inflammation among patients with chronic diarrhea referred for colonoscopy. *Am J Gastroenterol*. 2000;95(10):2831-7.
23. Livzan MA, Lyalukova EA, Nechaeva G, Osipenko MF, Dolgih TI. [Fecal Noninvasive Tests (Calprotectin, Transferrin, Hemoglobin) in Complex Diagnosis of Diseases of Intestines]. *Eksperimental'Naia i Klinicheskaia Gastroenterologiia*. 2015(3):34-8.
24. Mowat C, Digby J, Strachan JA, Wilson R, Carey FA, Fraser CG, *et al.* Faecal haemoglobin and faecal calprotectin as indicators of bowel disease in patients presenting to primary care with bowel symptoms. *Gut*. 2016;65(9):1463-9.
25. Otten CM, Kok L, Witteman BJ, Baumgarten R, Kampman E, Moons KG, *et al.* Diagnostic performance of rapid tests for detection of fecal calprotectin and lactoferrin and their ability to discriminate inflammatory from irritable bowel syndrome. *Clin Chem Lab Med*. 2008;46(9):1275-80.
26. Oyaert M, Trouve C, Baert F, De Smet D, Langlois M, Vanpoucke H. Comparison of two immunoassays for measurement of faecal calprotectin in detection of inflammatory bowel disease: (pre)-analytical and diagnostic performance characteristics. *Clinical Chemistry & Laboratory Medicine*. 2014;52(3):391-7.
27. Oyaert M, Boel A, Jacobs J, Van den Bremt S, De Sloovere M, Vanpoucke H, *et al.* Analytical performance and diagnostic accuracy of six different faecal calprotectin assays in inflammatory bowel disease. *Clinical Chemistry & Laboratory Medicine*. 2017;21:21.

For peer review only - http://bmjopen.bmj.com/site/about/guidelines.xhtml

28. Pavlidis P, Chedgy FJ, Tibble JA. Diagnostic accuracy and clinical application of faecal calprotectin in adult patients presenting with gastrointestinal symptoms in primary care. *Scandinavian Journal of Gastroenterology*. 2013;48(9):1048-54.
29. Rosenfeld G, Greenup AJ, Round A, Takach O, Halparin L, Saadeddin A, *et al*. FOCUS: Future of fecal calprotectin utility study in inflammatory bowel disease. *World Journal of Gastroenterology*. 2016;22(36):8211-8.
30. Schoepfer AM, Trummler M, Seeholzer P, Seibold-Schmid B, Seibold F. Discriminating IBD from IBS: comparison of the test performance of fecal markers, blood leukocytes, CRP, and IBD antibodies. *Inflamm Bowel Dis*. 2008;14(1):32-9.
31. Schroder O, Naumann M, Shastri Y, Povse N, Stein J. Prospective evaluation of faecal neutrophil-derived proteins in identifying intestinal inflammation: combination of parameters does not improve diagnostic accuracy of calprotectin. *Aliment Pharmacol Ther*. 2007;26(7):1035-42.
32. Sharbatdaran M, Halakou A, Kashifard M, Bijani A, Firozjaei A, Hossaini A, *et al*. Fecal calprotectin Level in patients with IBD and noninflammatory disease of colon: a study in Babol, Northern, Iran. *Caspian Journal of Internal Medicine*. 2018;9(1):60-4.
33. Shitrit AB, Braverman D, Stankiewics H, Shitrit D, Peled N, Paz K. Fecal calprotectin as a predictor of abnormal colonic histology. *Dis Colon Rectum*. 2007;50(12):2188-93.
34. Sostres C, Lue A, Barra Pardos MV, Hijos G, Perales A, Puente JJ, *et al*. Combination of FOBT and fecal calprotectin may be useful for reducing unnecessary colonoscopies in symptomatic patients. *United European Gastroenterology Journal*. 2017;5 (5 Supplement 1):A779.
35. Tan M, Subramaniam Y, Cheong C, Hasan F, Moran GW, Fateen W, *et al*. The role of faecal calprotectin in a primary care referral pathway in the UK. *United European Gastroenterology Journal*. 2016;4 (5 Supplement 1):A443.
36. Tibble JA, Sigthorsson G, Foster R, Forgacs I, Bjarnason I. Use of surrogate markers of inflammation and Rome criteria to distinguish organic from nonorganic intestinal disease. *Gastroenterology*. 2002;123(2):450-60.
37. Turvill J. High negative predictive value of a normal faecal calprotectin in patients with symptomatic intestinal disease. *Frontline Gastroenterology*. 2012;3(1):21-8.
38. Turvill J, O'Connell S, Brooks A, Bradley-Wood K, Laing J, Thiagarajan S, *et al*. Evaluation of a faecal calprotectin care pathway for use in primary care. *Primary Health Care Research & Development*. 2016;17(5):428-36.

For peer review only - http://bmjopen.bmj.com/site/about/guidelines.xhtml

Supplement 6

Table of risk of bias and applicability concern for each included study

Study	RISK OF BIAS				APPLICABILITY CONCERNS		
	PATIENT SELECTION	INDEX TEST	REFERENCE STANDARD	FLOW AND TIMING	PATIENT SELECTION	INDEX TEST	REFERENCE STANDARD
Alrubaiy 2012	😐	🙂	😐	🙁	😐	🙂	😐
Banerjee 2015	🙂	😐	🙁	🙁	🙁	🙂	🙂
Bharathi 2005	😐	😐	😐	🙁	😐	🙂	😐
Boyd 2016	😐	🙂	🙁	🙁	🙂	🙂	🙁
Burri 2013	😐	🙂	🙁	🙁	🙁	🙂	🙁
Carroccio 2003	🙂	🙂	🙁	🙁	🙁	🙂	🙁
Caviglia 2014	🙁	🙂	🙂	😐	🙁	🙂	🙂
Conroy 2017	🙂	🙂	🙁	🙁	😐	🙂	🙁
Damms 2008	😐	🙂	🙂	🙁	🙁	🙂	🙂
De Sloovere 2017	🙁	🙂	😐	🙁	🙁	🙂	😐
Dhaliwal 2015	😐	😐	😐	🙁	🙁	🙂	😐
El Badry 2010	😐	😐	😐	🙂	🙁	🙂	🙂
Garcia 2006	😐	🙁	😐	🙂	🙁	🙂	🙁
Hogberg 2017	🙂	🙂	🙁	🙁	🙁	🙂	🙂
Jang 2016	🙁	🙂	😐	🙁	🙁	🙂	😐
Kennedy 2015	🙂	😐	🙁	🙁	🙁	🙂	🙁
Kok 2012	😐	🙂	🙂	🙁	🙁	🙂	🙁
Labaere 2014	😐	🙁	🙂	🙂	🙁	🙂	🙂
Lee 2014	😐	😐	😐	😐	🙁	🙂	🙁
Li 2006	🙁	😐	😐	😐	🙁	🙂	😐
Licata 2012	🙂	🙁	🙂	😐	🙁	🙂	🙂
Limburg 2000	😐	🙂	🙁	🙁	🙁	🙂	🙁
Lizvan 2015	😐	😐	😐	😐	😐	😐	🙁
Mowat 2016	🙁	🙂	😐	🙁	🙁	🙂	😐
Otten 2008	🙁	🙂	🙁	🙁	🙁	🙂	🙁
Oyaert 2014	🙁	😐	🙂	😐	🙁	🙂	🙂
Oyaert 2017	🙁	🙁	🙂	😐	🙁	🙂	🙂
Pavlidis 2013	😐	🙂	🙁	🙁	🙂	🙂	🙁
Rosenfeld 2016	😐	🙂	🙁	🙁	😐	🙂	😐
Schoepfer 2008	🙁	🙂	🙂	🙂	🙁	🙂	🙂
Schroeder 2007	🙂	🙂	🙂	🙂	🙁	🙂	🙂
Sharbatdaran 2018	🙁	🙁	😐	🙁	🙁	😐	🙂
Shitrit 2007	😐	🙁	😐	😐	🙁	🙂	🙁
Sostres 2017	😐	😐	😐	😐	🙁	😐	🙁

For peer review only

Tan 2016	😐	☺	☹	☹	😐	☺	☹
Tibble 2002	😐	☹	☹	☹	☹	☺	☹
Turvill 2012	😐	😐	☹	☹	☹	☺	☹
Turvill 2016	☺	☺	☹	☹	☺	☺	☹

☹ high 😐 unclear ☺ low

For peer review only

Supplement 7 Paired forest plots of sensitivity and specificity of 38 included studies by clinical question (Letters following the year indicate different 2x2 table data from the same study reference)

Study	TP	FP	FN	TN	test type	Clin question	cut-off in µg/g	Setting	test name	Sensitivity (95% CI)	Specificity (95% CI)
Banerjee 2015 a	12	48	0	50	ELISA	IBD vs IBS	8.0	secondary	IDK	1.00 [0.74, 1.00]	0.51 [0.41, 0.61]
Otten 2008b	23	5	0	86	POCT	IBD vs IBS	15.0	secondary	CalDetect	1.00 [0.85, 1.00]	0.95 [0.88, 0.98]
Schroeder 2007	42	0	3	31	ELISA	IBD vs IBS	15.0	secondary	IDK	0.93 [0.82, 0.99]	1.00 [0.89, 1.00]
Kennedy 2015a	90	289	1	277	ELISA	IBD vs functional	20.0	secondary	PhiCal	0.99 [0.94, 1.00]	0.49 [0.45, 0.53]
Banerjee 2015 b	12	46	0	52	ELISA	IBD vs IBS	25.0	secondary	IDK	1.00 [0.74, 1.00]	0.53 [0.43, 0.63]
De Sloovere 2017i	45	15	1	101	CLIA	IBD vs IBS	43.0	secondary	Diasorin	0.98 [0.88, 1.00]	0.87 [0.80, 0.93]
Carroccio 2003a	11	8	0	32	ELISA	IBD vs IBS	50.0	secondary	Calprest	1.00 [0.72, 1.00]	0.80 [0.64, 0.91]
Caviglia 2014 a	24	10	0	11	ELISA	IBD vs IBS	50.0	secondary	Calprest	1.00 [0.86, 1.00]	0.52 [0.30, 0.74]
De Sloovere 2017h	43	14	3	102	CLIA	IBD vs IBS	50.0	secondary	Diasorin	0.93 [0.82, 0.99]	0.88 [0.81, 0.93]
De Sloovere 2017a	46	55	0	61	ELISA	IBD vs IBS	50.0	secondary	EK-CAL	1.00 [0.92, 1.00]	0.53 [0.43, 0.62]
Damms 2008a	18	12	0	44	ELISA	IBD vs functional	50.0	secondary	EK-CAL	1.00 [0.81, 1.00]	0.79 [0.66, 0.88]
Jang 2016a	30	1	1	4	FEIA	IBD vs IBS	50.0	secondary	EliA	0.97 [0.83, 1.00]	0.80 [0.28, 0.99]
Banerjee 2015 c	12	39	0	59	ELISA	IBD vs IBS	50.0	secondary	IDK	1.00 [0.74, 1.00]	0.60 [0.50, 0.70]
Dhaliwal 2015	126	33	18	115	ELISA	IBD vs IBS	50.0	secondary	PhiCal	0.88 [0.81, 0.92]	0.78 [0.70, 0.84]
El-Badry 2010a	9	3	0	17	ELISA	IBD vs IBS	50.0	secondary	PhiCal	1.00 [0.66, 1.00]	0.85 [0.62, 0.97]
Otten 2008a	22	12	1	79	ELISA	IBD vs IBS	50.0	secondary	PhiCal	0.96 [0.78, 1.00]	0.87 [0.78, 0.93]
Schoepfer 2008	53	0	11	30	ELISA	IBD vs IBS	50.0	secondary	PhiCal	0.83 [0.71, 0.91]	1.00 [0.88, 1.00]
Kennedy 2015b	88	149	3	417	ELISA	IBD vs functional	50.0	secondary	PhiCal	0.97 [0.91, 0.99]	0.74 [0.70, 0.77]
Damms 2008b	16	11	2	45	POCT	IBD vs functional	50.0	secondary	Prevista	0.89 [0.65, 0.99]	0.80 [0.68, 0.90]
Jang 2016b	31	1	0	4	POCT	IBD vs IBS	50.0	secondary	Quantum-Blue	1.00 [0.89, 1.00]	0.80 [0.28, 0.99]
Jang 2016c	25	0	6	5	ELISA	IBD vs IBS	50.0	secondary	Ridascreen	0.81 [0.63, 0.93]	1.00 [0.48, 1.00]
De Sloovere 2017d	46	30	0	86	PETIA	IBD vs IBS	50.0	secondary	fCal Turbo	1.00 [0.92, 1.00]	0.74 [0.65, 0.82]
Otten 2008c	14	2	9	89	POCT	IBD vs IBS	60.0	secondary	CalDetect	0.61 [0.39, 0.80]	0.98 [0.92, 1.00]
Bharathi 2005	0	12	0	46	ELISA	IBD vs IBS	60.0	secondary	PhiCal	Not estimable	0.79 [0.67, 0.89]
Kennedy 2015c	88	112	3	454	ELISA	IBD vs functional	70.0	secondary	PhiCal	0.97 [0.91, 0.99]	0.80 [0.77, 0.83]
Banerjee 2015 d	11	25	1	73	ELISA	IBD vs IBS	75.0	secondary	IDK	0.92 [0.62, 1.00]	0.74 [0.65, 0.83]
De Sloovere 2017e	46	16	0	100	PETIA	IBD vs IBS	81.0	secondary	fCal Turbo	1.00 [0.92, 1.00]	0.86 [0.79, 0.92]
De Sloovere 2017f	45	15	1	101	PETIA	IBD vs IBS	86.0	secondary	fCal Turbo	0.98 [0.88, 1.00]	0.87 [0.80, 0.93]
Caviglia 2014 b	21	6	3	15	ELISA	IBD vs IBS	100.0	secondary	Calprest	0.88 [0.68, 0.97]	0.71 [0.48, 0.89]
Banerjee 2015 e	11	18	1	80	ELISA	IBD vs IBS	100.0	secondary	IDK	0.92 [0.62, 1.00]	0.82 [0.73, 0.89]
El-Badry 2010b	7	0	2	20	ELISA	IBD vs IBS	100.0	secondary	PhiCal	0.78 [0.40, 0.97]	1.00 [0.83, 1.00]
Kennedy 2015d	87	74	4	492	ELISA	IBD vs functional	100.0	secondary	PhiCal	0.96 [0.89, 0.99]	0.87 [0.84, 0.90]
Rosenfeld 2016	4	5	0	41	POCT	IBD vs IBS	100.0	secondary	Quantum-Blue	1.00 [0.40, 1.00]	0.89 [0.76, 0.96]
De Sloovere 2017j	36	2	10	114	CLIA	IBD vs IBS	135.0	secondary	Diasorin	0.78 [0.64, 0.89]	0.98 [0.94, 1.00]
Caviglia 2014 c	21	2	3	19	ELISA	IBD vs IBS	150.0	secondary	Calprest	0.88 [0.68, 0.97]	0.90 [0.70, 0.99]
Banerjee 2015 f	10	14	2	84	ELISA	IBD vs IBS	150.0	secondary	IDK	0.83 [0.52, 0.98]	0.86 [0.77, 0.92]
De Sloovere 2017b	45	11	1	105	ELISA	IBD vs IBS	163.0	secondary	EK-CAL	0.98 [0.88, 1.00]	0.91 [0.84, 0.95]
De Sloovere 2017g	37	4	9	112	PETIA	IBD vs IBS	280.0	secondary	fCal Turbo	0.80 [0.66, 0.91]	0.97 [0.91, 0.99]
De Sloovere 2017c	38	4	8	112	ELISA	IBD vs IBS	325.0	secondary	EK-CAL	0.83 [0.69, 0.92]	0.97 [0.91, 0.99]

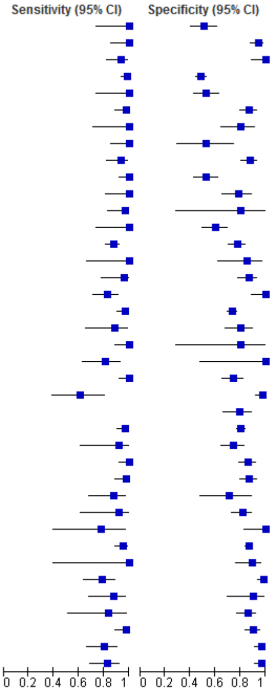

Forest plots of studies reporting data for clinical questions IBD vs IBS and IBD vs functional disease (14 studies)

For peer review only - http://bmjopen.bmj.com/site/about/guidelines.xhtml

Study	TP	FP	FN	TN	test type	Clin question	cut-off in µg/g	Setting	test name	Sensitivity (95% CI)	Specificity (95% CI)
Hogberg 2017 a	10	175	0	188	ELISA	IBD vs non-IBD	20.0	primary	Calpro	1.00 [0.69, 1.00]	0.52 [0.47, 0.57]
Conroy 2017a	8	140	3	259	ELISA	IBD vs non-IBD	50.0	primary	IDK	0.73 [0.39, 0.94]	0.65 [0.60, 0.70]
Boyd 2016 a	23	117	0	284	unclear	IBD vs non-IBD	50.0	primary	NR	1.00 [0.85, 1.00]	0.71 [0.66, 0.75]
Hogberg 2017 b	9	100	1	263	ELISA	IBD vs non-IBD	50.0	primary	Calpro	0.90 [0.55, 1.00]	0.72 [0.68, 0.77]
Conroy 2017b	6	78	5	321	ELISA	IBD vs non-IBD	100.0	primary	IDK	0.55 [0.23, 0.83]	0.80 [0.76, 0.84]
Hogberg 2017 c	7	51	3	312	ELISA	IBD vs non-IBD	100.0	primary	Calpro	0.70 [0.35, 0.93]	0.86 [0.82, 0.89]
Conroy 2017c	6	66	5	333	ELISA	IBD vs non-IBD	125.0	primary	IDK	0.55 [0.23, 0.83]	0.83 [0.79, 0.87]
Conroy 2017d	5	53	6	346	ELISA	IBD vs non-IBD	150.0	primary	IDK	0.45 [0.17, 0.77]	0.87 [0.83, 0.90]
Boyd 2016 b	21	44	2	357	unclear	IBD vs non-IBD	150.0	primary	NR	0.91 [0.72, 0.99]	0.89 [0.86, 0.92]
Conroy 2017e	3	26	8	373	ELISA	IBD vs non-IBD	250.0	primary	IDK	0.27 [0.06, 0.61]	0.93 [0.91, 0.96]
Labaere 2014a	10	3	2	16	FEIA	IBD vs non-IBD	15.0	secondary	EliA	0.83 [0.52, 0.98]	0.84 [0.60, 0.97]
De Sloovere 2017s	39	34	1	62	CLIA	IBD vs non-IBD	34.0	secondary	Diasorin	0.97 [0.87, 1.00]	0.65 [0.54, 0.74]
De Sloovere 2017k	40	54	0	42	ELISA	IBD vs non-IBD	50.0	secondary	EK-CAL	1.00 [0.91, 1.00]	0.44 [0.34, 0.54]
Carroccio 2003b	11	16	0	43	ELISA	IBD vs non-IBD	50.0	secondary	Calprest	1.00 [0.72, 1.00]	0.73 [0.60, 0.84]
Oyaert 2017i	21	27	0	38	ELISA	IBD vs non-IBD	50.0	secondary	Euroimmun	1.00 [0.84, 1.00]	0.58 [0.46, 0.71]
Oyaert 2017g	21	27	0	38	ELISA	IBD vs non-IBD	50.0	secondary	Orgentec	1.00 [0.84, 1.00]	0.58 [0.46, 0.71]
Oyaert 2017e	21	14	0	51	CLIA	IBD vs non-IBD	50.0	secondary	Diasorin	1.00 [0.84, 1.00]	0.78 [0.67, 0.88]
Oyaert 2017c	21	18	0	47	CLIA	IBD vs non-IBD	50.0	secondary	Quanta Flash	1.00 [0.84, 1.00]	0.72 [0.60, 0.83]
Oyaert 2017k	21	22	0	43	PETIA	IBD vs non-IBD	50.0	secondary	fCal Turbo	1.00 [0.84, 1.00]	0.66 [0.53, 0.77]
De Sloovere 2017o	40	45	0	51	PETIA	IBD vs non-IBD	50.0	secondary	fCal Turbo	1.00 [0.91, 1.00]	0.53 [0.43, 0.63]
De Sloovere 2017t	38	33	2	63	CLIA	IBD vs non-IBD	50.0	secondary	Diasorin	0.95 [0.83, 0.99]	0.66 [0.55, 0.75]
El-Badry 2010c	9	13	0	17	ELISA	IBD vs non-IBD	50.0	secondary	PhiCal	1.00 [0.66, 1.00]	0.57 [0.37, 0.75]
Labaere 2014e	10	6	2	13	POCT	IBD vs non-IBD	50.0	secondary	Quantum-Blue	0.83 [0.52, 0.98]	0.68 [0.43, 0.87]
Labaere 2014g	10	3	2	16	POCT	IBD vs non-IBD	50.0	secondary	CerTest	0.83 [0.52, 0.98]	0.84 [0.60, 0.97]
Labaere 2014b	9	1	3	18	FEIA	IBD vs non-IBD	50.0	secondary	EliA	0.75 [0.43, 0.95]	0.95 [0.74, 1.00]
Labaere 2014c	9	2	2	15	ELISA	IBD vs non-IBD	50.0	secondary	Calprest	0.82 [0.48, 0.98]	0.88 [0.64, 0.99]
Labaere 2014d	10	2	2	17	FEIA	IBD vs non-IBD	50.0	secondary	CalproLab	0.83 [0.52, 0.98]	0.89 [0.67, 0.99]
Oyaert 2014a	48	17	3	115	FEIA	IBD vs non-IBD	50.0	secondary	EliA	0.94 [0.84, 0.99]	0.87 [0.80, 0.92]
Tan 2016	15	165	0	95	unclear	IBD vs non-IBD	50.0	secondary	NR	1.00 [0.78, 1.00]	0.37 [0.31, 0.43]
Oyaert 2014b	51	37	0	95	POCT	IBD vs non-IBD	50.0	secondary	Quantum-Blue	1.00 [0.93, 1.00]	0.72 [0.63, 0.79]
Oyaert 2017a	21	22	0	43	FEIA	IBD vs non-IBD	50.0	secondary	EliA	1.00 [0.84, 1.00]	0.66 [0.53, 0.77]
Mowat 2016a	29	421	3	273	ELISA	IBD vs non-IBD	50.0	secondary	EK-CAL	0.91 [0.75, 0.98]	0.39 [0.36, 0.43]
Alrubaiy 2012	2	18	0	34	unclear	Infl vs non-infl	50.0	secondary	NR	1.00 [0.16, 1.00]	0.65 [0.51, 0.78]
Caviglia 2014d	38	10	7	11	ELISA	Infl vs non-infl	50.0	secondary	Calprest	0.84 [0.71, 0.94]	0.52 [0.30, 0.74]
Li 2006	55	6	5	54	ELISA	Infl vs non-infl	50.0	secondary	PhiCal	0.92 [0.82, 0.97]	0.90 [0.79, 0.96]
Labaere 2014h	9	2	2	15	POCT	IBD vs non-IBD	70.0	secondary	Calfast	0.82 [0.48, 0.98]	0.88 [0.64, 0.99]
Labaere 2014f	10	3	2	16	POCT	IBD vs non-IBD	75.0	secondary	Quantum-Blue	0.83 [0.52, 0.98]	0.84 [0.60, 0.97]
De Sloovere 2017u	36	20	4	76	CLIA	IBD vs non-IBD	78.0	secondary	Diasorin	0.90 [0.76, 0.97]	0.79 [0.70, 0.87]
De Sloovere 2017p	39	38	1	58	PETIA	IBD vs non-IBD	83.0	secondary	fCal Turbo	0.97 [0.87, 1.00]	0.60 [0.50, 0.70]
El-Badry 2010d	7	8	2	22	ELISA	IBD vs non-IBD	100.0	secondary	PhiCal	0.78 [0.40, 0.97]	0.73 [0.54, 0.88]
Caviglia 2014e	31	6	14	15	ELISA	Infl vs non-infl	100.0	secondary	Calprest	0.69 [0.53, 0.82]	0.71 [0.48, 0.89]
Limburg 2000	24	14	5	67	ELISA	Infl vs non-infl	100.0	secondary	PhiCal	0.83 [0.64, 0.94]	0.83 [0.73, 0.90]
Oyaert 2017f	21	2	0	63	CLIA	IBD vs non-IBD	111.0	secondary	Diasorin	1.00 [0.84, 1.00]	0.97 [0.89, 1.00]
Oyaert 2017d	21	9	0	56	CLIA	IBD vs non-IBD	115.0	secondary	Quanta Flash	1.00 [0.84, 1.00]	0.86 [0.75, 0.93]
Sharbatdaran 2018	33	3	12	40	ELISA	IBD vs non-IBD	127.65	secondary	EK-CAL	0.73 [0.58, 0.85]	0.93 [0.81, 0.99]
Caviglia 2014f	30	2	15	19	ELISA	Infl vs non-infl	150.0	secondary	Calprest	0.67 [0.51, 0.80]	0.90 [0.70, 0.99]
Licata 2012	107	24	35	180	ELISA	Infl vs non-infl	150.0	secondary	Calprest	0.75 [0.67, 0.82]	0.88 [0.83, 0.92]
De Sloovere 2017l	39	32	1	64	ELISA	IBD vs non-IBD	164.0	secondary	EK-CAL	0.97 [0.87, 1.00]	0.67 [0.56, 0.76]
Mowat 2016b	23	165	9	529	ELISA	IBD vs non-IBD	200.0	secondary	EK-CAL	0.72 [0.53, 0.86]	0.76 [0.73, 0.79]
De Sloovere 2017v	30	7	10	89	CLIA	IBD vs non-IBD	211.0	secondary	Diasorin	0.75 [0.59, 0.87]	0.93 [0.86, 0.97]
De Sloovere 2017q	35	20	5	76	PETIA	IBD vs non-IBD	243.0	secondary	fCal Turbo	0.88 [0.73, 0.96]	0.79 [0.70, 0.87]
Oyaert 2017l	21	3	0	62	PETIA	IBD vs non-IBD	285.0	secondary	fCal Turbo	1.00 [0.84, 1.00]	0.95 [0.87, 0.99]
De Sloovere 2017m	36	21	4	57	ELISA	IBD vs non-IBD	311.0	secondary	EK-CAL	0.90 [0.76, 0.97]	0.73 [0.62, 0.82]
De Sloovere 2017r	29	9	11	87	PETIA	IBD vs non-IBD	358.0	secondary	fCal Turbo	0.72 [0.56, 0.85]	0.91 [0.83, 0.96]
Oyaert 2017j	21	2	0	63	ELISA	IBD vs non-IBD	371.0	secondary	Euroimmun	1.00 [0.84, 1.00]	0.97 [0.89, 1.00]
Oyaert 2017b	18	0	3	65	FEIA	IBD vs non-IBD	376.0	secondary	EliA	0.86 [0.64, 0.97]	1.00 [0.94, 1.00]
Oyaert 2017h	21	1	0	46	ELISA	IBD vs non-IBD	477.0	secondary	Orgentec	1.00 [0.84, 1.00]	0.98 [0.89, 1.00]
De Sloovere 2017n	28	11	12	85	ELISA	IBD vs non-IBD	919.0	secondary	EK-CAL	0.70 [0.53, 0.83]	0.89 [0.80, 0.94]

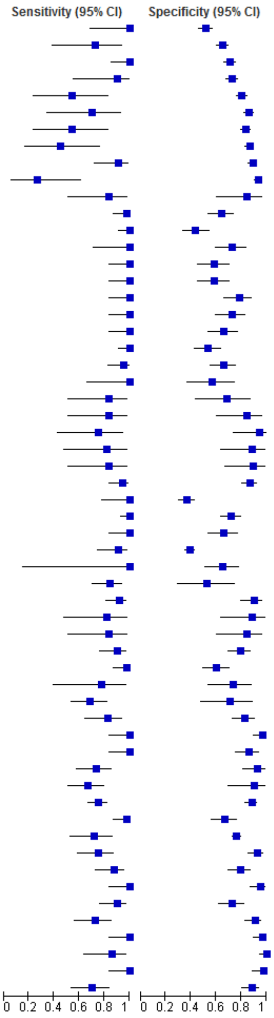

Forest plots of studies reporting data for clinical questions IBD vs non-IBD and Inflammatory vs non-inflammatory intestinal disease (16 studies)

For peer review only - http://bmjopen.bmj.com/site/about/guidelines.xhtml

Study	TP	FP	FN	TN	test type	Clin question	cut-off in µg/g	Setting	test name	Sensitivity (95% CI)	Specificity (95% CI)
Lee 2013	19	32	4	67	ELISA	organic vs non-organic	60.0	mix	NR	0.83 [0.61, 0.95]	0.68 [0.58, 0.77]
Hogberg 2017d	25	160	1	187	ELISA	organic vs other	20.0	primary	Calpro	0.96 [0.80, 1.00]	0.54 [0.48, 0.59]
Hogberg 2017e	20	89	6	258	ELISA	organic vs other	50.0	primary	Calpro	0.77 [0.56, 0.91]	0.74 [0.69, 0.79]
Pavlidis 2013a	77	199	17	669	ELISA	organic vs non-organic	50.0	primary	EK-CAL	0.82 [0.73, 0.89]	0.77 [0.74, 0.80]
Conroy 2017f	21	127	6	256	ELISA	organic vs non-organic	50.0	primary	IDK	0.78 [0.58, 0.91]	0.67 [0.62, 0.72]
Hogberg 2017f	12	46	14	301	ELISA	organic vs other	100.0	primary	Calpro	0.46 [0.27, 0.67]	0.87 [0.83, 0.90]
Pavlidis 2013b	73	78	21	790	ELISA	organic vs non-organic	100.0	primary	EK-CAL	0.78 [0.68, 0.86]	0.91 [0.89, 0.93]
Turvill 2016a	20	44	6	192	ELISA	organic vs non-organic	100.0	primary	EK-CAL	0.77 [0.56, 0.91]	0.81 [0.76, 0.86]
Pavlidis 2013c	67	43	27	825	ELISA	organic vs non-organic	125.0	primary	EK-CAL	0.71 [0.61, 0.80]	0.95 [0.93, 0.96]
Pavlidis 2013d	65	26	29	842	ELISA	organic vs non-organic	150.0	primary	EK-CAL	0.69 [0.59, 0.78]	0.97 [0.96, 0.98]
Pavlidis 2013e	60	9	34	859	ELISA	organic vs non-organic	200.0	primary	EK-CAL	0.64 [0.53, 0.73]	0.99 [0.98, 1.00]
Pavlidis 2013f	54	9	40	859	ELISA	organic vs non-organic	249.0	primary	EK-CAL	0.57 [0.47, 0.68]	0.99 [0.98, 1.00]
Turvill 2016b	16	14	10	222	ELISA	organic vs non-organic	250.0	primary	EK-CAL	0.62 [0.41, 0.80]	0.94 [0.90, 0.97]
Banerjee 2015g	21	48	0	50	ELISA	organic vs non-organic	8.0	secondary	IDK	1.00 [0.84, 1.00]	0.51 [0.41, 0.61]
Kennedy 2015e	139	289	17	277	ELISA	organic vs non-organic	20.0	secondary	PhiCal	0.89 [0.83, 0.94]	0.49 [0.45, 0.53]
Burri 2013c	84	16	43	218	ELISA	organic vs other	22.4	secondary	PhiCal	0.66 [0.57, 0.74]	0.93 [0.89, 0.96]
Banerjee 2015h	20	46	1	52	ELISA	organic vs non-organic	25.0	secondary	IDK	0.95 [0.76, 1.00]	0.53 [0.43, 0.63]
Carroccio 2003c	19	8	11	32	ELISA	organic vs non-organic	50.0	secondary	Calprest	0.63 [0.44, 0.80]	0.80 [0.64, 0.91]
Burri 2013a	111	13	32	249	ELISA	organic vs other	50.0	secondary	EK-CAL	0.78 [0.70, 0.84]	0.95 [0.92, 0.97]
Damms 2008c	52	13	14	43	ELISA	organic vs non-organic	50.0	secondary	EK-CAL	0.79 [0.67, 0.88]	0.77 [0.64, 0.87]
Kok 2012a	73	149	25	132	ELISA	organic vs non-organic	50.0	secondary	EK-CAL	0.74 [0.65, 0.83]	0.47 [0.41, 0.53]
Mowat 2016c	76	374	25	251	ELISA	organic vs non-organic	50.0	secondary	EK-CAL	0.75 [0.66, 0.83]	0.40 [0.36, 0.44]
Sostres 2017	27	63	10	71	FEIA	organic vs non-organic	50.0	secondary	EliA	0.73 [0.56, 0.86]	0.53 [0.44, 0.62]
Banerjee 2015i	19	39	2	59	ELISA	organic vs non-organic	50.0	secondary	IDK	0.90 [0.70, 0.99]	0.60 [0.50, 0.70]
Tibble 2002	234	71	29	268	ELISA	organic vs non-organic	50.0	secondary	NR	0.89 [0.85, 0.92]	0.79 [0.74, 0.83]
Burri 2013b	55	4	72	230	ELISA	organic vs other	50.0	secondary	PhiCal	0.43 [0.35, 0.52]	0.98 [0.96, 1.00]
El-Badry 2010e	19	3	0	17	ELISA	organic vs non-organic	50.0	secondary	PhiCal	1.00 [0.82, 1.00]	0.85 [0.62, 0.97]
Kennedy 2015f	123	149	33	417	ELISA	organic vs non-organic	50.0	secondary	PhiCal	0.79 [0.72, 0.85]	0.74 [0.70, 0.77]
Turvill 2012	91	39	18	482	ELISA	organic vs non-organic	50.0	secondary	PhiCal	0.83 [0.75, 0.90]	0.93 [0.90, 0.95]
Damms 2008d	53	16	13	40	POCT	organic vs non-organic	50.0	secondary	Prevista	0.80 [0.69, 0.89]	0.71 [0.58, 0.83]
Kok 2012b	63	131	36	150	POCT	organic vs non-organic	50.0	secondary	Quantum-Blue	0.64 [0.53, 0.73]	0.53 [0.47, 0.59]
Lizvan 2015	32	1	4	15	POCT	organic vs non-organic	50.0	secondary	Quantum-Blue	0.89 [0.74, 0.97]	0.94 [0.70, 1.00]
Kennedy 2015g	114	112	42	454	ELISA	organic vs non-organic	70.0	secondary	PhiCal	0.73 [0.65, 0.80]	0.80 [0.77, 0.83]
Banerjee 2015j	15	25	6	73	ELISA	organic vs non-organic	75.0	secondary	IDK	0.71 [0.48, 0.89]	0.74 [0.65, 0.83]
Carroccio 2003d	13	3	17	37	ELISA	organic vs non-organic	100.0	secondary	Calprest	0.43 [0.25, 0.63]	0.93 [0.80, 0.98]
Banerjee 2015k	14	18	7	80	ELISA	organic vs non-organic	100.0	secondary	IDK	0.67 [0.43, 0.85]	0.82 [0.73, 0.89]
El-Badry 2010f	15	0	4	20	ELISA	organic vs non-organic	100.0	secondary	PhiCal	0.79 [0.54, 0.94]	1.00 [0.83, 1.00]
Kennedy 2015h	109	74	47	492	ELISA	organic vs non-organic	100.0	secondary	PhiCal	0.70 [0.62, 0.77]	0.87 [0.84, 0.90]
Shitrit 2007	21	5	7	39	ELISA	organic vs non-organic	150.0	secondary	Calprest	0.75 [0.55, 0.89]	0.89 [0.75, 0.96]
Banerjee 2015l	13	14	8	84	ELISA	organic vs non-organic	150.0	secondary	IDK	0.62 [0.38, 0.82]	0.86 [0.77, 0.92]
Mowat 2016d	44	144	57	481	ELISA	organic vs non-organic	200.0	secondary	EK-CAL	0.44 [0.34, 0.54]	0.77 [0.73, 0.80]
Garcia 2006	62	22	11	95	ELISA	organic vs other	217.0	secondary	Calprest	0.85 [0.75, 0.92]	0.81 [0.73, 0.88]

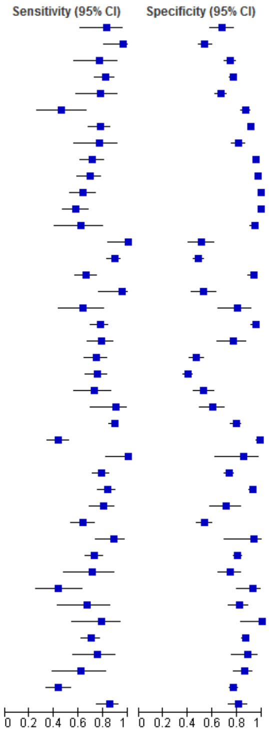

Forest plots of studies reporting data for clinical questions organic intestinal disease vs non-organic intestinal disease and organic intestinal disease vs other intestinal conditions (19 studies)

For peer review only

Supplement 8 2x2 table data of studies included in meta-analysis by test (50µg/g threshold) (Letters following the year indicate different 2x2 table data from the same study reference)

EK-CAL (6 studies)

Study ID	Year of study	TP	FP	TN	FN	Test type	Test name
Burri 2013a	2013	111	13	249	32	ELISA	EK-CAL
Damms 2008c	2008	52	13	43	14	ELISA	EK-CAL
De Sloovere 2017a	2017	46	55	61	0	ELISA	EK-CAL
Kok 2012a	2012	73	149	132	25	ELISA	EK-CAL
Mowat 2016 a	2016	29	421	273	3	ELISA	EK-CAL
Pavlidis 2013a	2013	77	199	669	17	ELISA	EK-CAL

ELiA (5 studies)

Study ID	Year of study	TP	FP	TN	FN	Test type	Test name
Jang 2016a	2016	30	1	4	1	FEIA	ELiA
Labaere 2014b	2014	9	1	18	3	FEIA	ELiA
Oyaert 2014a	2014	48	17	115	3	FEIA	ELiA
Oyaert 2017a	2017	21	22	43	0	FEIA	ELiA
Sostres 2017	2017	27	63	71	10	FEIA	ELiA

PhiCal (8 studies)

Study ID	Year of study	TP	FP	TN	FN	Test type	Test name
Burri 2013b	2013	55	4	230	72	ELISA	PhiCal
Dhaliwal 2015	2015	126	33	115	18	ELISA	PhiCal
El-Badry 2010c	2010	9	13	17	0	ELISA	PhiCal
Kennedy 2015b	2015	88	149	417	3	ELISA	PhiCal
Li 2006	2006	55	6	54	5	ELISA	PhiCal
Otten 2008a	2008	22	12	79	1	ELISA	PhiCal
Schoepfer 2008	2008	53	0	30	11	ELISA	PhiCal
Turvill 2012	2012	91	39	482	18	ELISA	PhiCal

Quantum-Blue (5 studies)

Study ID	Year of study	TP	FP	TN	FN	Test type	Test name
Jang 2016b	2016	31	1	4	0	POCT	Quantum-Blue
Kok 2012b	2012	63	131	150	36	POCT	Quantum-Blue
Labaere 2014e	2014	10	6	13	2	POCT	Quantum-Blue
Lizvan 2015	2015	32	1	15	4	POCT	Quantum-Blue
Oyaert 2014b	2014	51	37	95	0	POCT	Quantum-Blue

For peer review only

Supplement 9 2x2 table data of studies included in meta-analysis by clinical question (50μg/g threshold)

IBD versus IBS (11 studies)

Study ID	Year of study	TP	FP	TN	FN	Clinical question
Banerjee 2015	2015	12	39	59	0	IBD vs IBS
Carroccio 2003	2003	11	8	32	0	IBD vs IBS
Caviglia 2014	2014	24	10	11	0	IBD vs IBS
Damms 2008	2008	18	12	44	0	IBD vs functional
De Sloovere 2017	2017	46	55	61	0	IBD vs IBS
Dhaliwal 2015	2015	126	33	115	18	IBD vs IBS
El-Badry 2010	2010	9	3	17	0	IBD vs IBS
Jang 2016	2016	30	1	4	1	IBD vs IBS
Kennedy 2015	2015	88	149	417	3	IBD vs functional
Otten 2008	2008	22	12	79	1	IBD vs IBS
Schoepfer 2008	2008	53	0	30	11	IBD vs IBS

IBD versus non-IBD (14 studies)

Study ID	Year of study	TP	FP	TN	FN	Clinical question
Alrubaiy 2012	2012	2	18	34	0	Infl vs non-infl
Boyd 2016	2016	23	117	284	0	IBD vs non-IBD
Carroccio 2003	2003	11	16	43	0	IBD vs non-IBD
Caviglia 2014	2014	38	10	11	7	Infl vs non-infl
Conroy 2017	2017	8	140	259	3	IBD vs non-IBD
De Sloovere 2017	2017	38	33	63	2	IBD vs non-IBD
El-Badry 2010	2010	9	13	17	0	IBD vs non-IBD
Hogberg 2017	2017	9	100	263	1	IBD vs non-IBD
Labaere 2014	2014	9	1	18	3	IBD vs non-IBD
Li 2006	2006	55	6	54	5	Infl vs non-infl
Mowat 2016	2016	29	421	273	3	IBD vs non-IBD
Oyaert 2014	2014	51	37	95	0	IBD vs non-IBD
Oyaert 2017	2017	21	18	47	0	IBD vs non-IBD
Tan 2016	2016	15	165	95	0	IBD vs non-IBD

Organic versus non-organic (15 studies)

Study ID	Year of study	TP	FP	TN	FN	Clinical question
Banerjee 2015	2015	19	39	59	2	organic vs non-organic
Burri 2013	2013	111	13	249	32	organic vs other
Carroccio 2003	2003	19	8	32	11	organic vs non-organic
Conroy 2017	2017	21	127	256	6	organic vs non-organic
Damms 2008	2008	53	16	40	13	organic vs non-organic
El-Badry 2010	2010	19	3	17	0	organic vs non-organic
Hogberg 2017	2017	20	89	258	6	organic vs other
Kennedy 2015	2015	123	149	417	33	organic vs non-organic
Kok 2012	2012	63	131	150	36	organic vs non-organic
Lizvan 2015	2015	32	1	15	4	organic vs non-organic
Mowat 2016	2016	76	374	251	25	organic vs non-organic

For peer review only - http://bmjopen.bmj.com/site/about/guidelines.xhtml

Pavlidis 2013	2013	77	199	669	17	organic vs non-organic
Sostres 2017	2017	27	63	71	10	organic vs non-organic
Tibble 2002	2002	234	71	268	29	organic vs non-organic
Turvill 2012	2012	91	39	482	18	organic vs non-organic

Supplement 10 2x2 table data at 100μg/g threshold by clinical question

IBD versus IBS (5 studies)

Study ID	Year of study	TP	FP	TN	FN	Clinical question	Threshold μg/g
Banerjee 2015	2015	11	18	80	1	IBD vs IBS	100
Caviglia 2014	2014	21	6	15	3	IBD vs IBS	100
El-Badry 2010	2010	7	0	20	2	IBD vs IBS	100
Kennedy 2015	2015	87	74	492	4	IBD vs functional	100
Rosenfeld 2016	2016	4	5	41	0	IBD vs IBS	100

IBD versus non-IBD (5 studies)

Study ID	Year of study	TP	FP	TN	FN	Clinical question	Threshold μg/g
Caviglia 2014	2014	31	6	15	14	Infl vs non-infl	100
Conroy 2017	2017	6	78	321	5	IBD vs non-IBD	100
El-Badry 2010	2010	7	8	22	2	IBD vs non-IBD	100
Hogberg 2017	2017	7	51	312	3	IBD vs non-IBD	100
Limburg 2000	2000	24	14	67	5	Infl vs non-infl	100

Organic versus non-organic (7 studies)

Study ID	Year of study	TP	FP	TN	FN	Clinical question	Threshold μg/g
Banerjee 2015	2015	14	18	80	7	organic vs non-organic	100
Carroccio 2003	2003	13	3	37	17	organic vs non-organic	100
El-Badry 2010	2010	15	0	20	4	organic vs non-organic	100
Hogberg 2017	2017	12	46	301	14	organic vs other	100
Kennedy 2015	2015	109	74	492	47	organic vs non-organic	100
Pavlidis 2013	2013	73	78	790	21	organic vs non-organic	100
Turvill 2016	2016	20	44	192	6	organic vs non-organic	100

For peer review only

Supplement 11 2x2 table data at 50µg/g threshold of 28 studies considered for exploratory sensitivity analysis

Study ID	Year of study	TP	FP	TN	FN
Alrubaiy 2012	2012	2	18	34	0
Banerjee 2015c*	2015	12	39	59	0
Banerjee 2015i	2015	19	39	59	2
Boyd 2016a	2016	23	117	284	0
Burri 2013a	2013	111	13	249	32
Burri 2013b	2013	55	4	230	72
Carroccio 2003a	2003	11	8	32	0
Carroccio 2003b	2003	11	16	43	0
Carroccio 2003c	2003	19	8	32	11
Caviglia 2014a	2014	24	10	11	0
Caviglia 2014d	2014	38	10	11	7
Conroy 2017a	2017	8	140	259	3
Conroy 2017f	2017	21	127	256	6
Damms 2008a	2008	18	12	44	0
Damms 2008b	2008	16	11	45	2
Damms 2008c	2008	52	13	43	14
Damms 2008d	2008	53	16	40	13
De Sloovere 2017a	2017	46	55	61	0
De Sloovere 2017d	2017	46	30	86	0
De Sloovere 2017h	2017	43	14	102	3
De Sloovere 2017k	2017	40	54	42	0
De Sloovere 2017o	2017	40	45	51	0
De Sloovere 2017t	2017	38	33	63	2
Dhaliwal 2015	2015	126	33	115	18
El-Badry 2010a	2010	9	3	17	0
El-Badry 2010c	2010	9	13	17	0
El-Badry 2010e	2010	19	3	17	0
Hogberg 2017b	2017	9	100	263	1
Hogberg 2017e	2017	20	89	258	6
Jang 2016a	2016	30	1	4	1
Jang 2016b	2016	31	1	4	0
Jang 2016c	2016	25	0	5	6
Kennedy 2015b	2015	88	149	417	3
Kennedy 2015f	2015	123	149	417	33
Kok 2012a	2012	73	149	132	25
Kok 2012b	2012	63	131	150	36
Labaere 2014b	2014	9	1	18	3
Labaere 2014c	2014	9	2	15	2
Labaere 2014d	2014	10	2	17	2
Labaere 2014e	2014	10	6	13	2
Labaere 2014g	2014	10	3	16	2
Li 2006	2006	55	6	54	5
Lizvan 2015	2015	32	1	15	4

For peer review only - http://bmjopen.bmj.com/site/about/guidelines.xhtml

Mowat 2016a	2016	29	421	273	3
Mowat 2016c	2016	76	374	251	25
Otten 2008a	2008	22	12	79	1
Oyaert 2014a	2014	48	17	115	3
Oyaert 2014b	2014	51	37	95	0
Oyaert 2017a	2017	21	22	43	0
Oyaert 2017c	2017	21	18	47	0
Oyaert 2017e	2017	21	14	51	0
Oyaert 2017g	2017	21	27	38	0
Oyaert 2017i	2017	21	27	38	0
Oyaert 2017k	2017	21	22	43	0
Pavlidis 2013a	2013	77	199	669	17
Schoepfer 2008	2008	53	0	30	11
Sostres 2017	2017	27	63	71	10
Tan 2016	2016	15	165	95	0
Tibble 2002	2002	234	71	268	29
Turvill 2012	2012	91	39	482	18

*Letters indicate different 2x2 table data from the same study reference

For peer review only
